# Supplementary material for: Synthesis and Biological Activity of Myricetin Derivatives Containing Pyrazole Piperazine Amide
Source: Int J Mol Sci. 2023 Jun 21;24(13):10442. doi: 10.3390/ijms241310442 (PMC10341855; doi:10.3390/ijms241310442)
Supplement: Supplementary file 1 [file ijms-24-10442-s001.zip › ijms-2451446-supplementary.pdf]

Supplementary Materials

Synthesis and Biological Activity of Myricetin Derivatives Containing Pyrazole Piperazine Amide

Fang Liu, Xiao Cao, Tao Zhang, Li Xing, Zhiling Sun, Wei Zeng, Hui Xin and Wei Xue *

National Key Laboratory of Green Pesticide, Key Laboratory of Green Pesticide and Agricultural Bioengineering, Ministry of Education, Center for R&D of Fine Chemicals of Guizhou University, Guiyang 550025, China; gs.liuf20@gzu.edu.cn (F.L.); gs.xcao20@gzu.edu.cn (X.C.); gs.zt21@gzu.edu.cn (T.Z.); gs.lxing21@gzu.edu.cn (L.X.); gs.zlsun22@gzu.edu.cn (Z.S.); gs.wzeng22@gzu.edu.cn (W.Z.); xinhui1135@163.com (H.X.)

* Correspondence: wxue@gzu.edu.cn

The characterization data of the target compounds including ^{1}H NMR, ^{13}C NMR and HRMS were shown as below.

Data for *5-chloro-1-(4-nitrophenyl)-3-phenyl-1H-pyrazole-4-carboxylic acid* (**b-4**): White solid, yield: 36%. m.p 213.9-214.5°C; 1H NMR (400 MHz, DMSO) δ 13.17 (s, 1H, -COOH), 8.50 – 8.42 (m, 2H, P h), 8.10 – 7.99 (m, 2H, Ph), 7.68 (dd, *J* = 6.6, 3.0 Hz, 2H, Ph), 7.48 – 7.44 (m, 3H, Ph); ^{13}C NMR (101 MHz, DMSO) δ 163.10, 153.95, 147.58, 142.35, 132.17, 131.81, 129.64, 129.40, 128.45, 127.01, 125.26, 111.96; ESI-HRMS calcd for $C_{16}H_{10}O_4N_3Cl$ [M+H]$^+$344.04326, found 344.04385.

Data for *tert-butyl 4-(5-chloro-1,3-diphenyl-1H-pyrazole-4-carbonyl)piperazine-1-carboxylate* (**c-2**): White s olid, yield: 14%. m.p 59.1-60.2°C; ^{1}H NMR (400 MHz, DMSO) δ 7.71 (d, *J* = 7.5 Hz, 2H, Ph), 7.6 7 – 7.54 (m, 6H, Ph), 7.46 (dd, *J* = 14.0, 6.1 Hz, 2H, Ph), 3.66 (d, *J* = 30.0 Hz, 4H, piperazine), 3.15 (dd, *J* = 95.6, 60.6 Hz, 4H, piperazine), 1.39 (s, 9H, -O-C(CH_3)$_3$); ^{13}C NMR (101 MHz, DMS O) δ 162.04, 154.12, 148.98, 137.69, 131.76, 129.84, 129.59, 129.43, 128.35, 127.02, 125.82, 113.93, 79. 78, 46.66, 41.70, 28.44; ESI-HRMS calcd for $C_{25}H_{27}O_3N_4Cl$ [M+H]$^+$467.18444, found 467.18356.

Data for *tert-butyl 4-(5-chloro-3-methyl-1-(4-nitrophenyl)-1H-pyrazole-4-carbonyl)piperazine-1-carboxylate* (**c -3**): White solid, yield: 86%. m.p 148.9-149.8°C; ^{1}H NMR (400 MHz, DMSO) δ 8.43 (d, *J* = 9.0 H z, 2H, Ph), 7.96 (d, *J* = 9.0 Hz, 2H, Ph), 3.56 (d, *J* = 40.8 Hz, 5H, piperazine), 2.98 (d, *J* = 233.8 Hz, 3H, piperazine), 2.26 (s, 3H, pyrazol-CH_3), 1.42 (s, 9H, -O-C(CH_3)$_3$); ^{13}C NMR (101 MHz, D MSO) δ 161.59, 154.24, 149.79, 146.91, 142.59, 125.79, 125.56, 125.27, 116.20, 79.76, 46.84, 41.83, 38. 71, 28.47, 13.17; ESI-HRMS calcd for $C_{20}H_{24}O_5N_5Cl$ [M+H]$^+$450.15387, found 450.15274.

Data for *tert-butyl 4-(5-chloro-1-(4-nitrophenyl)-3-phenyl-1H-pyrazole-4-carbonyl)piperazine-1-carboxylate* (**c -4**): White solid, yield: 40%. m.p 132.1-132.5°C; 1H NMR (400 MHz, DMSO) δ 8.49 – 8.44 (m, 2 H, Ph), 8.09 – 8.04 (m, 2H, Ph), 7.68 (dd, *J* = 8.1, 1.3 Hz, 2H, Ph), 7.53 – 7.45 (m, 3H, Ph), 3.61 (t, *J* = 38.4 Hz, 4H, piperazine), 3.10 (dd, *J* = 150.7, 11.0 Hz, 3H, piperazine), 2.69 (s, 1H, pipera

zine), 1.39 (s, 9H, -O-C(CH_3)$_3$); 1^{3}C NMR (101 MHz, DMSO) δ 161.63, 154.12, 150.03, 147.22, 142. 51, 131.31, 129.80, 129.51, 129.40, 127.11, 126.11, 125.34, 115.28, 79.80, 46.60, 41.73, 38.71, 28.44; ES I-HRMS calcd for $C_{25}H_{26}O_5N_5Cl$ [M+H]$^+$512.16952, found 512.16846.

Data for *tert-butyl 4-(5-chloro-1-(4-chlorophenyl)-3-methyl-1H-pyrazole-4-carbonyl)piperazine-1-carboxylate* (**c-5**): White solid, yield: 65%. m.p 170.5-171.5°C; ^{1}H NMR (400 MHz, DMSO) δ 7.65 (s, 4H, Ph), 3.55 (d, *J* = 35.1 Hz, 5H, piperazine), 3.14 (d, *J* = 102.2 Hz, 3H, piperazine), 2.23 (s, 3H, pyrazol-CH3), 1.41 (s, 9H, -O-C(CH3)3); ^{13}C NMR (101 MHz, DMSO) δ 161.92, 154.24, 151.50, 148.76, 136. 57, 133.64, 129.76, 127.36, 115.00, 79.75, 46.86, 41.86, 28.47, 13.15; ESI-HRMS calcd for $C_{20}H_{25}O_3N_4$ Cl_2 [M+H]$^+$439.12982, found 439.12888.

Data for *tert-butyl 4-(5-chloro-1-(4-chlorophenyl)-3-phenyl-1H-pyrazole-4-carbonyl)piperazine-1-carboxylate* (**c-6**): White solid, yield: 78%. m.p 161.2-162.5°C; ^{1}H NMR (400 MHz, DMSO) δ 7.77 (d, *J* = 8.8 Hz, 2H, Ph), 7.75 – 7.67 (m, 3H, Ph), 7.65 (d, *J* = 6.9 Hz, 2H, Ph), 7.49 – 7.45 (m, 2H, Ph), 3.75 – 3.40 (m, 4H, piperazine), 3.15 (dd, *J* = 93.8, 61.0 Hz, 4H, piperazine), 1.39 (s, 9H, -O-C(CH_3)$_3$); ^{13}C NMR (101 MHz, DMSO) δ 161.90, 154.12, 149.24, 136.51, 134.04, 131.62, 129.87, 129.54, 129.44, 127.53, 127.04, 114.20, 79.79, 46.63, 41.71, 28.44; ESI-HRMS calcd for $C_{25}H_{26}O_3N_4Cl_2$ [M+H]$^+$501.145 47, found 501.14438.

Data for *tert-butyl 4-(5-chloro-3-methyl-1-(pyridin-2-yl)-1H-pyrazole-4-carbonyl)piperazine-1-carboxylate* (**c-7**): White solid, yield: 29%. m.p 124.3-125.1°C; ^{1}H NMR (400 MHz, DMSO) δ 8.58 (dd, *J* = 4.8, 1. 0 Hz, 1H, Pyridine-6-H), 8.07 (td, *J* = 8.0, 1.8 Hz, Pyridine-4-H), 7.75 (d, *J* = 8.2 Hz, 1H, Pyridin e-3-H), 7.53 (dd, *J* = 7.1, 5.1 Hz, 1H, Pyridine-5-H), 3.54 (d, *J* = 53.8 Hz, 4H, piperazine), 3.36 – 2.67 (m, 4H, piperazine), 2.24 (s, 3H, pyrazol-CH_3), 1.41 (s, 9H, -O-C(CH_3)$_3$); ^{13}C NMR (101 MHz, DMSO) δ 161.78, 154.23, 150.89, 148.82 (d, *J* = 12.1 Hz), 139.94, 125.18, 124.25, 118.89, 116.12, 79. 75, 46.90, 41.79, 38.71, 28.47, 13.17; ^{13}C NMR (101 MHz, DMSO) δ 161.78, 154.23, 150.89, 148.88, 148.76, 139.94, 125.18, 124.25, 118.89, 116.12, 79.75, 46.90, 41.79,38.71, 28.47, 13.17; ESI-HRMS calcd for $C_{19}H_{24}O_3N_5Cl$ [M+H]$^+$406.16404, found 406.16251.

Data for *tert-butyl 4-(5-chloro-3-phenyl-1-(pyridin-2-yl)-1H-pyrazole-4-carbonyl)piperazine-1-carboxylate* (**c-8**): White solid, yield: 51%. m.p 45.1-46.2°C; ^{1}H NMR (400 MHz, DMSO) δ 8.64 (dd, *J* = 4.8, 1.1 Hz, 1H, Pyridine-6-H), 8.13 (td, *J* = 7.9, 1.9 Hz, 1H, Pyridine-4-H), 7.90 (d, *J* = 8.1 Hz, 1H, Pyrid ine-3-H), 7.68 (d, *J* = 6.9 Hz, 2H, Ph), 7.59 (dd, *J* = 7.1, 5.3 Hz, 1H, Pyridine-5-H), 7.48 (dq, *J* = 14.3, 7.1 Hz, 3H, Ph), 3.76 – 3.40 (m, 3H, piperazine), 3.34 (s, 1H, piperazine), 3.27 (s, 1H, piper azine), 2.86 (s, 1H, piperazine), 2.69 (s, 2H, piperazine), 1.39 (s, 9H, -O-C(CH_3)$_3$); ^{13}C NMR (101 MHz, DMSO) δ 161.81, 154.10, 150.85, 149.29, 148.89, 140.13, 131.46, 129.70, 129.48, 127.08, 126.24, 124.66, 119.21, 115.22, 79.79, 46.64, 41.69, 38.71, 28.43; ESI-HRMS calcd for $C_{24}H_{26}O_3N_5Cl$ [M+H]$^+$4 68.17969, found 468.17868.

Data for *tert-butyl 4-(5-chloro-1-(4-methoxyphenyl)-3-methyl-1H-pyrazole-4-carbonyl)piperazine-1-carboxylat e* (**c-9**): White solid, yield: 52%. m.p 122.8-123.2°C; ^{1}H NMR (400 MHz, DMSO) δ 7.49 (d, *J* = 8.9 Hz, 2H, Ph), 7.09 (d, *J* = 8.9 Hz, 2H, Ph), 3.83 (s, 3H, -OCH_3), 3.57 (t, *J* = 29.6 Hz, 6H, piperaz ine), 2.98 (d, *J* = 235.8 Hz, 2H, piperazine), 2.21 (s, 3H, pyrazol-CH_3), 1.41 (s, 9H, -O-C(CH_3)$_3$); ^{13}C NMR (101 MHz, DMSO) δ 162.19, 159.74, 154.24, 147.97, 130.68, 127.35, 125.29, 114.75, 114.17,

79.74, 55.98, 38.71, 28.47, 13.17; ESI-HRMS calcd for $C_{21}H_{27}O_4N_4Cl$ $[M+H]^+$435.17936, found 435.178 41.

Data for *tert-butyl 4-(5-chloro-1,3-dimethyl-1H-pyrazole-4-carbonyl)piperazine-1-carboxylate* (**c-10**): White solid, yield: 54%. m.p 118.4-119.0°C; ^{1}H NMR (400 MHz, DMSO) δ 3.73 (s, 3H, pyrazol-N-CH_3), 3.54 (d, *J* = 11.6 Hz, 3H, piperazine), 3.34 (s, 3H, piperazine), 2.69 (s, 2H, piperazine), 2.12 (s, 3H, pyrazol-CH_3), 1.41 (s, 9H, -O-C(CH_3)$_3$); ^{13}C NMR (101 MHz, DMSO) δ 162.51, 154.23, 146.46, 125.32, 112.67, 79.72, 38.71, 36.42, 28.47, 13.09; ESI-HRMS calcd for $C_{15}H_{23}O_3N_4Cl$ $[M+H]^+$343.15314, found 343.15244.

Data for *3-(3-(4-(5-chloro-3-methyl-1-phenyl-1H-pyrazole-4-carbonyl)piperazin-1-yl)propoxy)-5,7-dimethoxy-2-(3,4,5-trimethoxyphenyl)-4H-chromen-4-one* (**D1**): White solid, yield: 46%. m.p. 181.2-181.6°C; ^{1}H NMR (400 MHz, DMSO) δ 7.61 – 7.56 (m, 4H, Ph-H), 7.52 (d, *J* = 2.9 Hz, 1H, Ph-H), 7.36 (s, 2H, Ph-H), 6.84 (d, *J* = 2.2 Hz, 1H, Ph-H), 6.50 (d, *J* = 2.2 Hz, 1H, Ph-H), 3.99 (t, *J* = 6.1 Hz, 2H, -O-$\underline{CH_2}$-CH_2-CH_2-N), 3.90 (s, 3H, Ph-OCH_3), 3.86 (d, *J* = 12.3 Hz, 9H, Ph-OCH_3), 3.74 (s, 3H, Ph-OCH_3), 3.49 (d, *J* = 60.6 Hz, 4H, piperazine), 2.39 – 2.34 (m, 2H, -O-CH_2-CH_2-$\underline{CH_2}$-N), 2.30 (s, 4H, piperazine), 2.21 (s, 3H, pyrazol-CH_3), 1.83 – 1.76 (m, 2H, -O-CH_2-$\underline{CH_2}$-CH_2-N); ^{13}C NMR (101 MHz, DMSO) δ 172.67, 164.21, 161.67, 160.74, 158.65, 153.15, 152.10, 148.18, 140.43, 139.80, 137.77, 129.73, 129.19, 126.11, 125.65, 124.93, 115.00, 108.92, 106.29, 96.40, 93.56, 70.69, 60.65, 56.57, 56.52, 54.88, 53.83, 52.88, 47.10, 41.87, 27.63, 13.14; ESI-HRMS calcd for $C_{38}H_{42}O_9N_4Cl$ $[M+H]^+$733.26348, found 733.26440.

Data for *3-(3-(4-(5-chloro-1,3-diphenyl-1H-pyrazole-4-carbonyl)piperazin-1-yl)propoxy)-5,7-dimethoxy-2-(3,4,5-trimethoxyphenyl)-4H-chromen-4-one* (**D2**): White solid, yield: 9%. m.p. 99.9-101.1°C; ^{1}H NMR (400 MHz, DMSO) δ 7.72 – 7.69 (m, 2H, Ph-H), 7.62 (ddd, *J* = 25.5, 12.1, 4.4 Hz, 5H, Ph-H), 7.50 – 7.41 (m, 3H, Ph-H), 7.34 (s, 2H, Ph-H), 6.84 (d, *J* = 2.2 Hz, 1H, Ph-H), 6.49 (d, *J* = 2.2 Hz, 1H, Ph-H), 3.95 (t, *J* = 6.1 Hz, 2H, -O-CH_2-CH_2-CH_2-N), 3.90 (s, 3H, Ph-OCH_3), 3.85 (d, *J* = 6.9 Hz, 9H, Ph-OCH_3), 3.73 (s, 3H, Ph-OCH_3), 3.62 (d, *J* = 59.6 Hz, 2H, piperazine), 3.25 (d, *J* = 27.4 Hz, 2H, piperazine), 2.34 (s, 1H, piperazine), 2.31 – 2.27 (m, 2H, -O-CH_2-CH_2-CH_2-N), 2.18 (s, 2H, piperazine), 1.85 (s, 1H, piperazine), 1.75 (dd, *J* = 13.5, 6.5 Hz, 2H, -O-CH_2-CH_2-CH_2-N); ^{13}C NMR (101 MHz, DMSO) δ 172.65, 164.21, 161.70, 160.74, 158.64, 153.14, 152.09, 148.80, 140.39, 139.79, 137.71, 131.80, 129.84, 129.56, 129.37, 126.92, 126.09, 125.99, 125.81, 114.20, 108.91, 106.26, 96.39, 93.56, 70.61, 60.65, 56.56, 54.81, 53.18, 52.53, 46.85, 41.83, 27.53; ESI-HRMS calcd for $C_{43}H_{44}O_9N_4Cl$ $[M+H]^+$795.27913, found 795.27673.

Data for *3-(3-(4-(5-chloro-3-methyl-1-(4-nitrophenyl)-1H-pyrazole-4-carbonyl)piperazin-1-yl)propoxy)-5,7-dimethoxy-2-(3,4,5-trimethoxyphenyl)-4H-chromen-4-one* (**D3**): Yellow solid, yield: 17%. m.p. 113.8-115.2°C; ^{1}H NMR (400 MHz, DMSO) δ 8.42 (d, *J* = 9.1 Hz, 2H, Ph), 7.95 (d, *J* = 9.1 Hz, 2H, Ph), 7.36 (s, 2H, Ph), 6.84 (d, *J* = 2.1 Hz, 1H, Ph), 6.50 (d, *J* = 2.1 Hz, 1H, Ph), 3.99 (t, *J* = 6.0 Hz, 2H, -O-$\underline{CH_2}$-$\underline{CH_2}$-$\underline{CH_2}$-N), 3.90 (s, 3H, Ph-$\underline{OCH_3}$), 3.86 (d, *J* = 12.7 Hz, 9H, Ph-OCH_3), 3.74 (s, 3H, Ph-OCH_3), 3.51 (d, *J* = 58.9 Hz, 4H, piperazine), 2.41 – 2.35 (m, 2H, -O-CH_2-CH_2-$\underline{CH_2}$-N), 2.31 (s, 4H, piperazine), 2.24 (s, 3H, pyrazol-CH_3), 1.84 – 1.76 (m, 2H, -O-CH_2-$\underline{CH_2}$-CH_2-N); ^{13}C NMR (101 MHz, DMSO) δ 172.67, 164.22, 161.19, 160.74, 158.65, 153.15, 152.09, 149.58, 146.89, 142.60, 140.42, 139.80, 126.11, 125.74, 125.35, 125.26, 116.49, 108.92, 106.28, 96.40, 93.56, 70.74, 70.70, 60.66, 56.57, 56.52, 54.88, 53.82, 52.82, 47.08, 41.94, 27.63, 13.14; ESI-HRMS calcd for $C_{38}H_{41}O_{11}N_5Cl$ $[M+H]^+$778.24856, found 778.25000.

Data for *3-(3-(4-(5-chloro-1-(4-nitrophenyl)-3-phenyl-1H-pyrazole-4-carbonyl)piperazin-1-yl)propoxy)-5,7-dimethoxy-2-(3,4,5-trimethoxyphenyl)-4H-chromen-4-one* (**D4**)*:* White solid, yield: 26%. m.p. 150.5-152.8°C; ^{1}H NMR (400 MHz, DMSO) δ 8.55 -8.39 (m, 2H, -Ph), 8.11 - 8.03 (m, 2H, -Ph), 7.70 - 7.63 (m, 2H, -Ph), 7.55

- 7.40 (m, 3H, -Ph), 7.34 (s, 2H, -Ph), 6.84 (d, *J* = 2.1 Hz, 1H, -Ph), 6.49 (d, *J* = 2.2 Hz, 1H, -Ph), 3.94 (t, *J* = 6.1 Hz, 2H, -O-CH_2-CH_2-CH_2-N), 3.89 (s, 3H, Ph-OCH_3), 3.84 (d, *J* = 8.2 Hz, 9H, Ph-OCH_3), 3.73 (s, 3H, Ph-OCH_3), 3.62 (d, *J* = 40.0 Hz, 2H, piperazine), 3.28 (d, *J* = 35.5 Hz, 2H, piperazine), 2.35 (s, 1H, piperazine), 2.30 (s, 2H, -O-CH_2-CH_2-CH_2-N), 2.21 (d, *J* = 10.7 Hz, 2H, piperazine), 1.87 (s, 1H, piperazine), 1.80 - 1.70 (m, 2H, -O-CH_2-CH_2-CH_2-N); ^{13}C NMR (101 MHz, DMSO) δ 172.69, 164.23, 161.29, 160.71, 158.64, 153.13, 152.10, 149.89, 147.18, 142.51, 140.36, 139.75, 131.31, 129.77, 129.47, 127.02, 126.44, 126.07, 125.33, 115.51, 108.87, 106.19, 96.38, 93.54, 70.59, 60.65, 56.53, 54.81, 53.20, 52.50, 46.81, 41.83, 27.63; ESI-HRMS calcd for $C_{43}H_{43}O_{11}N_5Cl$ [M+H]$^+$840.26421, found 840.26147.

Data for *3-(3-(4-(5-chloro-1-(4-chlorophenyl)-3-methyl-1H-pyrazole-4-carbonyl)piperazin-1-yl)propoxy)-5,7-dimethoxy-2-(3,4,5-trimethoxyphenyl)-4H-chromen-4-one (***D5***)*: White solid, yield: 4 %. m.p. 165.7-166.5 °C; ^{1}H NMR (400 MHz, DMSO) *δ* 7.64 (s, 4H, Ph-H), 7.36 (s, 2H, Ph-H), 6.84 (d, *J* = 2.2 Hz, 1H, Ph-H), 6.50 (d, *J* = 2.2 Hz, 1H, Ph-H), 3.99 (t, *J* = 6.1 Hz, 2H, -O-CH_2-CH_2-CH_2-N), 3.90 (s, 3H, Ph-OCH_3), 3.86 (d, *J* = 12.2 Hz, 9H, Ph-OCH_3), 3.74 (s, 3H, Ph-OCH_3), 3.49 (d, *J* = 53.9 Hz, 4H, piperazine), 2.43 – 2.34 (m, 2H, -O-CH_2-CH_2-CH_2-N), 2.30 (s, 4H, piperazine), 2.21 (s, 3H, pyrazol-CH_3), 1.85 – 1.72 (m, 2H, -O-CH_2-CH_2-CH_2-N); ^{13}C NMR (101 MHz, DMSO) *δ* 172.67, 164.21, 161.52, 160.74, 158.65, 153.15, 152.10, 148.53, 140.43, 139.80, 136.59, 133.61, 129.76, 127.33, 126.11, 125.08, 115.29, 108.92, 106.29, 96.40, 93.56 , 70.70, 60.65, 56.57, 56.52, 54.88, 53.87, 52.94, 47.02, 41.93, 27.63, 13.12; ESI-HRMS calcd for $C_{38}H_{41}O_9N_4Cl_2$ [M+H]$^+$767.22451, found 767.22571.

Data for *3-(3-(4-(5-chloro-1-(4-chlorophenyl)-3-phenyl-1H-pyrazole-4-carbonyl)piperazin-1-yl)propoxy)-5,7-dimethoxy-2-(3,4,5-trimethoxyphenyl)-4H-chromen-4-one* (**D6**): White solid, yield: 29% **.**m.p 126.7-128.2°C; ^{1}H NMR (400 MHz, DMSO) δ 7.76 (d, *J* = 8.7 Hz, 2H, Ph), 7.70 – 7.65 (m, 4H, Ph), 7.49 – 7.44 (m, 3H, Ph), 7.34 (s, 2H, Ph), 6.84 (d, *J* = 2.1 Hz, 1H, Ph), 6.50 (d, *J* = 2.1 Hz, 1H, Ph), 3.95 (t, *J* = 6.1 Hz, 2H, -O-CH_2-CH_2-CH_2-N), 3.90 (s, 3H, Ph-OCH_3), 3.86 – 3.77 (m, 9H, Ph-OCH_3), 3.73 (s, 3H, Ph-OCH_3), 3.60 (d, *J* = 58.6 Hz, 2H, piperazine), 3.20 (s, 2H, piperazine), 2.33 (s, 1H, piperazine), 2.31 – 2.27 (m, 2H, -O-CH_2-CH_2-CH_2-N), 2.12 (d, *J* = 32.8 Hz, 2H, piperazine), 1.85 (s, 1H, piperazine), 1.81 – 1.68 (m, 2H, -O-CH_2-CH_2-CH_2-N); ^{13}C NMR (101 MHz) δ 172.66, 164.22, 161.62, 160.76, 158.65, 153.15, 152.10, 149.04, 139.86, 136.56, 133.99, 131.73, 131.67, 129.86, 129.48, 129.38, 127.49, 126.95, 126.90, 114.49, 108.94, 106.33, 96.41, 93.58, 70.64, 60.66, 56.59, 54.80, 53.21, 52.52, 46.84, 41.85, 27.53; ESI-HRMS calcd for $C_{43}H_{43}O_9N_4Cl_2$ [M+H]$^+$829.24016, found 829.24078.

Data for *3-(3-(4-(5-chloro-3-methyl-1-(pyridin-2-yl)-1H-pyrazole-4-carbonyl)piperazin-1-yl)propoxy)-5,7-dimethoxy-2-(3,4,5-trimethoxyphenyl)-4H-chromen-4-one* (**D7**)*:* White solid, yield: 31%. m.p 150.1-151.2°C; ^{1}H NMR (400 MHz, DMSO) δ 8.60 – 8.55 (m, 1H, pyridine-6-H), 8.09 – 8.04 (m, 1H, pyridine-4-H), 7.75 (d, *J* = 8.1 Hz, 1H, pyridine-3-H), 7.52 (ddd, *J* = 7.5, 4.9, 0.9 Hz, 1H, pyridine-5-H), 7.36 (s, 2H, Ph), 6.84 (d, *J* = 2.1 Hz, 1H, Ph), 6.50 (d, *J* = 2.1 Hz, 1H, Ph), 3.99 (t, *J* = 6.1 Hz, 2H, -O-CH_2-CH_2-CH_2-N), 3.90 (s, 3H, Ph-OCH_3), 3.86 (d, *J* = 12.0 Hz, 9H, Ph-OCH_3), 3.74 (s, 3H, Ph-OCH_3), 3.58 (s, 2H, piperazine), 3.28 (s, 2H, piperazine), 2.39 – 2.34 (m, 2H, -O-CH_2-CH_2-CH_2-N), 2.31 (s, 4H, piperazine), 2.23 (s, 3H, pyrazol-CH_3), 1.84 – 1.75 (m, 2H, -O-CH_2-CH_2-CH_2-N); ^{13}C NMR (101 MHz, DMSO) δ 172.66, 164.21, 161.38, 160.74, 158.64, 153.15, 150.91, 148.76, 148.68, 140.42, 139.93, 139.80, 126.11, 124.99, 124.22, 118.88, 116.37, 108.93, 106.29, 96.39, 93.56, 70.68, 60.65, 56.57, 56.52, 54.87, 53.79, 52.87, 47.06, 41.88, 27.63, 13.13; ESI-HRMS calcd for $C_{37}H_{41}O_9N_5Cl$ [M+H]$^+$734.25873, found 734.25623.

Data for *3-(3-(4-(5-chloro-3-phenyl-1-(pyridin-2-yl)-1H-pyrazole-4-carbonyl)piperazin-1-yl)propoxy)-5,7-dimethoxy-2-(3,4,5-trimethoxyphenyl)-4H-chromen-4-one* (**D8**): White solid, yield: 42%. m.p 82.7-84.6°C; ^{1}H NMR (400 MHz, DMSO) δ 8.63 (dd, *J* = 4.8, 1.1 Hz, 1H, Pyridine-6-H), 8.13 (td, *J* = 7.9, 1.9 Hz, 1H, Pyridine-

4-H), 7.90 (d, *J* = 8.1 Hz, 1H, Pyridine-3-H), 7.67 (d, *J* = 6.9 Hz, 2H, Ph), 7.58 (ddd, *J* = 7.5, 4.9, 0.9 Hz, 1H, Pyridine-5-H), 7.52 – 7.43 (m, 3H, Ph), 7.34 (s, 2H, Ph), 6.83 (d, *J* = 2.2 Hz, 1H, Ph), 6.49 (d, *J* = 2.2 Hz, 1H, Ph), 3.95 (t, *J* = 6.1 Hz, 2H, -O-$\underline{CH_2}$-CH_2-CH_2-N), 3.90 (s, 3H, Ph-OCH_3), 3.85 (d, *J* = 6.6 Hz, 9H, Ph-OCH_3), 3.73 (s, 3H, Ph-OCH_3), 3.62 (d, *J* = 60.6 Hz, 2H, piperazine), 3.24 (d, *J* = 33.8 Hz, 2H, piperazine), 2.35 (s, 1H, piperazine), 2.29 (t, *J* = 7.1 Hz, 2H, -O-CH_2-CH_2-$\underline{CH_2}$-N), 2.19 (s, 2H, piperazine), 1.85 (s, 1H, piperazine), 1.79 – 1.67 (m, 2H, -O-CH_2-$\underline{CH_2}$-CH_2-N); ^{13}C NMR (101 MHz, DMSO) δ 172.65, 164.21, 161.47, 160.73, 158.64, 153.14, 150.87, 149.13, 148.89, 140.39, 140.11, 139.79, 131.50, 129.64, 129.42, 126.99, 126.09, 119.20, 115.46, 108.91, 106.26, 96.39, 93.56, 70.60, 60.64, 56.55, 54.80, 53.15, 52.52, 46.84, 41.82, 27.53; ESI-HRMS calcd for $C_{42}H_{43}O_9N_5Cl$ $[M+H]^+$796.27438, found 796.27173.

Data for *3-(3-(4-(5-chloro-1-(4-methoxyphenyl)-3-methyl-1H-pyrazole-4-carbonyl)piperazin-1-yl)propoxy)-5,7-dimethoxy-2-(3,4,5-trimethoxyphenyl)-4H-chromen-4-one* (**D9**): White solid, yield: 64%. m.p 170.2-171.8°C; ^{1}H NMR (400 MHz, DMSO) δ 7.43 (d, *J* = 9.0 Hz, 2H, Ph-H), 7.32 (s, 2H, Ph-H), 7.05 (d, *J* = 9.0 Hz, 2H, Ph-H), 6.79 (d, *J* = 2.3 Hz, 1H, Ph-H), 6.45 (d, *J* = 2.3 Hz, 1H, Ph-H), 3.95 (t, *J* = 6.2 Hz, 2H, -O-$\underline{CH_2}$-CH_2-CH_2-N), 3.86 (s, 3H, Ph-OCH_3), 3.82 (d, *J* = 14.6 Hz, 9H, Ph-OCH_3), 3.78 (s, 3H, Ph-OCH_3), 3.70 (s, 3H, Ph-OCH_3), 3.42 (d, *J* = 101.3 Hz, 4H, piperazine), 2.32 (t, *J* = 7.3 Hz, 2H, -O-CH_2-CH_2-$\underline{CH_2}$-N), 2.25 (s, 4H, piperazine), 2.15 (s, pyrazol-CH_3), 1.78 – 1.72 (m, 2H, -O-CH_2-$\underline{CH_2}$-CH_2-N); ^{13}C NMR (101 MHz,) δ 172.72, 164.28, 161.86, 160.83, 159.80, 158.71, 153.22, 152.16, 147.77, 140.50, 139.93, 130.78, 127.37, 126.18, 125.12, 114.83, 114.54, 109.02, 106.43, 96.47, 93.64, 70.77, 60.72, 56.66, 56.58, 56.05, 54.93, 53.83, 52.97, 47.23, 41.89, 27.70, 13.18; ESI-HRMS calcd for $C_{39}H_{44}O_{10}N_4Cl$ $[M+H]^+$763.27405, found 763.27185.

Data for *3-(3-(4-(5-chloro-1,3-dimethyl-1H-pyrazole-4-carbonyl)piperazin-1-yl)propoxy)-5,7-dimethoxy-2-(3,4,5-trimethoxyphenyl)-4H-chromen-4-one* (**D10)**: White solid, yield: 32%. m.p 148.4-150.2°C; ^{1}H NMR (400 MHz, DMSO) δ 7.36 (s, 2H, Ph-H), 6.84 (d, *J* = 2.2 Hz, 1H, Ph-H), 6.50 (d, *J* = 2.2 Hz, 1H, Ph-H), 3.98 (t, *J* = 6.1 Hz, 2H, -O-$\underline{CH_2}$-CH_2-CH_2-N), 3.90 (s, 3H, Ph-OCH_3), 3.86 (d, *J* = 11.4 Hz, 9H, Ph-OCH_3), 3.74 (s, 3H, -CH_3), 3.73 (s, 3H, Ph-OCH_3), 3.51 (s, 2H, piperazine), 3.27 (s, 2H, piperazine), 2.34 (t, *J* = 7.2 Hz, 2H, -O-CH_2-CH_2-$\underline{CH_2}$-N), 2.26 (s, 4H, piperazine), 2.10 (s, 3H, pyrazol-CH_3), 1.79 (dd, *J* = 13.5, 6.6 Hz, 2H, -O-CH_2-$\underline{CH_2}$-CH_2-N); ^{13}C NMR (101 MHz, DMSO) δ 172.68, 164.21, 162.12, 160.73, 158.65, 153.14, 152.11, 146.22, 140.40, 139.75, 126.10, 125.09, 112.91, 108.90, 106.25, 96.39, 93.55, 70.68, 60.64, 56.55, 56.53, 54.89, 53.69, 53.15, 47.15, 41.89, 36.39, 27.59, 13.04; ESI-HRMS calcd for $C_{33}H_{40}O_9N_4Cl$ $[M+H]^+$671.24783, found 671.24890.

Data for *3-(4-(4-(5-chloro-3-methyl-1-phenyl-1H-pyrazole-4-carbonyl)piperazin-1-yl)butoxy)-5,7-dimethoxy-2-(3,4,5-trimethoxyphenyl)-4H-chromen-4-one* (**D11**): Yellow solid, yield: 35%. m.p 128.9-129.7°C; ^{1}H NMR (400 MHz, DMSO) δ 7.62 – 7.49 (m, 5H, Ph-H), 7.38 (s, 2H, Ph-H), 6.84 (d, *J* = 2.2 Hz, 1H, Ph-H), 6.49 (d, *J* = 2.2 Hz, 1H, Ph-H), 3.96 (t, *J* = 6.2 Hz, 2H, -O-$\underline{CH_2}$-CH_2-CH_2-CH_2-N), 3.90 (s, 3H, Ph-OCH_3), 3.86 (d, *J* = 11.5 Hz, 9H, Ph-OCH_3), 3.74 (s, 3H, Ph-OCH_3), 3.58 (s, 4H, piperazine), 2.33 (s, 3H, piperazine), 2.27 (t, *J* = 6.8 Hz, 2H, -O-CH_2-CH_2-CH_2-$\underline{CH_2}$-N), 2.25 (s, 1H, piperazine), 2.22 (s, 3H, pyrazol-CH_3), 1.70 – 1.59 (m, 2H, -O-CH_2-$\underline{CH_2}$-CH_2-CH_2-N), 1.55 – 1.44 (m, 2H, -O-CH_2-CH_2-$\underline{CH_2}$-CH_2-N); ^{13}C NMR (101 MHz, DMSO) δ 172.69, 164.20, 161.67, 160.72, 158.63, 153.12, 152.01, 148.19, 140.41, 139.76, 137.76, 129.74, 129.20, 126.13, 125.64, 124.94, 115.01, 108.90, 106.18, 96.38, 93.54, 72.03, 60.64, 57.66, 56.51, 53.80, 52.90, 47.15, 41.95, 27.99, 23.23, 13.14; ESI-HRMS calcd for $C_{39}H_{44}O_9N_4Cl$ $[M+H]^+$747.27913, found 747.27985.

Data for *3-(4-(4-(5-chloro-3-methyl-1-(4-nitrophenyl)-1H-pyrazole-4-carbonyl)piperazin-1-yl)butoxy)-5,7-dimethoxy-2-(3,4,5-trimethoxyphenyl)-4H-chromen-4-one* (**D12**): Yellow solid, yield: 11%. m.p. 130.4-131.6°C; ^{1}H NMR (400 MHz, DMSO) δ 8.48 – 8.36 (m, 2H, Ph-H), 8.02 – 7.90 (m, 2H, Ph-H), 7.38 (s, 2H, Ph-H), 6.84 (d, *J* = 1.9 Hz, 1H, Ph-H), 6.49 (d, *J* = 2.1 Hz, 1H, Ph-H), 3.96 (t, *J* = 6.2 Hz, 2H, -O-$\underline{CH_2}$-CH_2-CH_2-CH_2-N), 3.90 (s, 3H, Ph-OCH_3), 3.86 (d, *J* = 12.3 Hz, 9H, Ph-OCH_3), 3.74 (s, 3H, Ph-OCH_3), 3.51 (d, *J* = 59.6 Hz, 4H,

piperazine), 2.34 (s, 2H, piperazine), 2.28 (d, *J* = 6.8 Hz, 2H, -O-CH_2-CH_2-CH_2-$\underline{CH_2}$-N), 2.25 (s, 2H, piperazine), 2.25 (s, 3H, pyrazol-CH_3), 1.64 (dd, *J* = 14.1, 6.5 Hz, 2H, -O-CH_2-$\underline{CH_2}$-CH_2-CH_2-N), 1.51 (dd, *J* = 14.0, 7.1 Hz, 2H, -O-CH_2-CH_2-$\underline{CH_2}$-CH_2-N); ^{13}C NMR (101 MHz, DMSO) δ 172.69, 164.20, 161.18, 160.71, 158.63, 153.12, 152.01, 149.59, 146.88, 142.60, 140.42, 139.76, 126.12, 125.72, 125.35, 125.27, 116.51, 108.89, 106.17, 96.37, 93.54, 72.03, 60.65, 57.65, 56.52, 53.81, 52.88, 47.08, 41.96, 27.99, 23.23, 13.15; ESI-HRMS calcd for $C_{39}H_{43}O_{11}N_5Cl$ $[M+H]^+$792.26421, found 792.26129.

Data for *3-(4-(4-(5-chloro-1-(4-nitrophenyl)-3-phenyl-1H-pyrazole-4-carbonyl)piperazin-1-yl)butoxy)-5,7-dimethoxy-2-(3,4,5-trimethoxyphenyl)-4H-chromen-4-one* (**D13**): Yellow solid, yield: 31%. m.p. 112.9-114.3°C; ^1H NMR (400 MHz, DMSO) δ 8.49 – 8.45 (m, 2H, Ph-H), 8.10 – 8.06 (m, 2H, Ph-H), 7.67 (dd, *J* = 8.1, 1.3 Hz, 2H, Ph-H), 7.53 – 7.45 (m, 3H, Ph-H), 7.37 (s, 2H, Ph-H), 6.84 (d, *J* = 2.2 Hz, 1H, Ph-H), 6.49 (d, *J* = 2.2 Hz, 1H, Ph-H), 3.93 (t, *J* = 6.3 Hz, 2H, -O-$\underline{CH_2}$-CH_2-CH_2-CH_2-N), 3.90 (s, 3H, Ph-OCH_3), 3.85 (d, *J* = 7.8 Hz, 9H, Ph-OCH_3), 3.73 (s, 3H, Ph-OCH_3), 3.64 (d, *J* = 38.2 Hz, 2H, piperazine), 3.29 (d, *J* = 36.8 Hz, 2H, piperazine), 2.41 – 2.21 (m, 3H, piperazine), 2.18 (d, *J* = 7.1 Hz, 2H, -O-CH_2-CH_2-CH_2-$\underline{CH_2}$-N), 1.86 (s, 1H, piperazine), 1.60 (dd, *J* = 14.1, 6.6 Hz, 2H, -O-CH_2-$\underline{CH_2}$-CH_2-CH_2-N), 1.45 (dd, *J* = 13.8, 6.9 Hz, 2H, -O-CH_2-CH_2-$\underline{CH_2}$-CH_2-N); ^{13}C NMR (101 MHz, DMSO) δ 172.68, 164.20, 161.27, 160.71, 158.62, 153.11, 151.99, 149.90, 147.19, 142.52, 140.41, 139.75, 131.34, 129.46, 127.03, 126.40, 126.11, 126.06, 125.34, 115.57, 108.89, 106.16, 96.37, 93.53, 72.00, 60.64, 57.58, 56.51, 53.20, 52.54, 46.83, 41.90, 27.95, 23.15; ESI-HRMS calcd for $C_{44}H_{45}O_{11}N_5Cl$ $[M+H]^+$854.27986, found 854.27777.

Data for *3-(4-(4-(5-chloro-1-(4-chlorophenyl)-3-methyl-1H-pyrazole-4-carbonyl)piperazin-1-yl)butoxy)-5,7-dimethoxy-2-(3,4,5-trimethoxyphenyl)-4H-chromen-4-one* (**D14**): Yellow solid, yield: 10%. m.p. 88.5-89.9°C; ^1H NMR (400 MHz, DMSO) δ 7.65 (s, 4H, Ph-H), 7.38 (s, 2H, Ph-H), 6.85 (s, 1H, Ph-H), 6.50 (d, *J* = 1.6 Hz, 1H, Ph-H), 3.96 (t, *J* = 6.2 Hz, 2H, -O-$\underline{CH_2}$-CH_2-CH_2-CH_2-N), 3.90 (s, 3H, Ph-OCH_3), 3.86 (d, *J* = 11.6 Hz, 9H, Ph-OCH_3), 3.74 (s, 3H, Ph-OCH_3), 3.52 (d, *J* = 40.0 Hz, 4H, piperazine), 2.31 (d, *J* = 15.2 Hz, 3H, piperazine), 2.29 – 2.25 (m, 2H, -O-CH_2-CH_2-CH_2-$\underline{CH_2}$-N), 2.25 (s, 1H, piperazine), 2.22 (s, 3H, pyrazol-CH_3), 1.63 (dd, *J* = 14.0, 6.5 Hz, 2H, -O-CH_2-$\underline{CH_2}$-CH_2-CH_2-N), 1.50 (d, *J* = 6.8 Hz, 2H, -O-CH_2-CH_2-$\underline{CH_2}$-CH_2-N); ^{13}C NMR (101 MHz, DMSO) δ 172.69, 164.20, 161.51, 160.72, 158.63, 153.12, 152.01, 148.54, 140.41, 139.76, 136.58, 133.61, 129.77, 127.32, 126.12, 125.09, 115.30, 108.89, 106.18, 96.38, 93.54, 72.03, 60.64, 57.65, 56.51, 53.81, 52.87, 47.11, 41.92, 27.98, 23.23, 13.13; ESI-HRMS calcd for $C_{39}H_{43}O_9N_4Cl_2$ $[M+H]^+$781.24016, found 781.23767.

Data for *3-(4-(4-(5-chloro-1-(4-chlorophenyl)-3-phenyl-1H-pyrazole-4-carbonyl)piperazin-1-yl)butoxy)-5,7-dim ethoxy-2-(3,4,5-trimethoxyphenyl)-4H-chromen-4-one* (**D15**)**:** White solid, yield: 46%. m.p 101.8-102.8°C; ^1H NMR (400 MHz, DMSO) *δ* 7.79 – 7.75 (m, 2H, Ph-H), 7.71 – 7.63 (m, 4H, Ph-H), 7.50 – 7.4 2 (m, 3H, Ph-H), 7.36 (s, 2H, Ph-H), 6.84 (d, *J* = 2.2 Hz, 1H, Ph-H), 6.49 (d, *J* = 2.2 Hz, 1H, Ph-H), 3.94 (d, *J* = 6.2 Hz, 2H, -O-$\underline{CH_2}$-CH_2-CH_2-CH_2-N), 3.90 (s, 3H, Ph-OCH_3), 3.84 (d, *J* = 6.4 Hz, 9H, Ph-OCH_3), 3.73 (s, 3H, Ph-OCH_3), 3.68 – 3.56 (m, 2H, piperazine), 3.26 (d, *J* = 35.0 Hz, 2H, piperazine), 2.31 (d, *J* = 25.5 Hz, 3H, piperazine), 2.21 – 2.17 (m, 2H, -O-CH_2-CH_2-CH_2-$\underline{CH_2}$-N), 1. 84 (s, 1H, piperazine), 1.65 – 1.57 (m, 2H, -O-CH_2-$\underline{CH_2}$-CH_2-CH_2-N), 1.45 (dd, *J* = 13.7, 7.0 Hz, 2 H, -O-CH_2-CH_2-$\underline{CH_2}$-CH_2-N); ^{13}C NMR (101 MHz, DMSO) δ 172.73, 164.27, 161.62, 160.81, 158.69, 153.19, 152.05, 149.16, 140.48, 139.92, 136.61, 134.07, 131.74, 129.92, 129.53, 129.43, 127.54, 127.02, 1 26.18, 114.59, 109.00, 106.35, 96.45, 93.64, 72.09, 60.71, 57.65, 56.62, 56.58, 53.23, 52.62, 46.92, 41.94, 28.02, 23.22; ESI-HRMS calcd for $C_{44}H_{45}O_9N_4Cl_2$ $[M+H]^+$843.25581, found 843.25671.

Data for *3-(4-(4-(5-chloro-3-methyl-1-(pyridin-2-yl)-1H-pyrazole-4-carbonyl)piperazin-1-yl)butoxy)-5,7-dimethoxy-2-(3,4,5-trimethoxyphenyl)-4H-chromen-4-one* (**D16**): White solid, yield: 20%. m.p 111.1-112.3°C;

^{1}H NMR (400 MHz, DMSO) δ 8.58 (dd, *J* = 4.8, 1.0 Hz, 1H, pyridine-6-H), 8.09 – 8.04 (m, 1H, pyridine-4-H), 7.75 (d, *J* = 8.1 Hz, 1H, pyridine-3-H), 7.52 (dd, *J* = 7.0, 5.3 Hz, 1H, pyridine-5-H), 7.38 (s, 2H, Ph-H), 6.84 (t, *J* = 3.5 Hz, 1H, Ph-H), 6.50 – 6.47 (m, 1H, Ph-H), 3.96 (t, *J* = 6.1 Hz, 2H, -O-$\underline{CH_2}$-CH_2-CH_2-CH_2-N), 3.90 (s, 3H, Ph-OCH_3), 3.85 (d, *J* = 11.1 Hz, 9H, Ph-OCH_3), 3.74 (s, 3H, Ph-OCH_3), 3.59 (s, 2H, piperazine), 3.30 (s, 2H, piperazine), 2.36 – 2.24 (m, 6H, piperazine(4H), -O-CH_2-CH_2-CH_2-CH_2-N(2H)), 2.23 (s, 3H, pyrazol-CH_3), 1.63 (dd, *J* = 13.9, 6.6 Hz, 2H, -O-CH_2-$\underline{CH_2}$-CH_2-CH_2-N), 1.50 (dd, *J* = 14.1, 7.1 Hz, 2H, -O-CH_2-CH_2-$\underline{CH_2}$-CH_2-N); ^{13}C NMR (101 MHz, DMSO) δ 172.69, 164.21, 161.38, 160.74, 158.64, 153.13, 150.92, 148.76, 148.67, 140.42, 139.93, 139.81, 126.12, 124.99, 124.22, 118.87, 116.41, 108.92, 106.24, 96.39, 93.56, 72.04, 60.64, 57.66, 56.54, 53.80, 52.92, 47.10, 41.91, 27.99, 23.24, 13.13; ESI-HRMS calcd for $C_{38}H_{43}O_9N_5Cl$ $[M+H]^+$748.27438, found 748.27240.

Data for *3-(4-(4-(5-chloro-3-phenyl-1-(pyridin-2-yl)-1H-pyrazole-4-carbonyl)piperazin-1-yl)butoxy)-5,7-dimethoxy-2-(3,4,5-trimethoxyphenyl)-4H-chromen-4-one* (**D17**): White solid, yield: 10%. m.p 101.3-102.6°C; ^{1}H NMR (400 MHz, DMSO) δ 8.65 – 8.62 (m, 1H, Pyridine-6-H), 8.13 (td, *J* = 8.1, 1.8 Hz, 1H, Pyridine-4-H), 7.90 (d, *J* = 8.1 Hz, 1H, Pyridine-3-H), 7.67 (d, *J* = 6.9 Hz, 2H, Ph-H), 7.58 (dd, *J* = 7.0, 5.3 Hz, 1H, Pyridine-5-H), 7.51 – 7.44 (m, 3H, Ph-H), 7.36 (s, 2H, Ph-H), 6.84 (d, *J* = 2.1 Hz, 1H, Ph-H), 6.49 (d, *J* = 2.1 Hz, 1H, Ph-H), 3.93 (t, *J* = 6.2 Hz, 2H, -O-$\underline{CH_2}$-CH_2-CH_2-CH_2-N), 3.90 (s, 3H, Ph-OCH_3), 3.84 (d, *J* = 5.8 Hz, 9H, Ph-OCH_3), 3.73 (s, 3H, Ph-OCH_3), 3.61 (d, *J* = 41.8 Hz, 2H, piperazine), 3.24 (d, *J* = 33.5 Hz, 2H, piperazine), 2.45 – 2.20 (m, 3H, piperazine), 2.18 (d, *J* = 6.9 Hz, 2H, -O-CH_2-CH_2-CH_2-$\underline{CH_2}$-N), 1.84 (s, 1H, piperazine), 1.60 (dd, *J* = 13.9, 6.6 Hz, 2H, -O-CH_2-$\underline{CH_2}$-CH_2-CH_2-N), 1.42 (dd, *J* = 15.7, 9.0 Hz, 2H, -O-CH_2-CH_2-$\underline{CH_2}$-CH_2-N); ^{13}CNMR (101 MHz, DMSO) δ 172.68, 164.21, 161.47, 160.74, 158.63, 153.12, 150.88, 149.14, 148.89, 140.41, 140.11, 139.80, 131.51, 129.64, 129.42, 127.00, 126.11, 126.02, 119.19, 115.50, 108.91, 106.23, 96.38, 93.56, 72.02, 60.64, 57.59, 56.53, 53.13, 52.56, 46.85, 41.86, 27.95, 23.17; ESI-HRMS calcd for $C_{43}H_{45}O_9N_5Cl$ $[M+H]^+$810.29003, found 810.28766.

Data for *3-(4-(4-(5-chloro-1-(4-methoxyphenyl)-3-methyl-1H-pyrazole-4-carbonyl)piperazin-1-yl)butoxy)-5,7-dimethoxy-2-(3,4,5-trimethoxyphenyl)-4H-chromen-4-one* (**D18**): White solid, yield: 24%. m.p 80.0-81.8°C; ^{1}H NMR (400 MHz, DMSO) δ 7.48 (d, *J* = 8.9 Hz, 2H, Ph-H), 7.38 (s, 2H, Ph-H), 7.09 (d, *J* = 9.0 Hz, 2H, Ph-H), 6.84 (d, *J* = 2.1 Hz, 1H, Ph-H), 6.49 (d, *J* = 2.1 Hz, 1H, Ph-H), 3.96 (t, *J* = 6.2 Hz, 2H, -O-$\underline{CH_2}$-CH_2-CH_2-CH_2-N), 3.90 (s, 3H, Ph-OCH_3), 3.86 (d, *J* = 11.0 Hz, 9H, Ph-OCH_3), 3.83 (s, 3H, Ph-OCH_3), 3.74 (s, 3H, Ph-OCH_3), 3.49 (d, *J* = 55.9 Hz, 4H, piperazine), 2.36 (s, 3H, piperazine), 2.27 (s, 2H, -O-CH_2-CH_2-CH_2-$\underline{CH_2}$-N), 2.23 (s, 1H, piperazine), 2.20 (s, 3H, pyrazol-CH_3), 1.64 (dd, *J* = 13.8, 6.4 Hz, 2H, -O-CH_2-$\underline{CH_2}$-CH_2-CH_2-N), 1.50 (d, *J* = 6.8 Hz, 2H, -O-CH_2-CH_2-$\underline{CH_2}$-CH_2-N)); ^{13}C NMR (101 MHz, DMSO) δ 172.69, 164.21, 161.80, 160.74, 159.73, 158.64, 153.13, 152.01, 147.72, 140.42, 139.81, 130.72, 127.31, 126.13, 125.08, 114.77, 114.48, 108.92, 106.24, 96.39, 93.56, 72.05, 60.65, 57.68, 56.54, 55.99, 53.77, 52.91, 47.18, 41.94, 28.00, 23.25, 13.13; ESI-HRMS calcd for $C_{40}H_{46}O_{10}N_4Cl$ $[M+H]^+$777.28970, found 777.28772.

Data for *3-(4-(4-(5-chloro-1,3-dimethyl-1H-pyrazole-4-carbonyl)piperazin-1-yl)butoxy)-5,7-dimethoxy-2-(3,4,5-trimethoxyphenyl)-4H-chromen-4-one* (**D19**): White solid, yield: 28%. m.p 75.4-74.5°C; ^{1}H NMR (400 MHz, DMSO) δ 7.38 (s, 2H, Ph-H), 6.84 (s, 1H, Ph-H), 6.49 (s, 1H, Ph-H), 3.95 (t, *J* = 6.3 Hz, 2H, -O-$\underline{CH_2}$-CH_2-CH_2-CH_2-N), 3.90 (s, 3H, Ph-OCH_3), 3.86 (d, *J* = 11.3 Hz, 9H, Ph-OCH_3), 3.74 (s, 3H, -CH_3), 3.74 (s, 3H, Ph-OCH_3), 3.54 (d, *J* = 16.9 Hz, 2H, piperazine), 3.28 (s, 2H, piperazine), 2.40 (s, 2H, piperazine), 2.26 (s, 2H, -O-CH_2-CH_2-CH_2-$\underline{CH_2}$-N), 2.17 (s, 2H, piperazine), 2.11 (s, 3H, pyrazol-CH_3), 1.70 – 1.58 (m, 2H, -O-CH_2-$\underline{CH_2}$-CH_2-CH_2-N), 1.50 (d, *J* = 6.4 Hz, 2H, -O-CH_2-CH_2-$\underline{CH_2}$-CH_2-N); ^{13}C NMR (101 MHz, DMSO) δ 172.69, 164.21, 162.13, 160.74, 158.63, 153.13, 152.01, 146.23, 140.42, 139.81, 126.12, 125.10, 112.94, 108.91, 106.22, 96.38,

95.35, 93.56, 72.01, 60.64, 57.64, 56.54, 53.78, 52.97, 47.08, 41.98, 36.39, 27.97, 23.18, 13.05; ESI-HRMS calcd for $C_{34}H_{42}O_9N_4Cl$ $[M+H]^+$685.26348, found 685.26392.

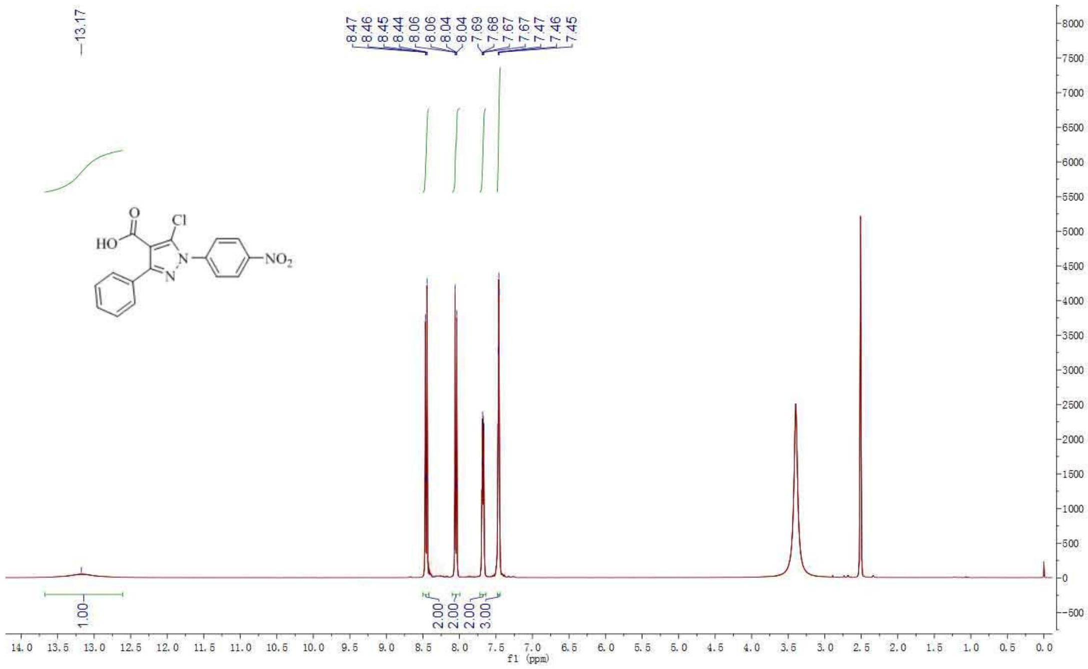

Figure S1 ^{1}H NMR spectrum of title compound **b-4**

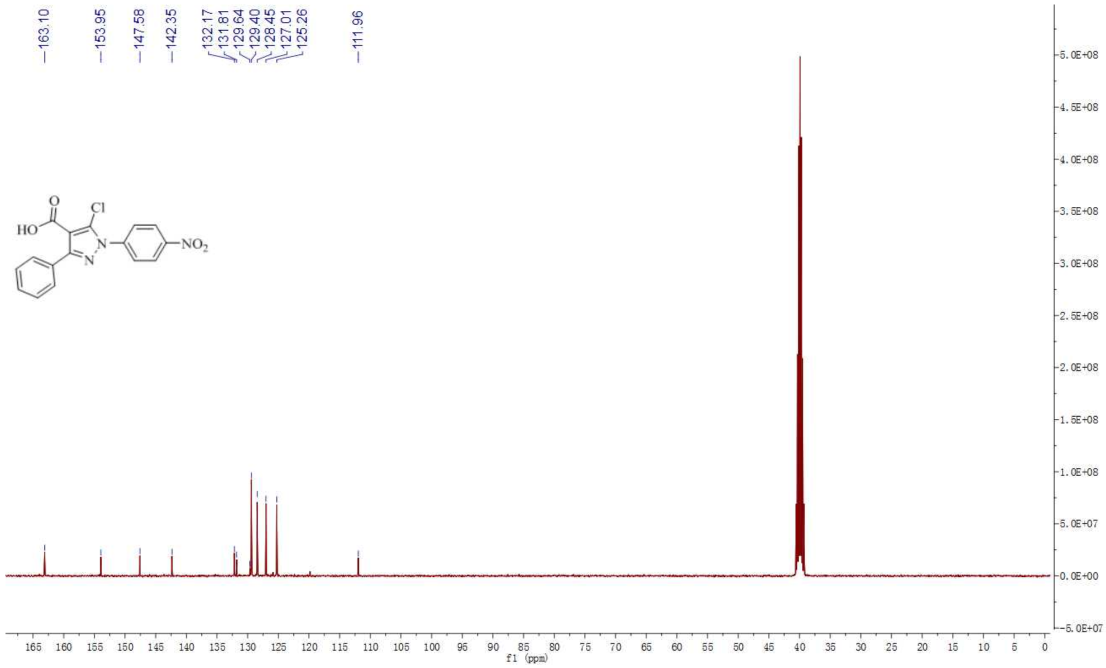

Figure S2 ^{13}C NMR spectrum of title compound **b-4**

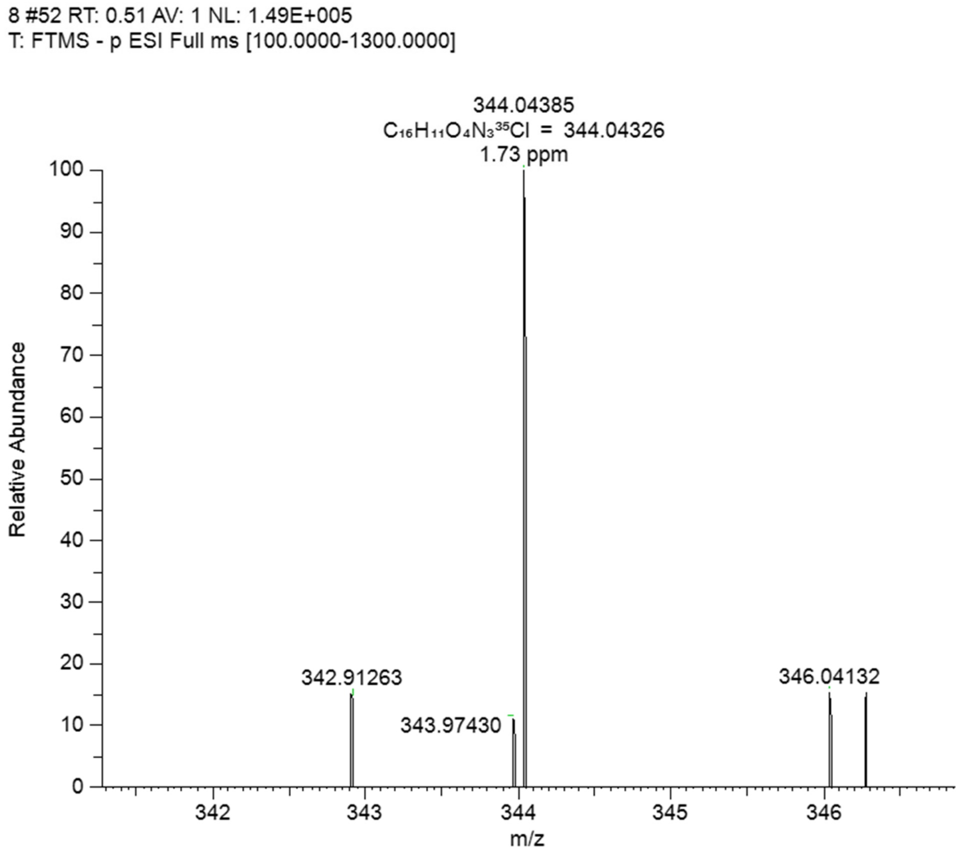

Figure S3 HRMS spectrum of title compound **b-4**

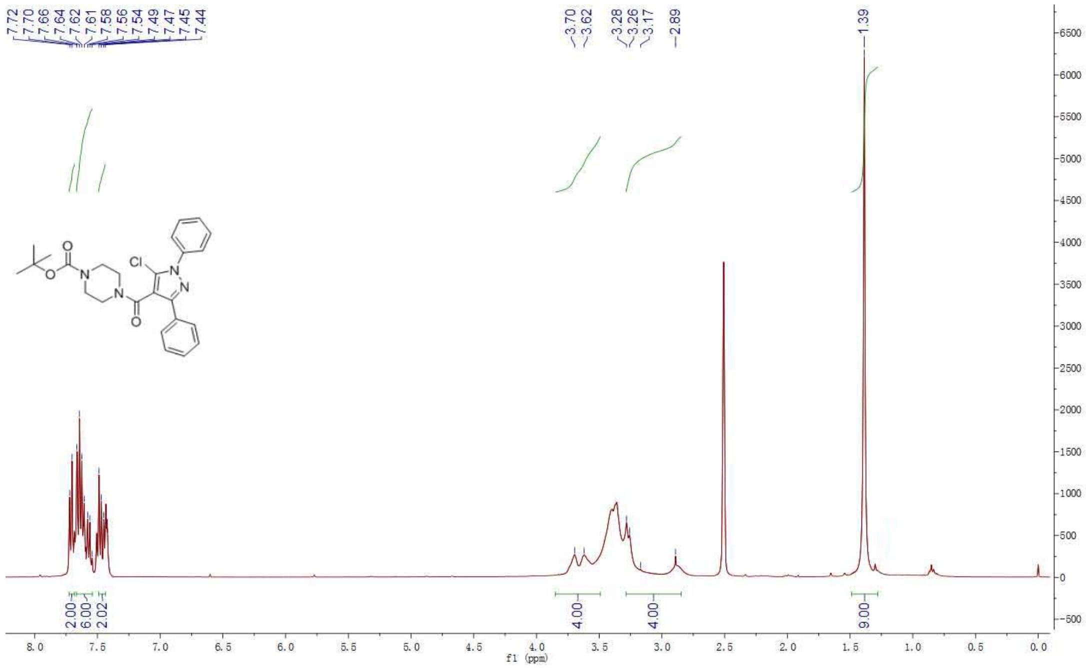

Figure S4 ^{1}H NMR spectrum of title compound **c-2**

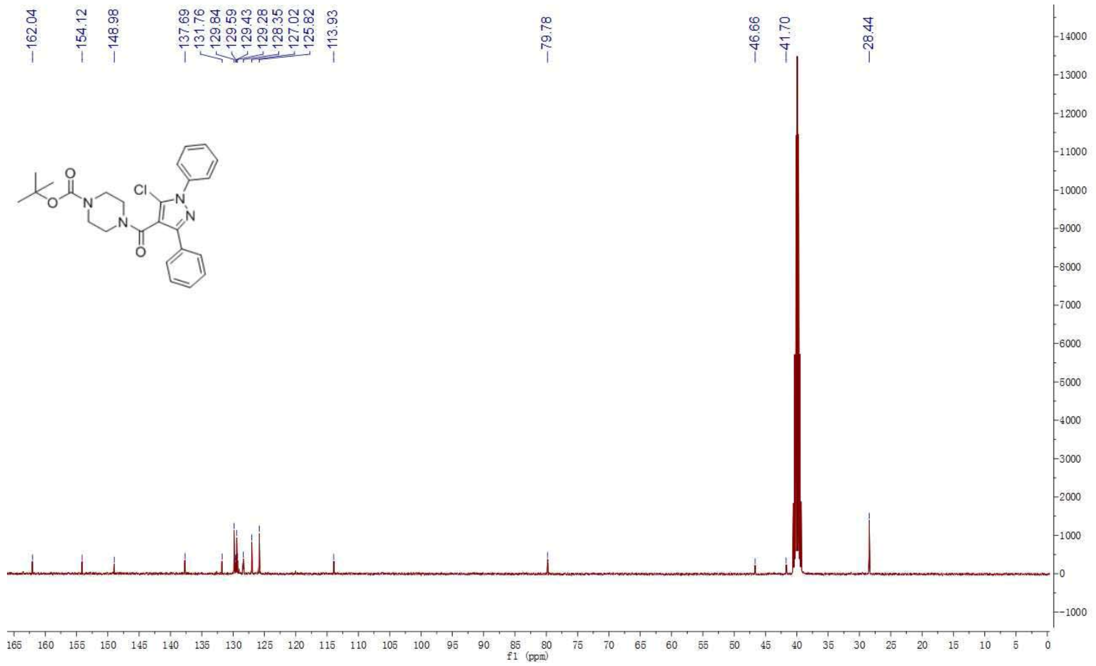

Figure S5 ^{13}C NMR spectrum of title compound **c-2**

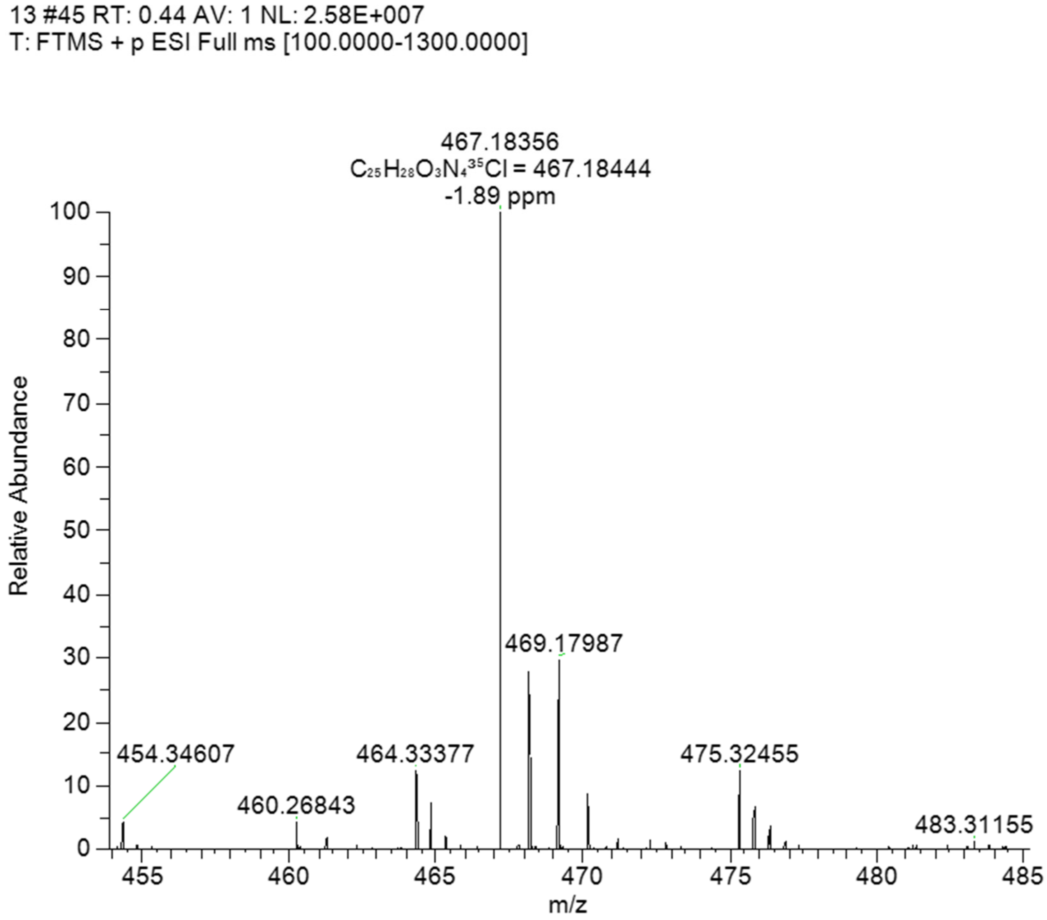

Figure S6 HRMS spectrum of title compound **c-2**

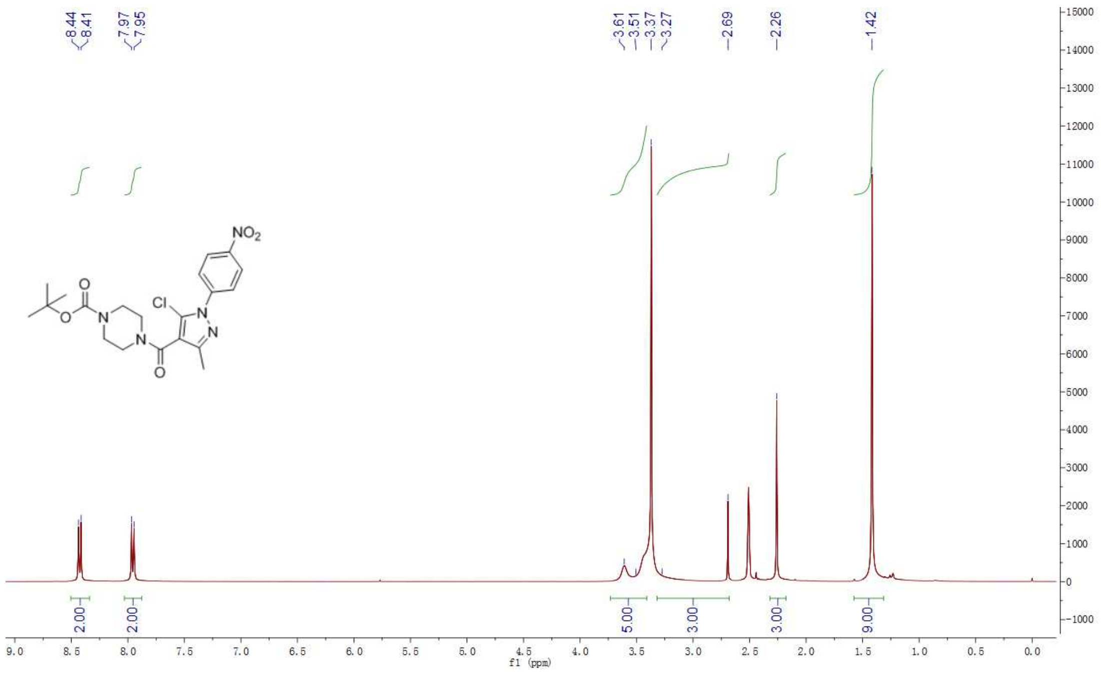

Figure S7 ^{1}H NMR spectrum of title compound **c-3**

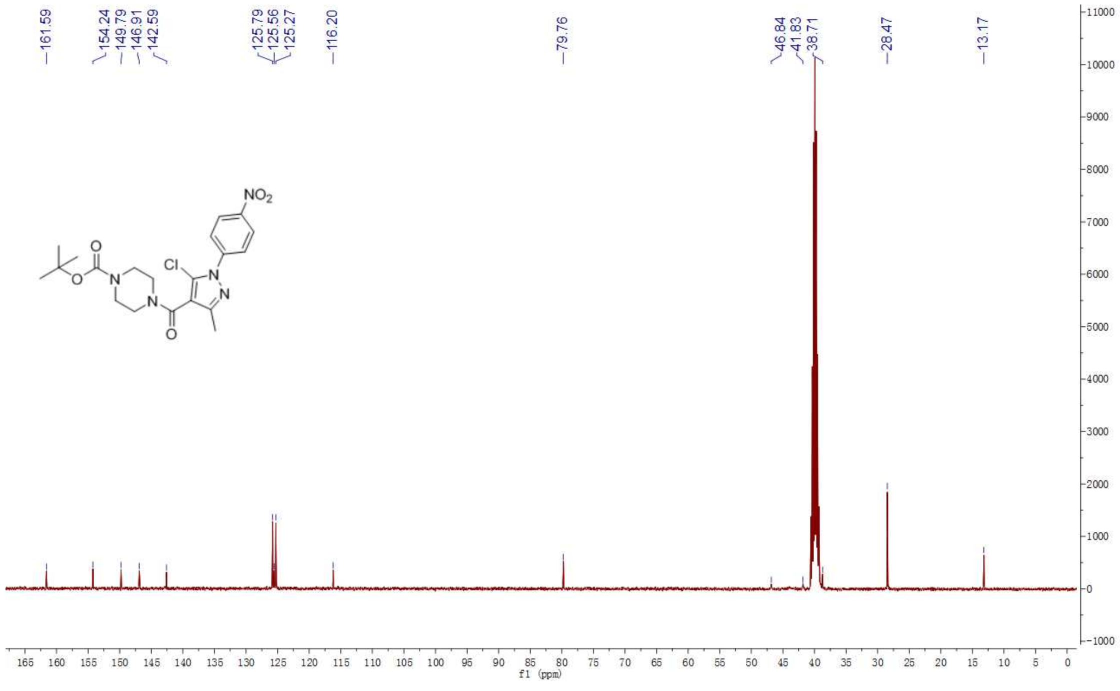

Figure S8 ^{13}C NMR spectrum of title compound **c-3**

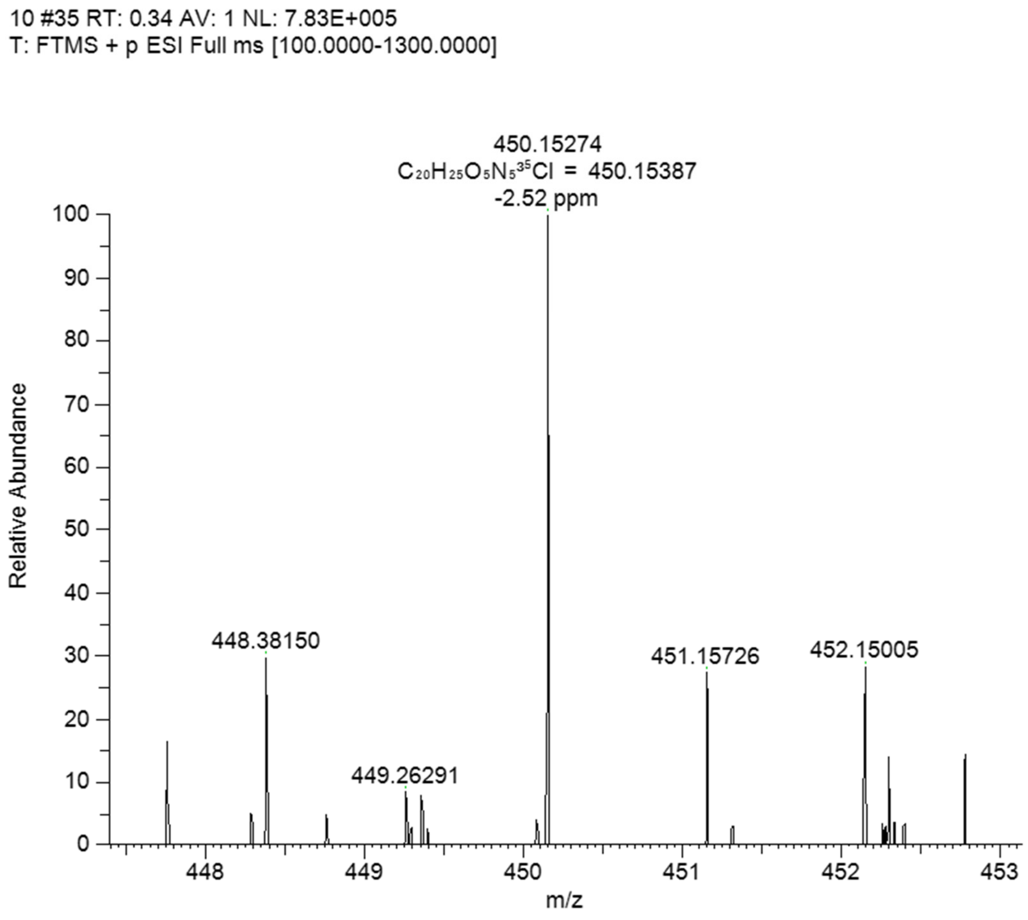

Figure S9 HRMS spectrum of title compound **c-3**

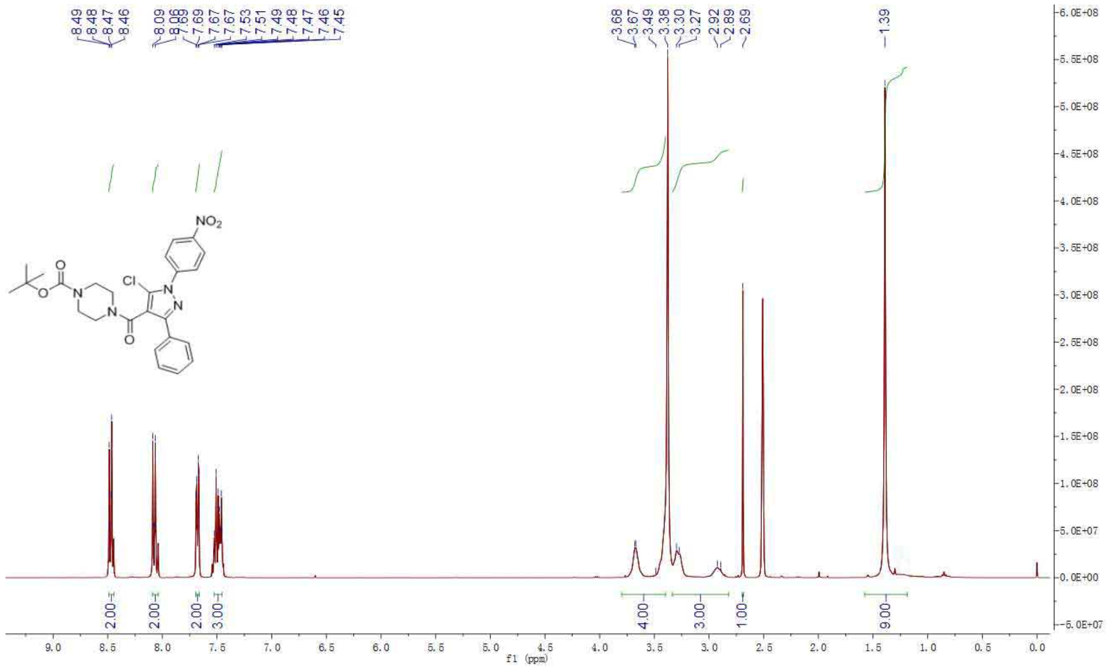

Figure S10 ^{1}H NMR spectrum of title compound **c-4**

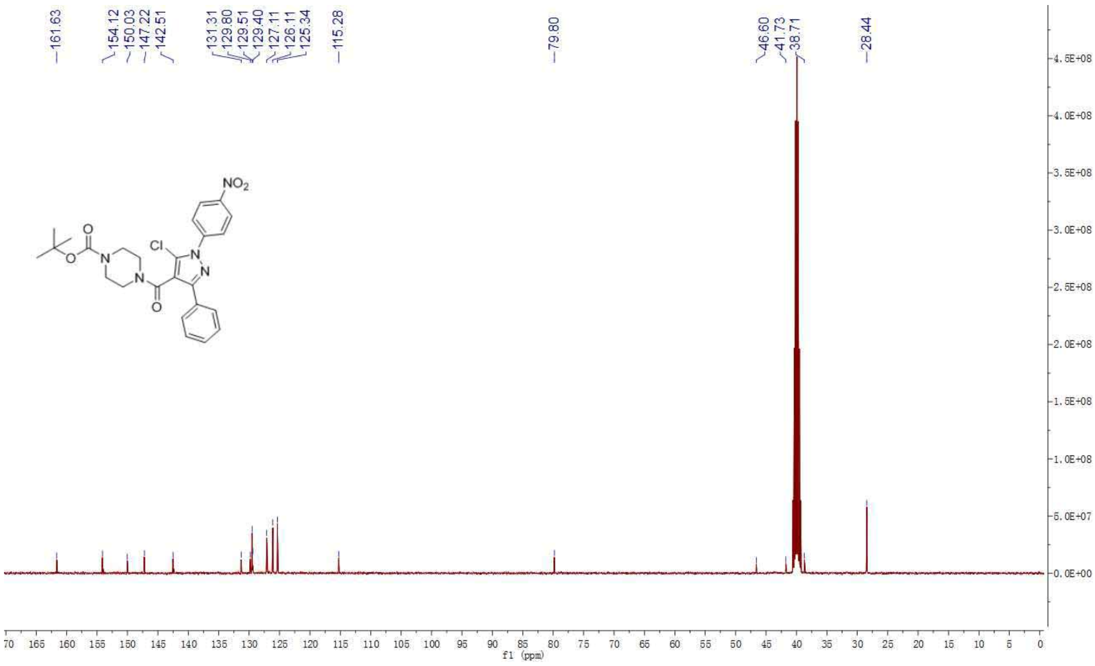

Figure S11 ^{13}C NMR spectrum of title compound **c-4**

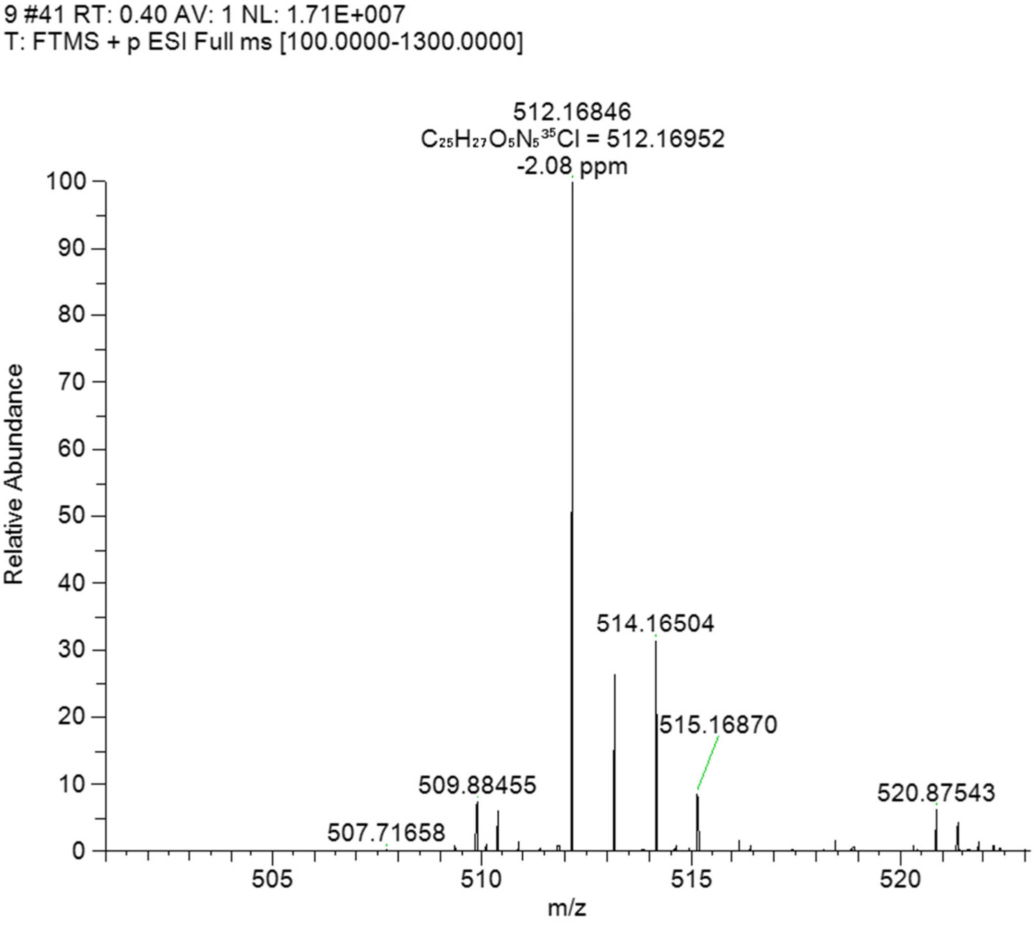

Figure S12 HRMS spectrum of title compound **c-4**

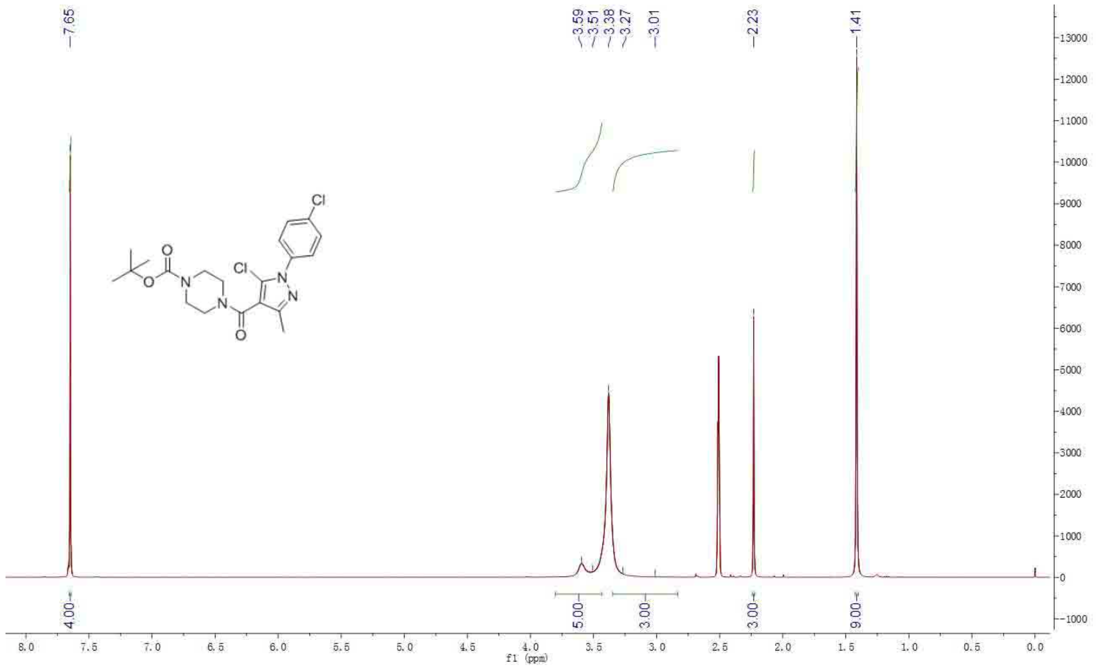

Figure S13 ^{1}H NMR spectrum of title compound **c-5**

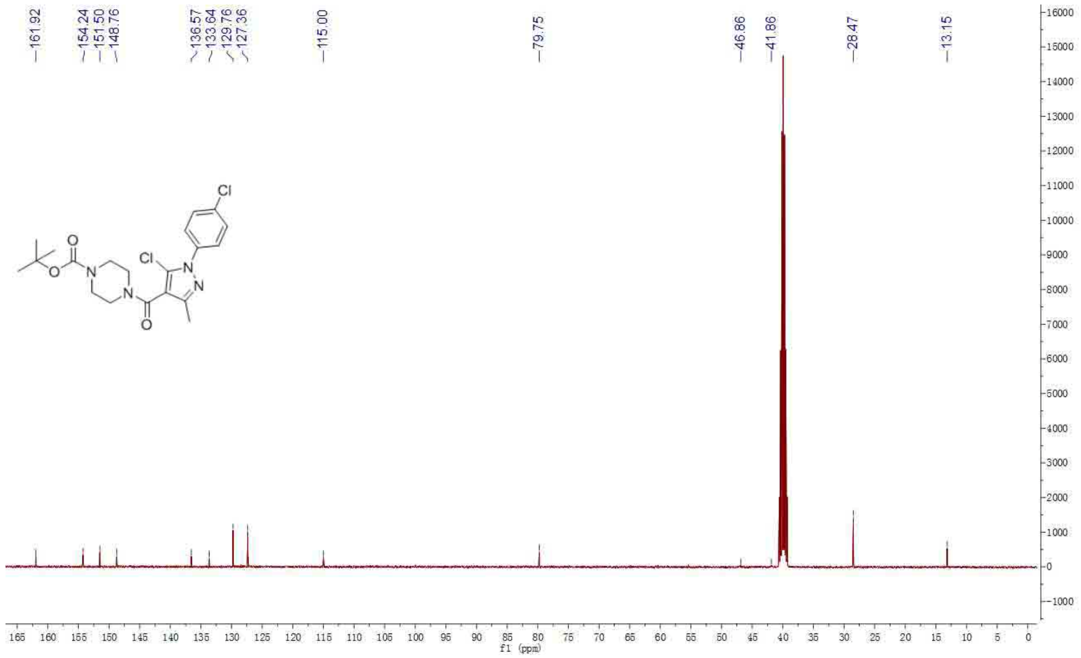

Figure S14 ^{13}C NMR spectrum of title compound **c-5**

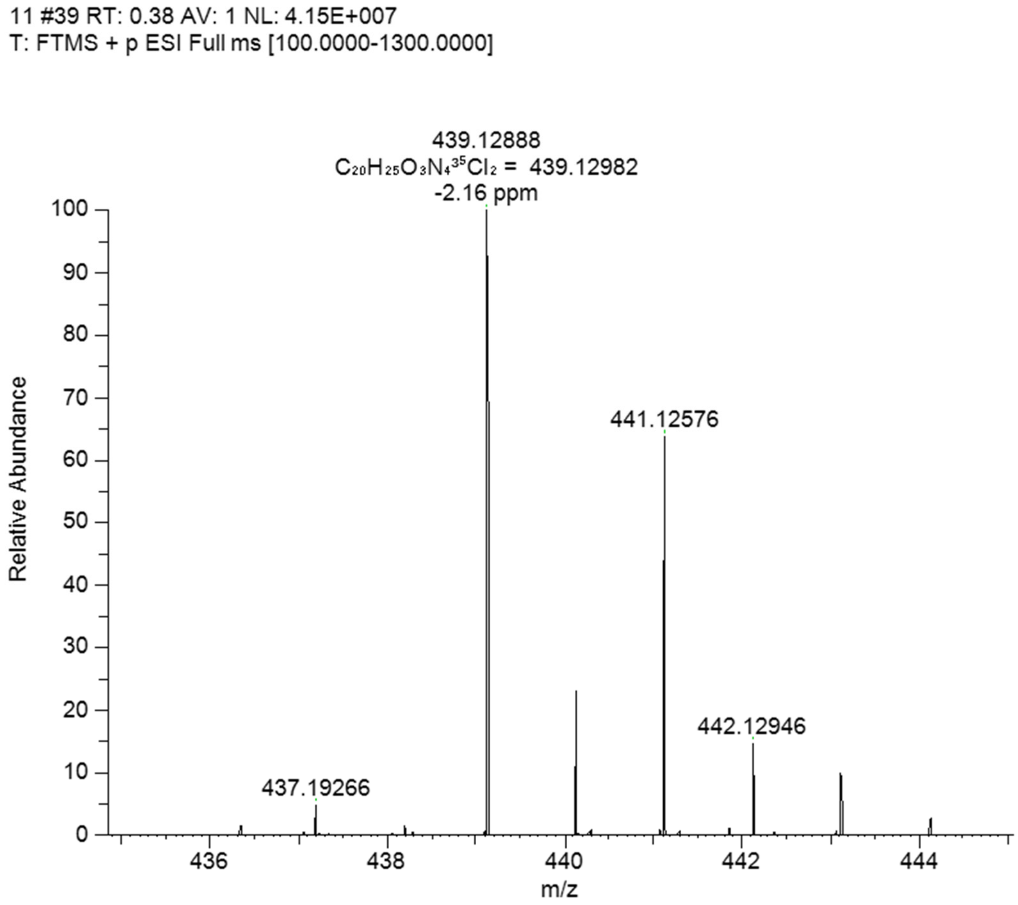

Figure S15 HRMS spectrum of title compound **c-5**

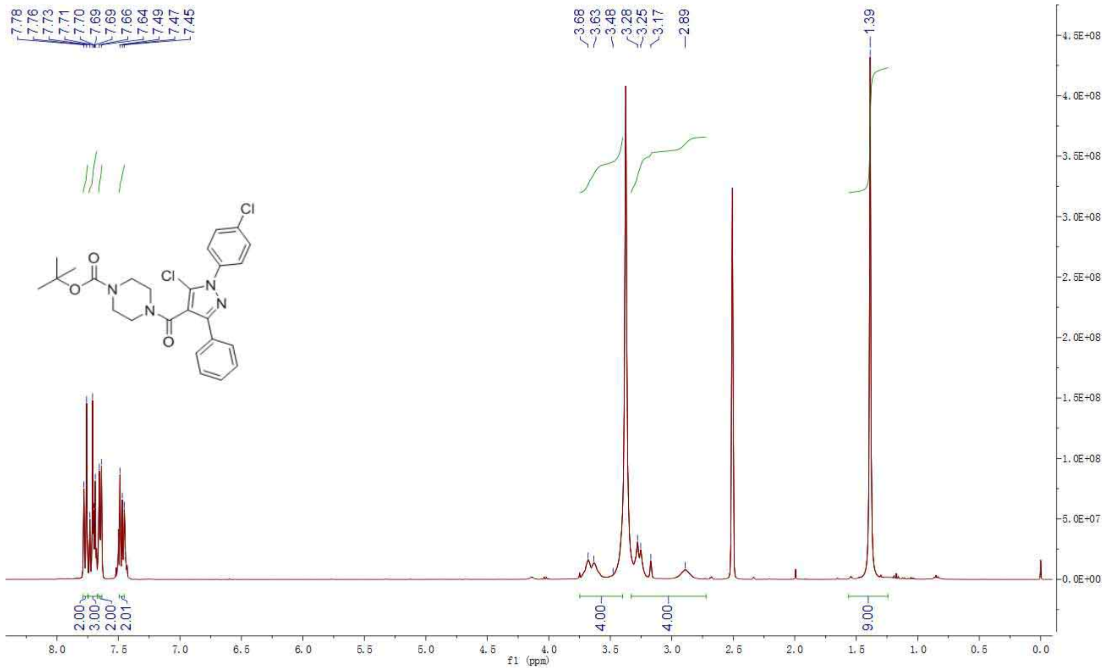

Figure S16 ^{1}H NMR spectrum of title compound **c-6**

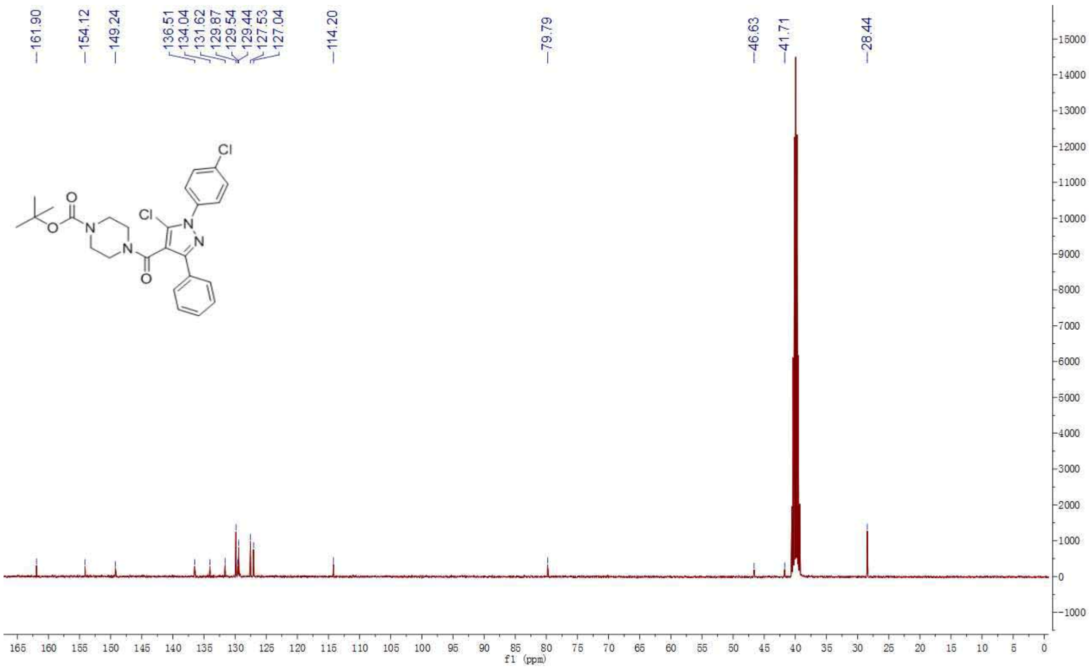

Figure S17 ^{13}C NMR spectrum of title compound **c-6**

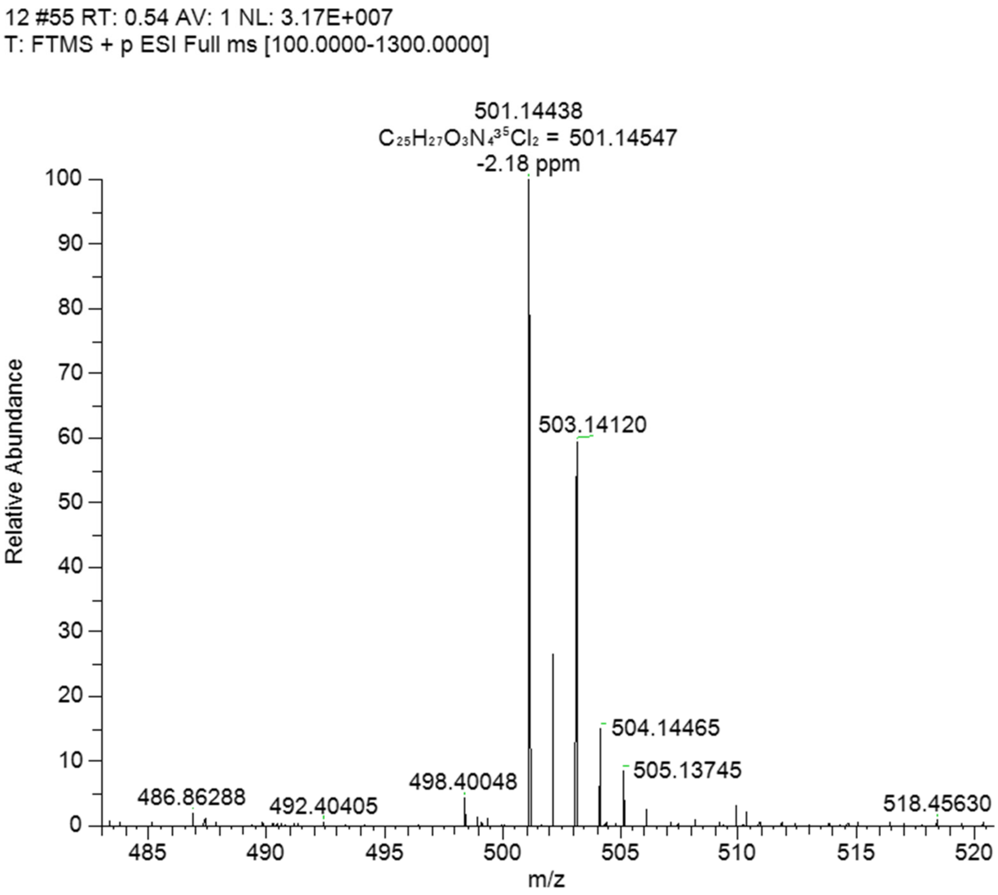

Figure S18 HRMS spectrum of title compound **c-6**

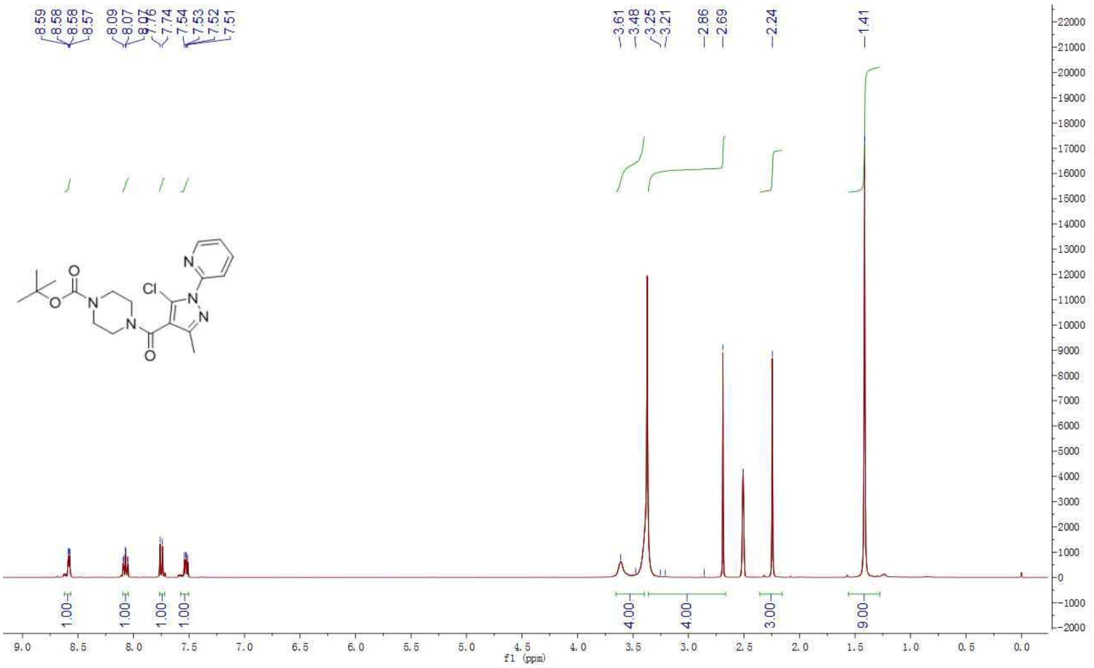

Figure S19 ^{1}H NMR spectrum of title compound **c-7**

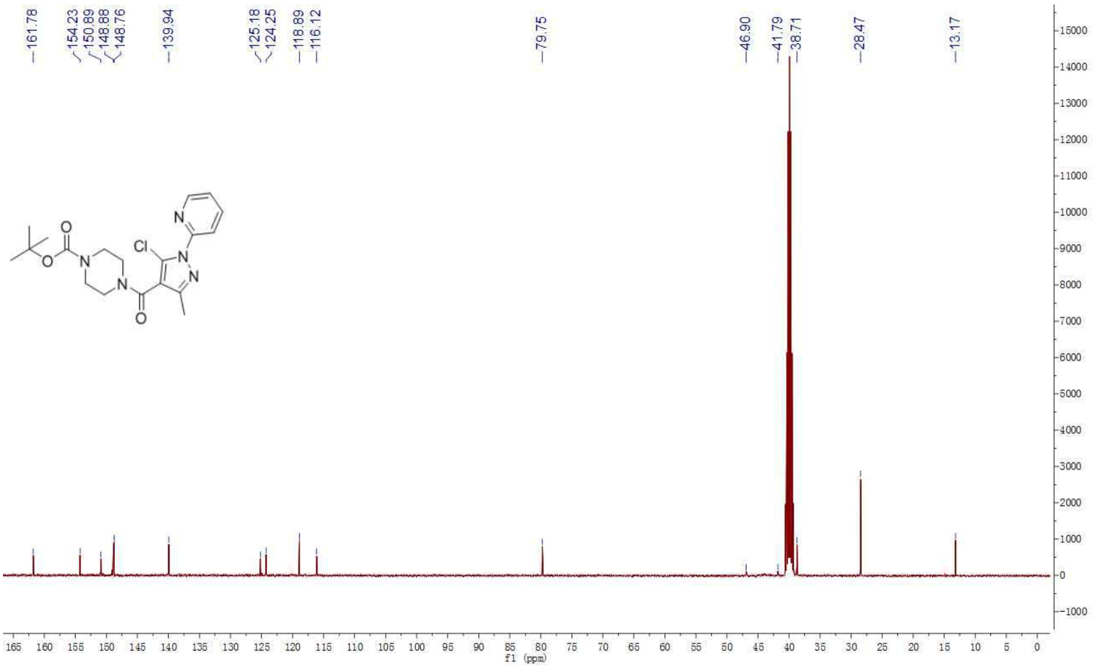

Figure S20 ^{13}C NMR spectrum of title compound **c-7**

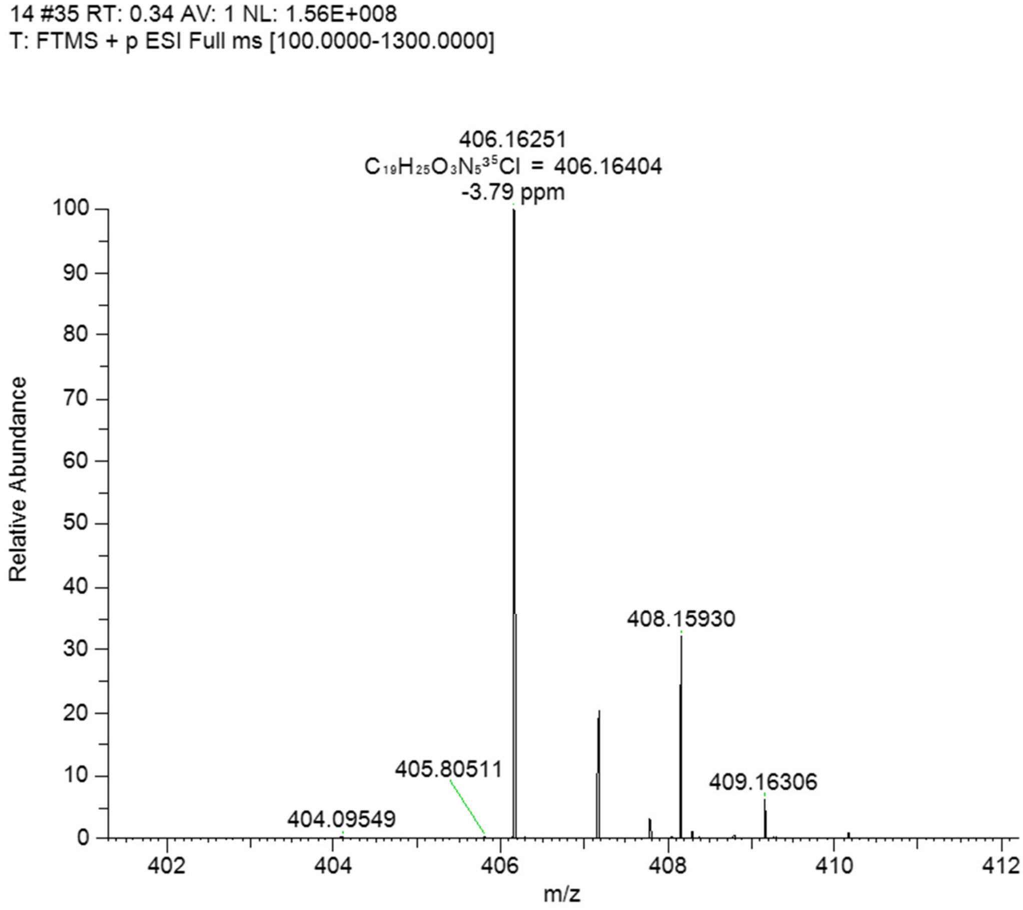

Figure S21 HRMS spectrum of title compound **c-7**

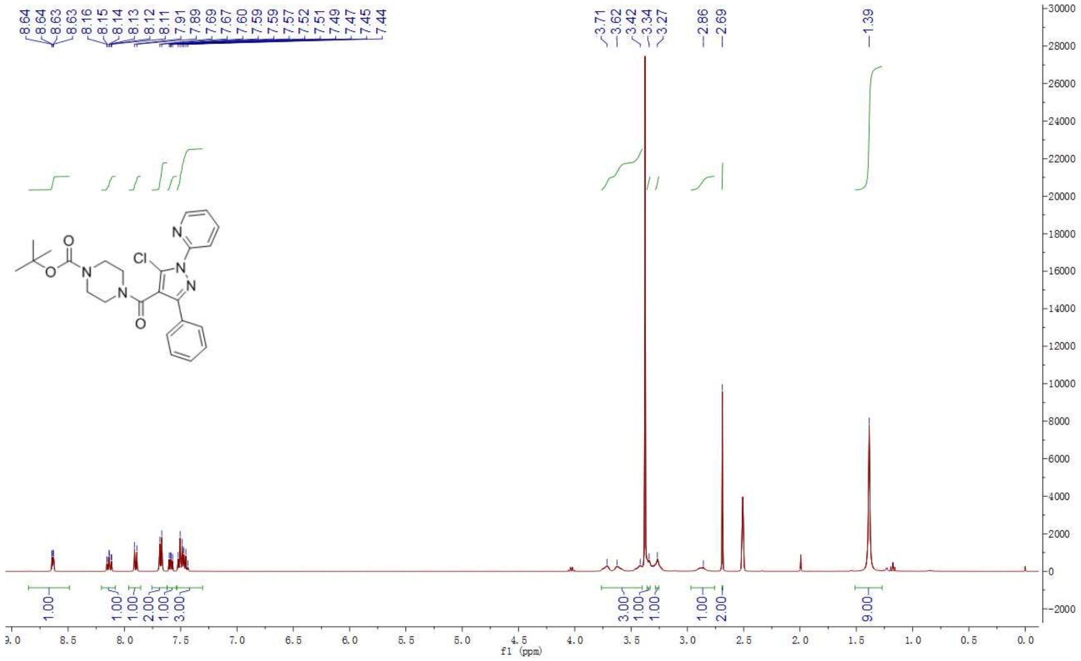

Figure S22 ^{1}H NMR spectrum of title compound **c-8**

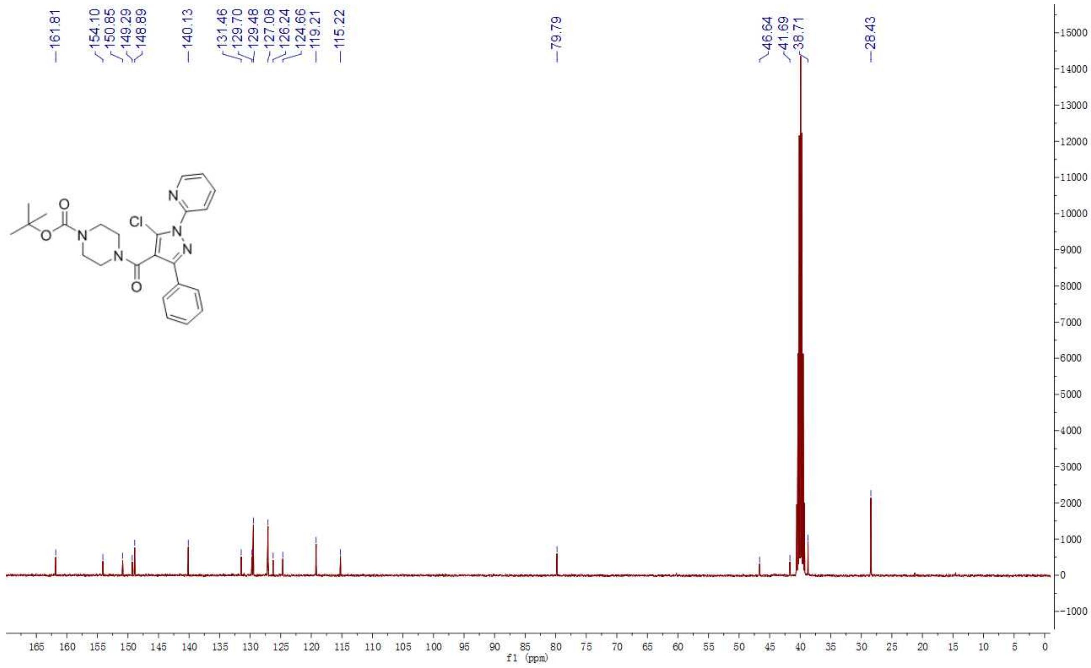

Figure S23 ^{13}C NMR spectrum of title compound **c-8**

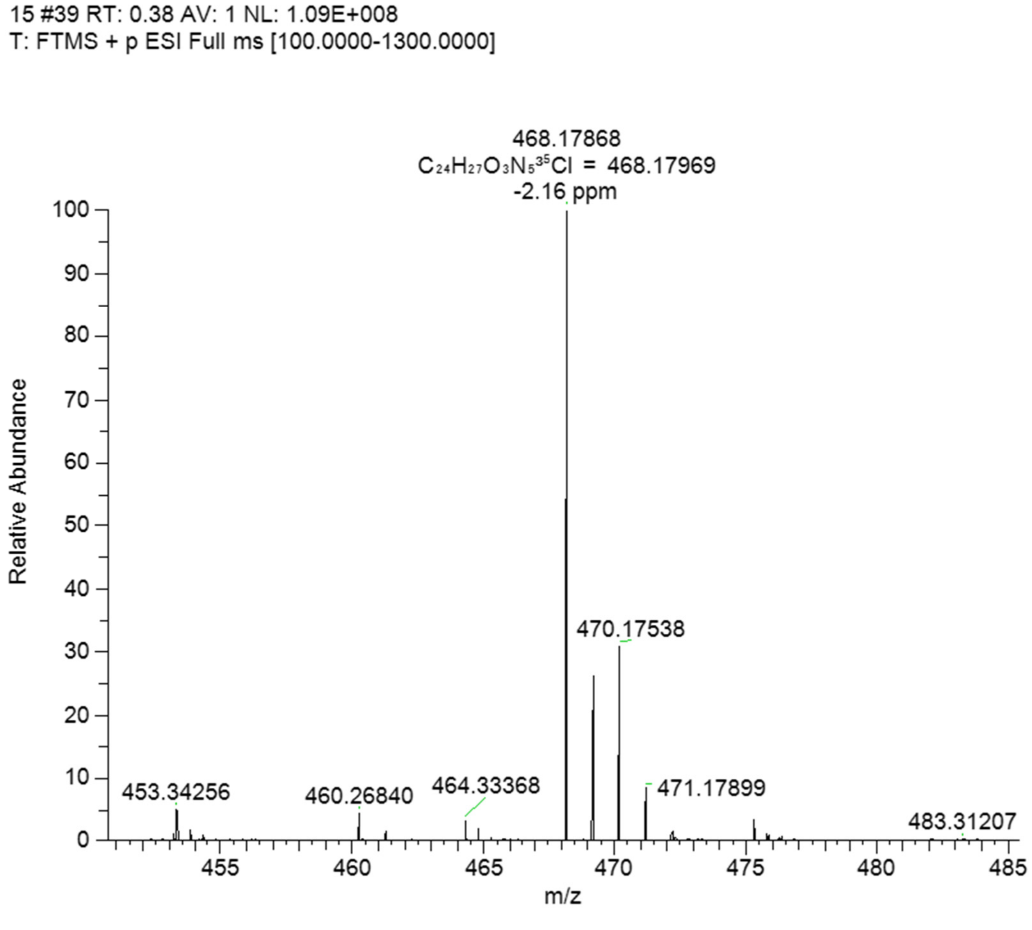

Figure S24 HRMS spectrum of title compound **c-8**

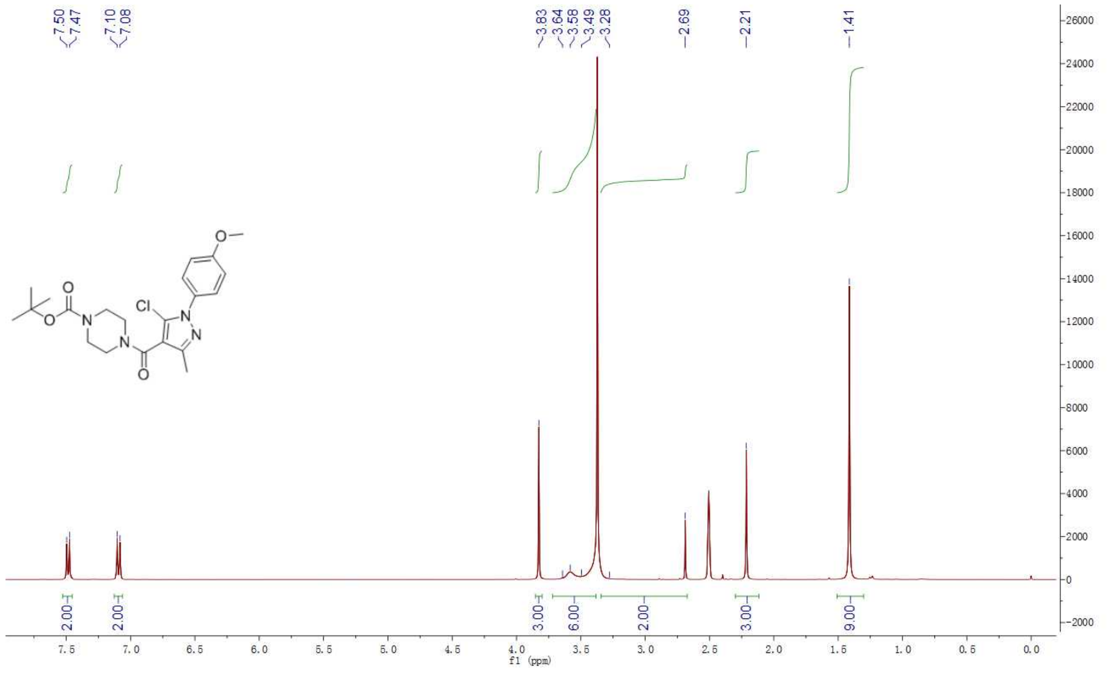

Figure S25 ^{1}H NMR spectrum of title compound **c-9**

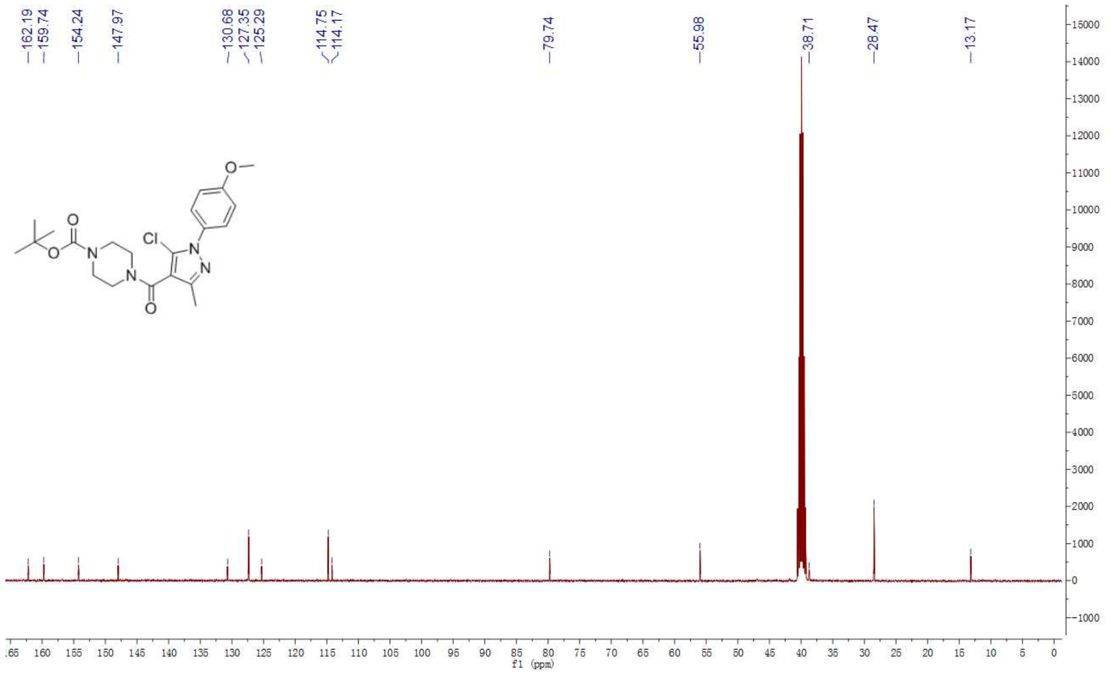

Figure S26 ^{13}C NMR spectrum of title compound **c-9**

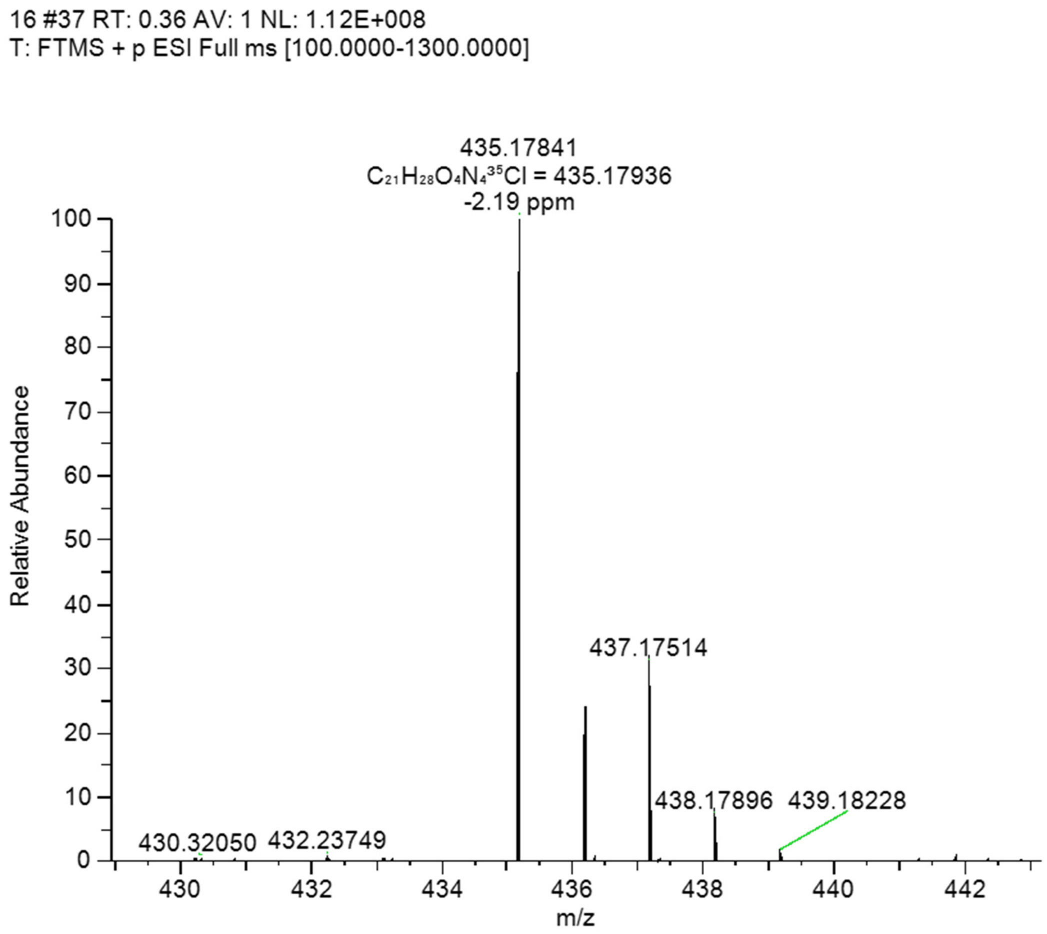

Figure S27 HRMS spectrum of title compound **c-9**

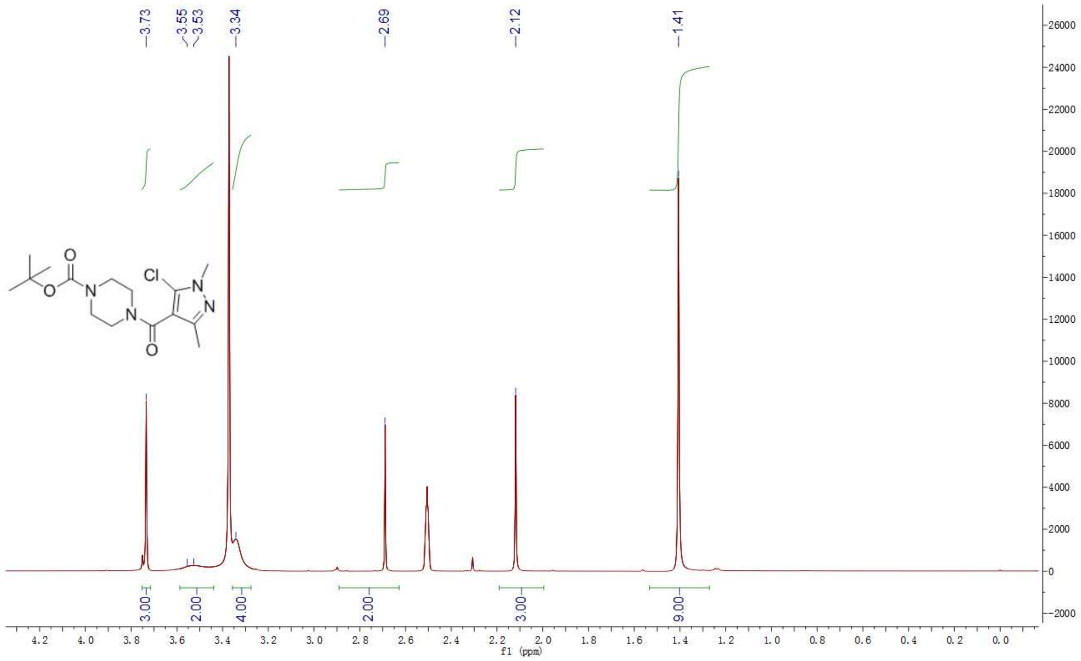

Figure S28 ^{1}H NMR spectrum of title compound **c-10**

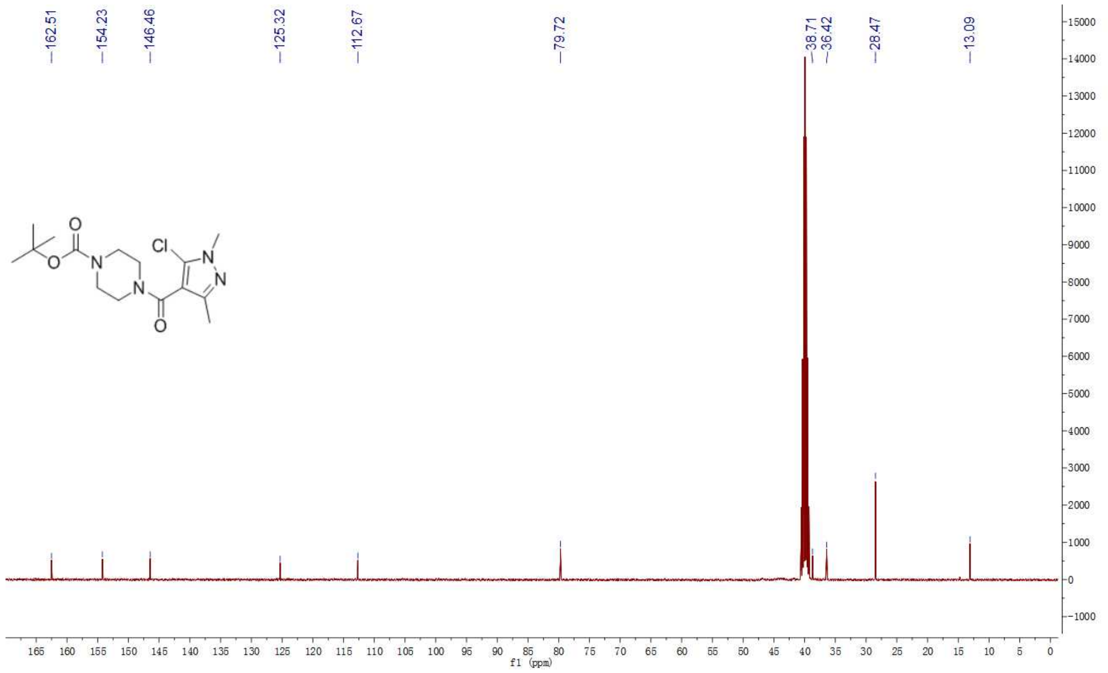

Figure S29 ^{13}C NMR spectrum of title compound **c-10**

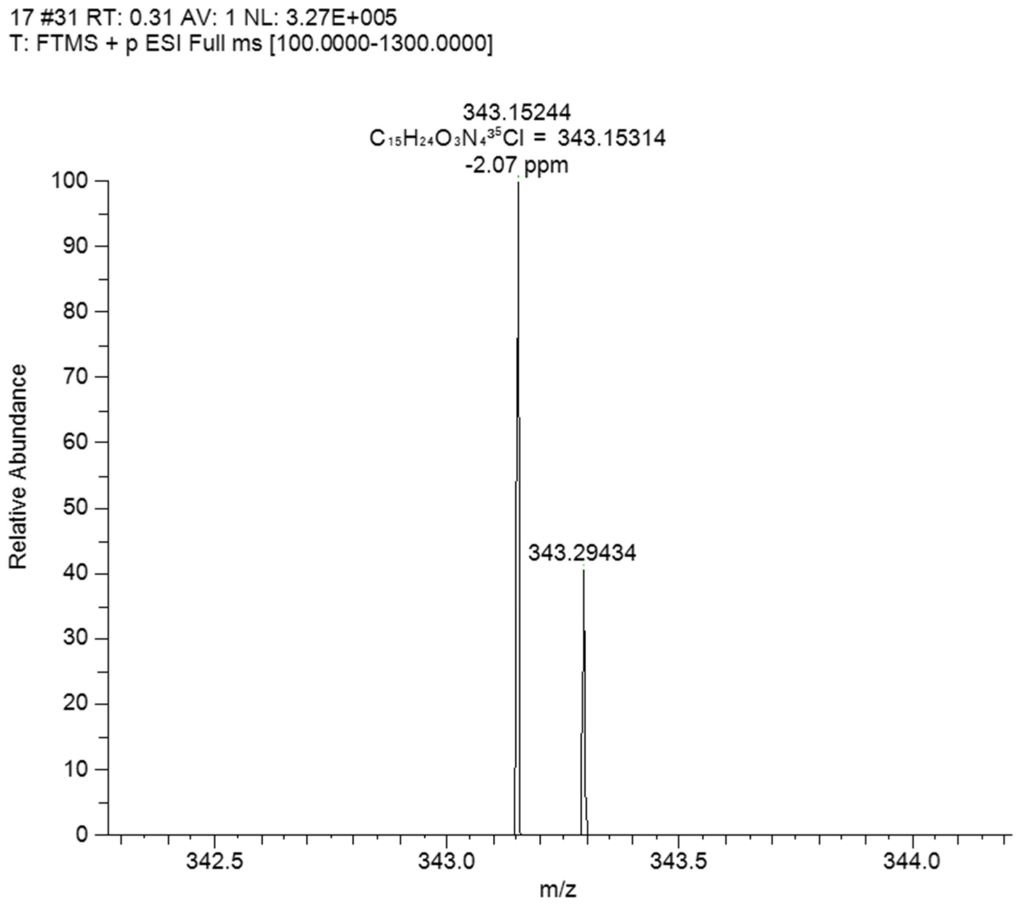

Figure S30 HRMS spectrum of title compound **c-10**

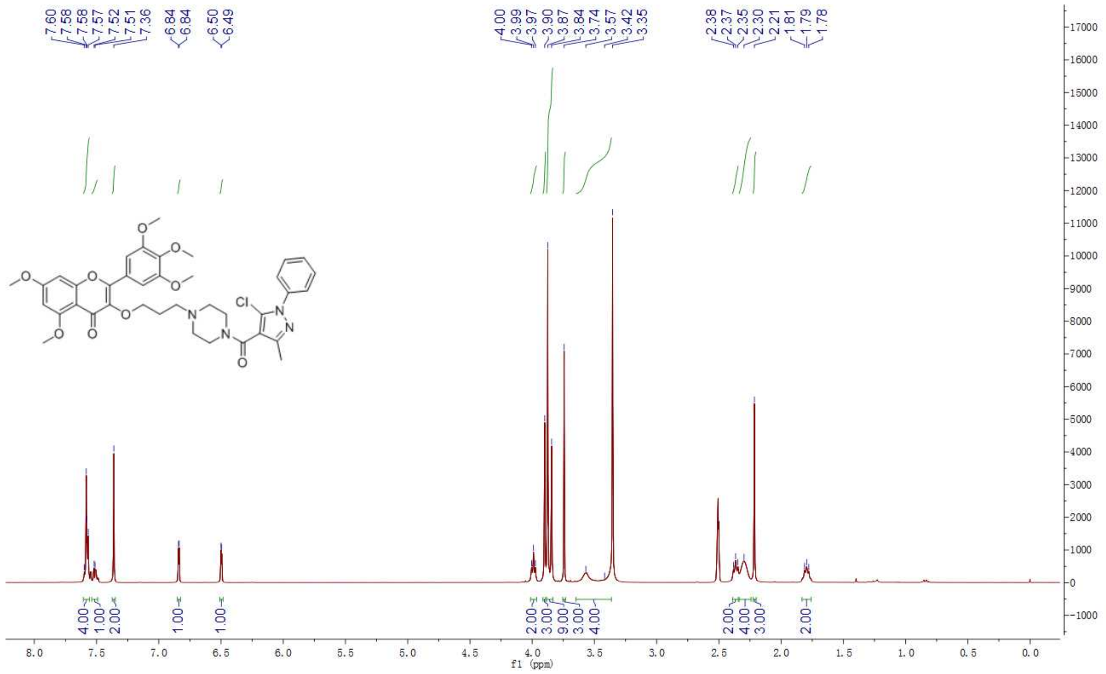

Figure S31 ^{1}H NMR spectrum of title compound **D1**

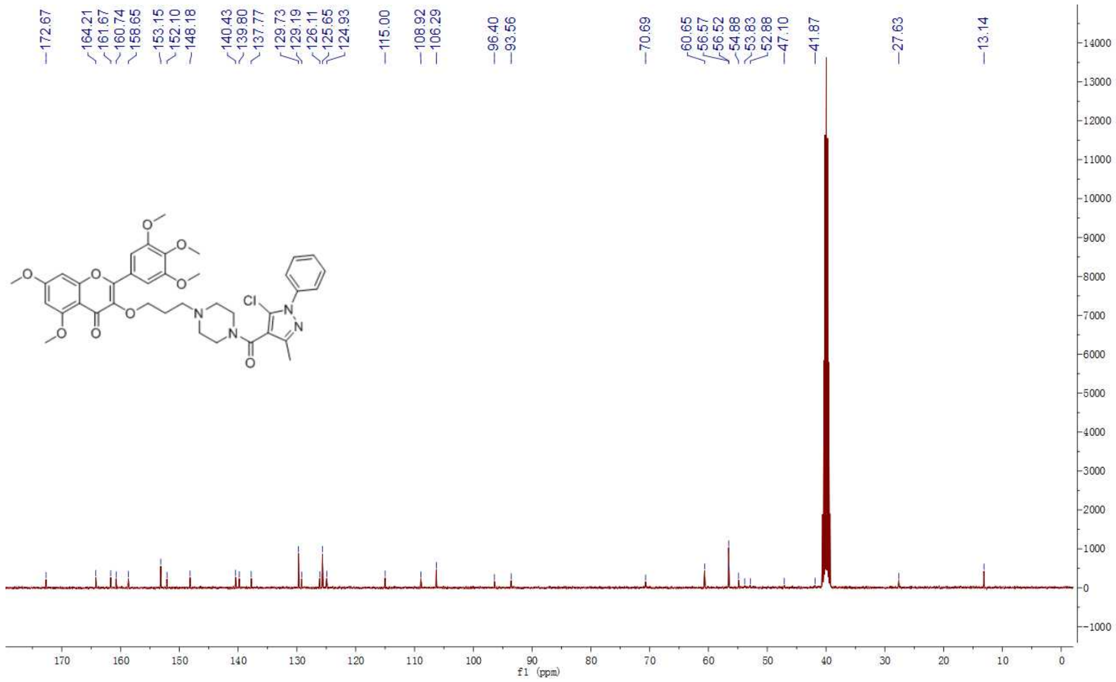

Figure S32 ^{13}C NMR spectrum of title compound **D1**

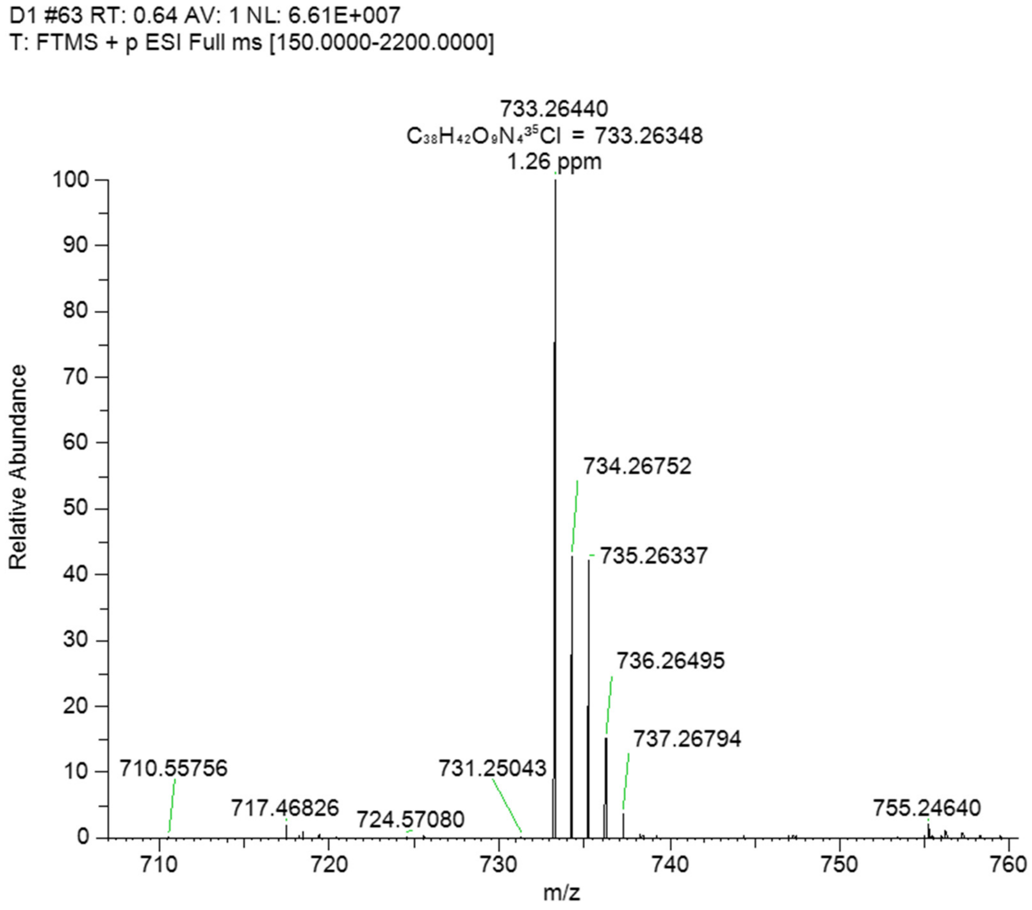

Figure S33 HRMS spectrum of title compound **D1**

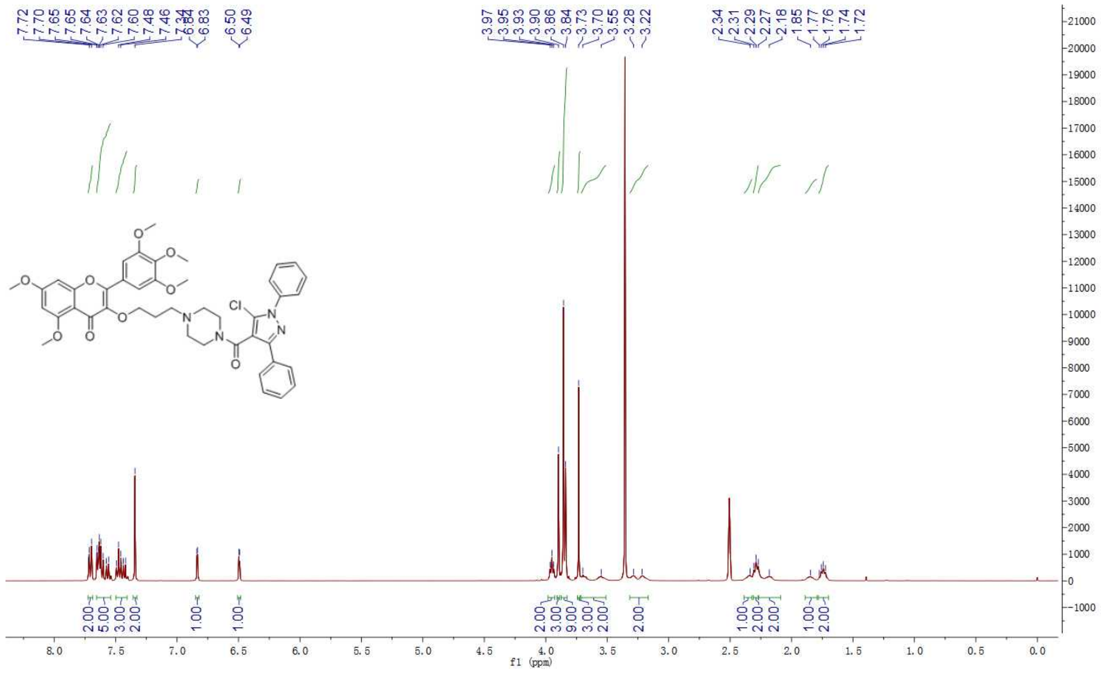

Figure S34 ^{1}H NMR spectrum of title compound **D2**

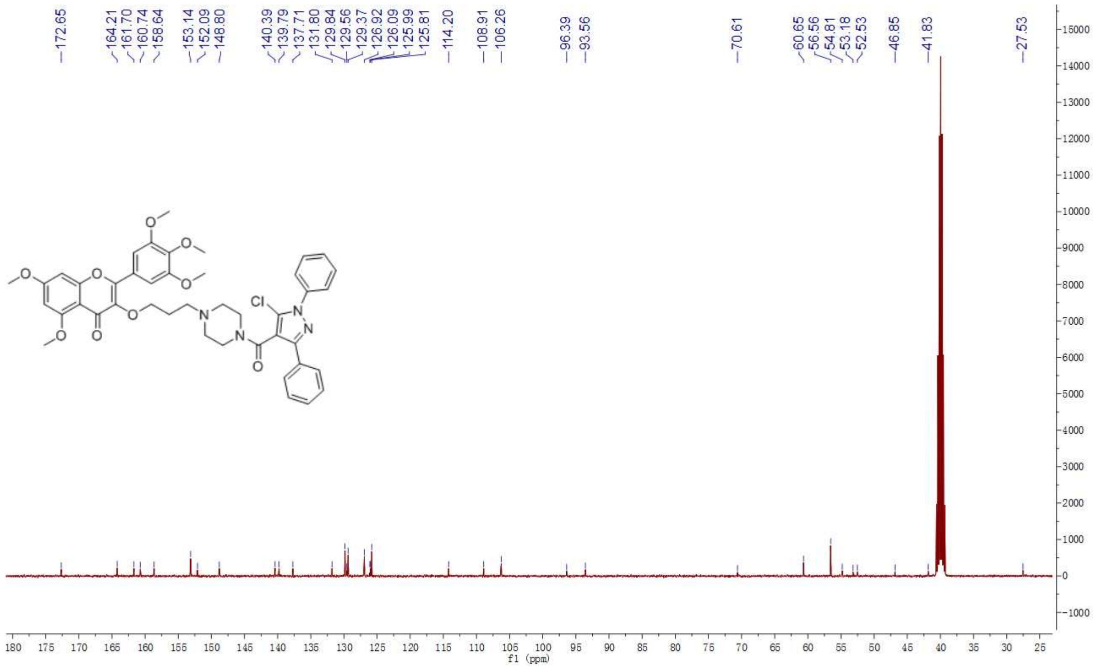

Figure S35 ^{13}C NMR spectrum of title compound **D2**

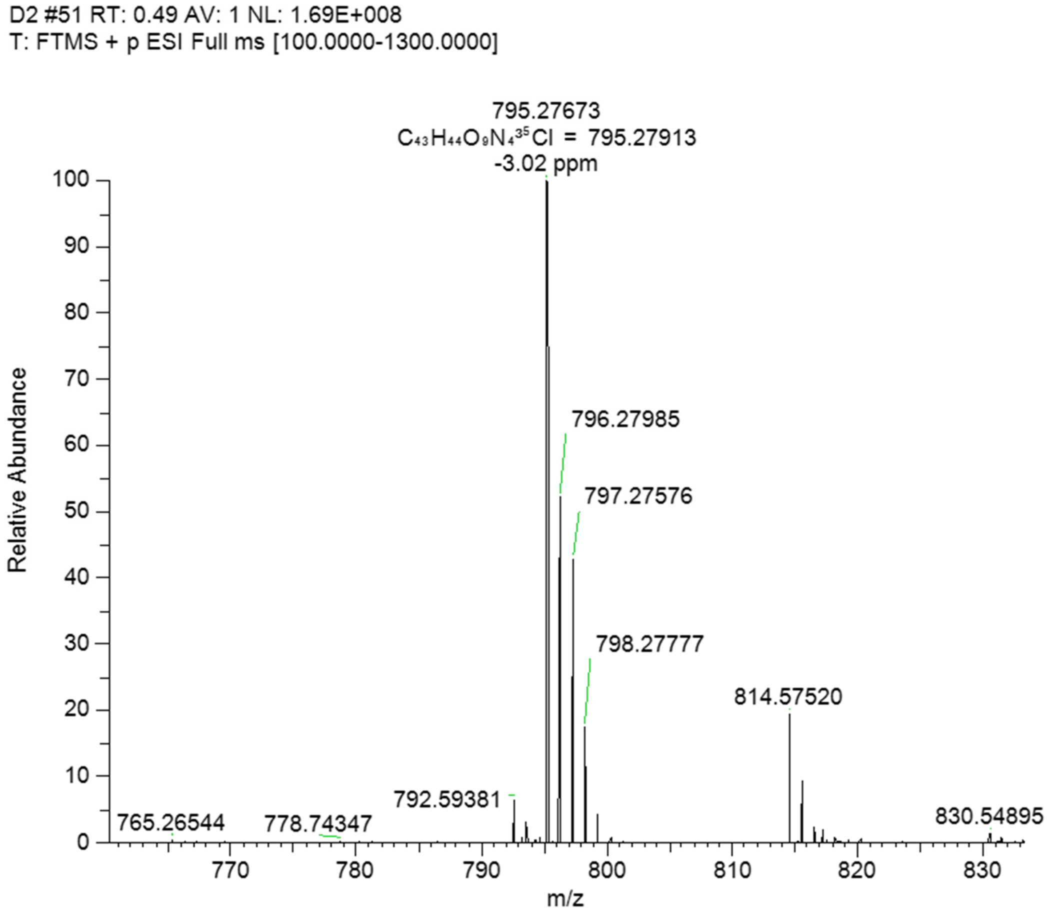

Figure S36 HRMS spectrum of title compound **D2**

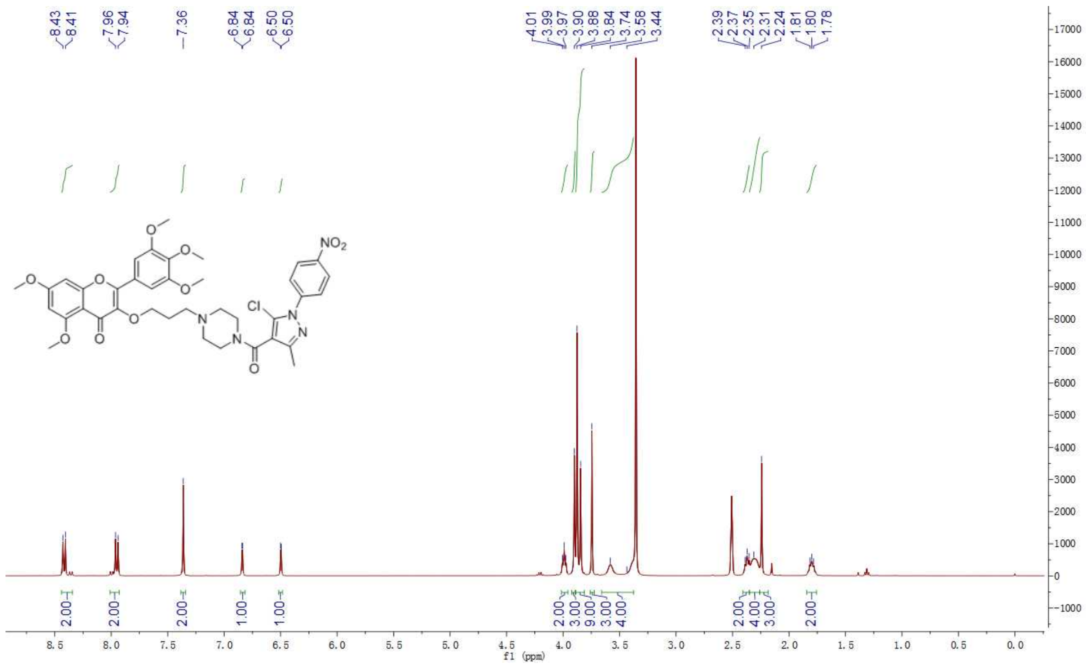

Figure S37 ^{1}H NMR spectrum of title compound **D3**

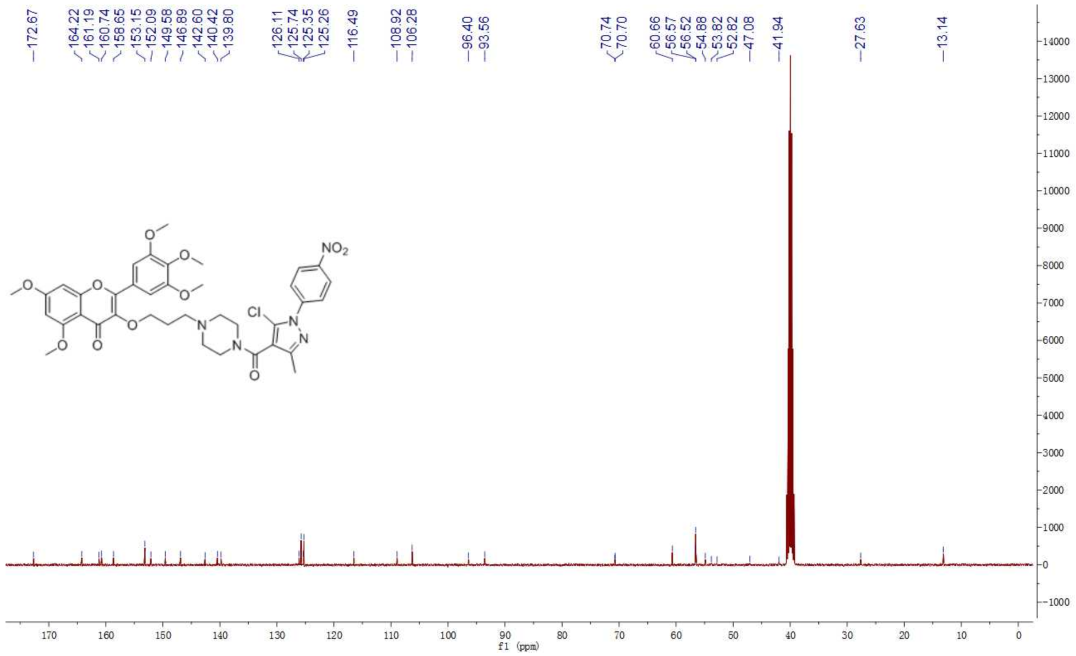

Figure S38 ^{13}C NMR spectrum of title compound **D3**

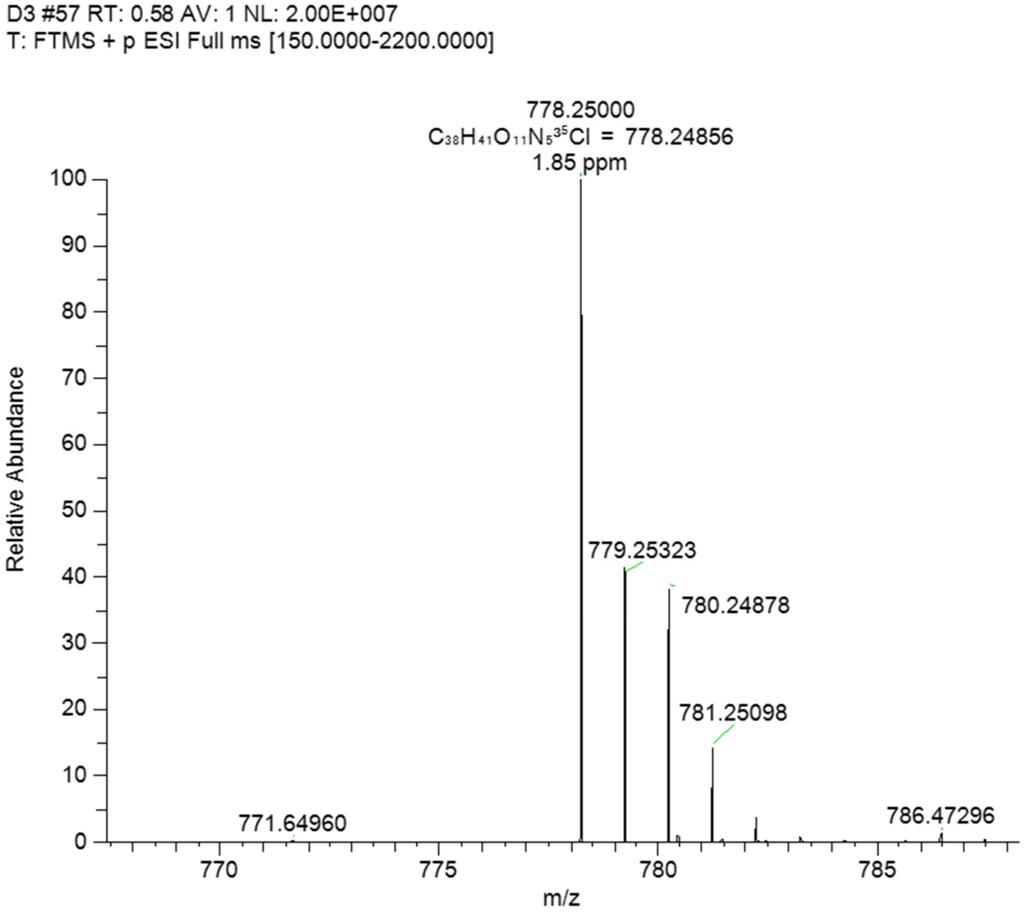

Figure S39 HRMS spectrum of title compound **D3**

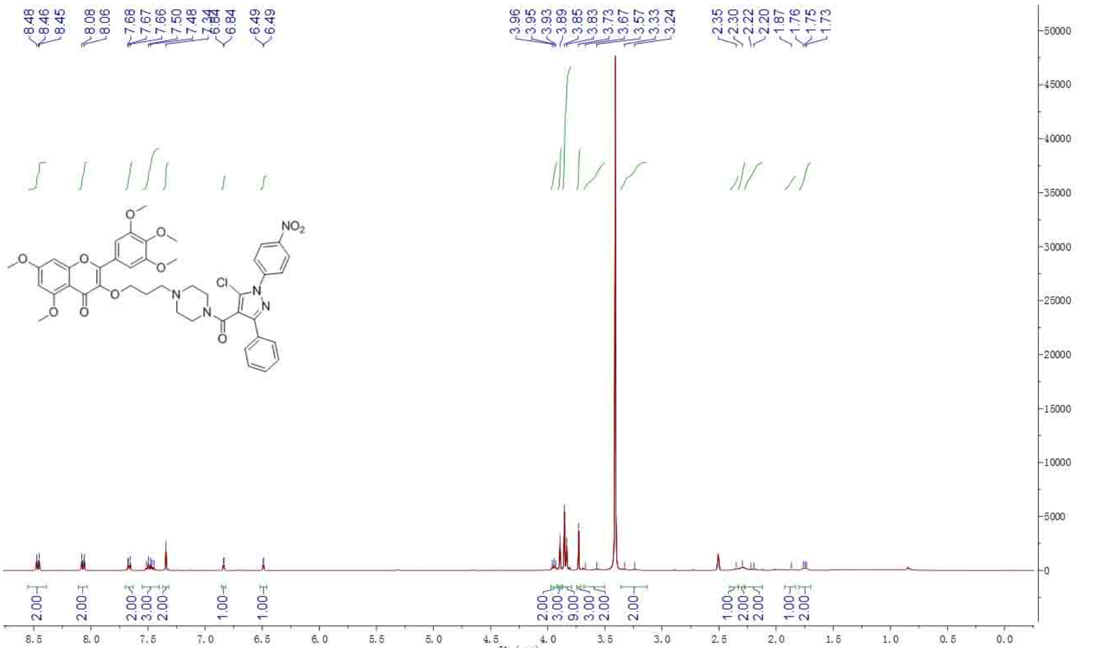

Figure S40 ^{1}H NMR spectrum of title compound **D4**

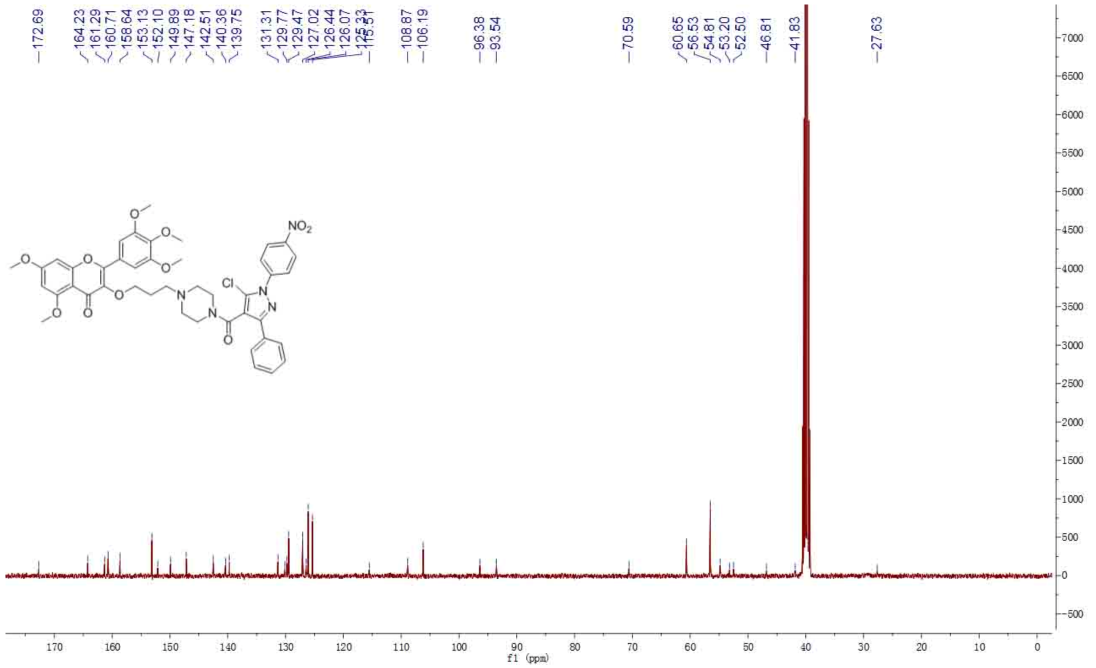

Figure S41 ^{13}C NMR spectrum of title compound **D4**

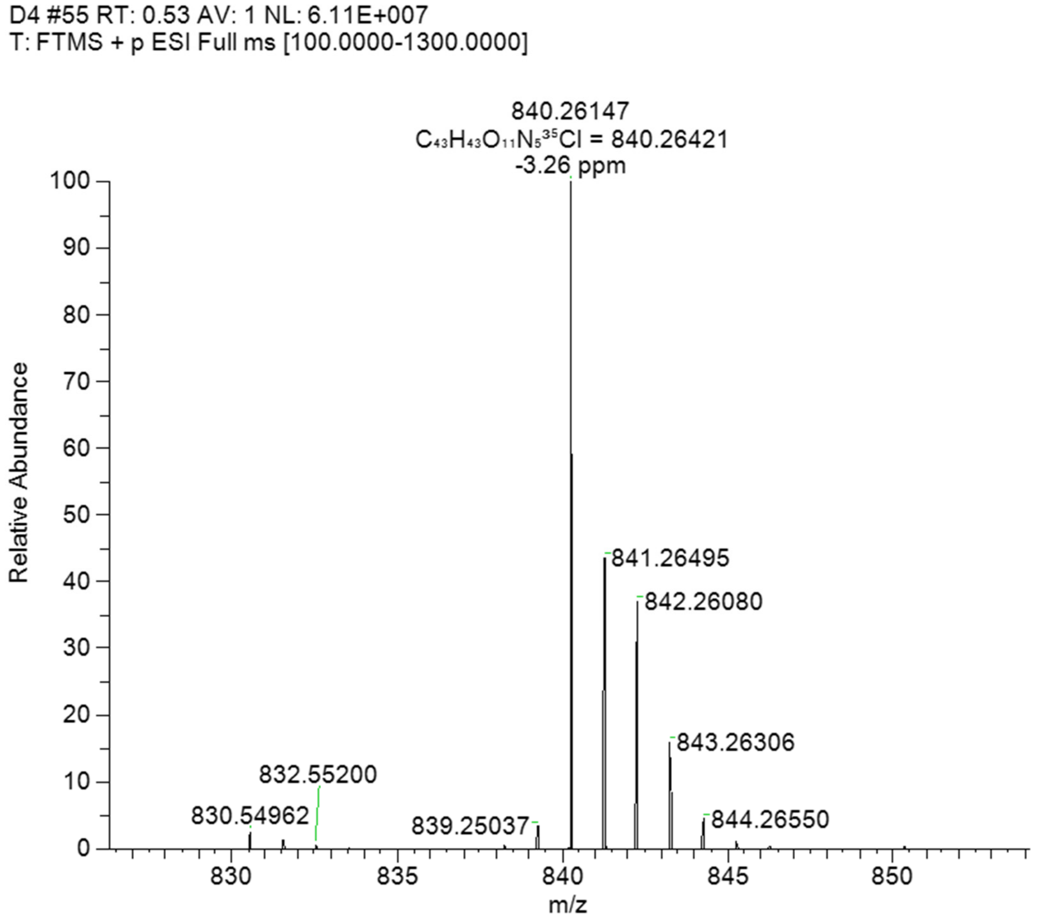

Figure S42 HRMS spectrum of title compound **D4**

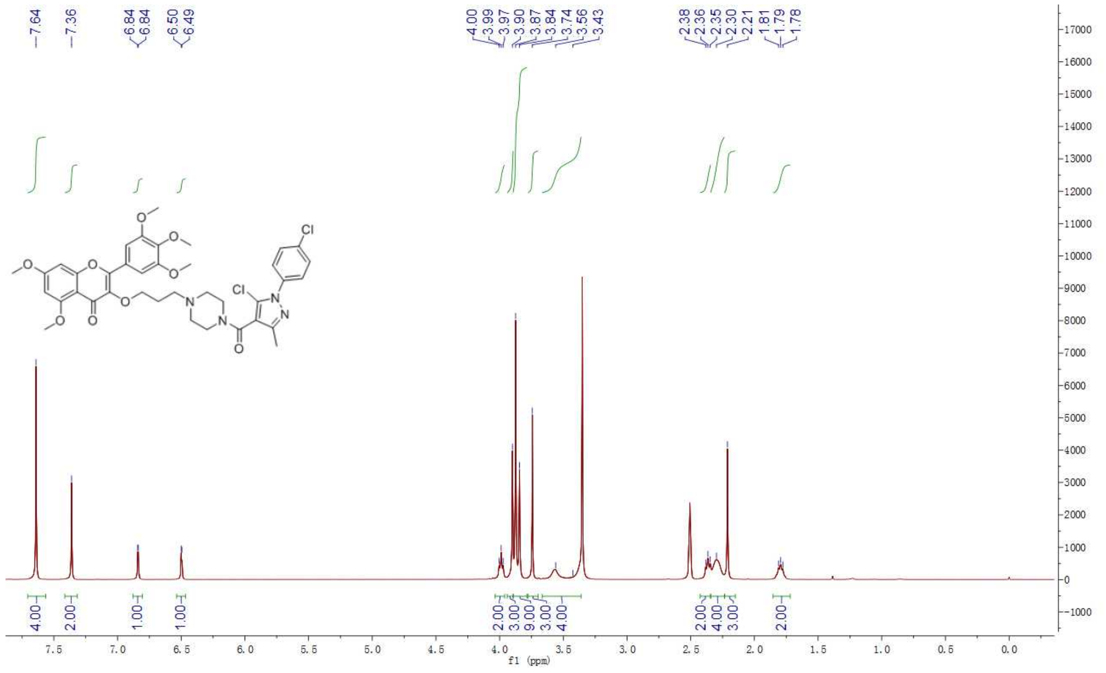

Figure S43 ^{1}H NMR spectrum of title compound **D5**

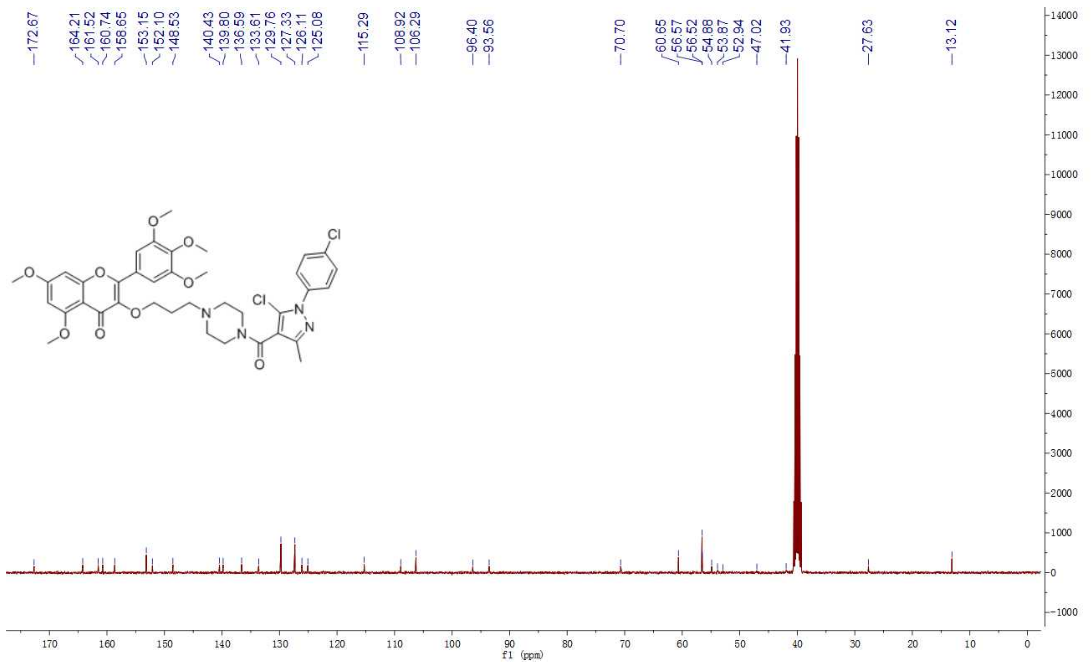

Figure S44 ^{13}C NMR spectrum of title compound **D5**

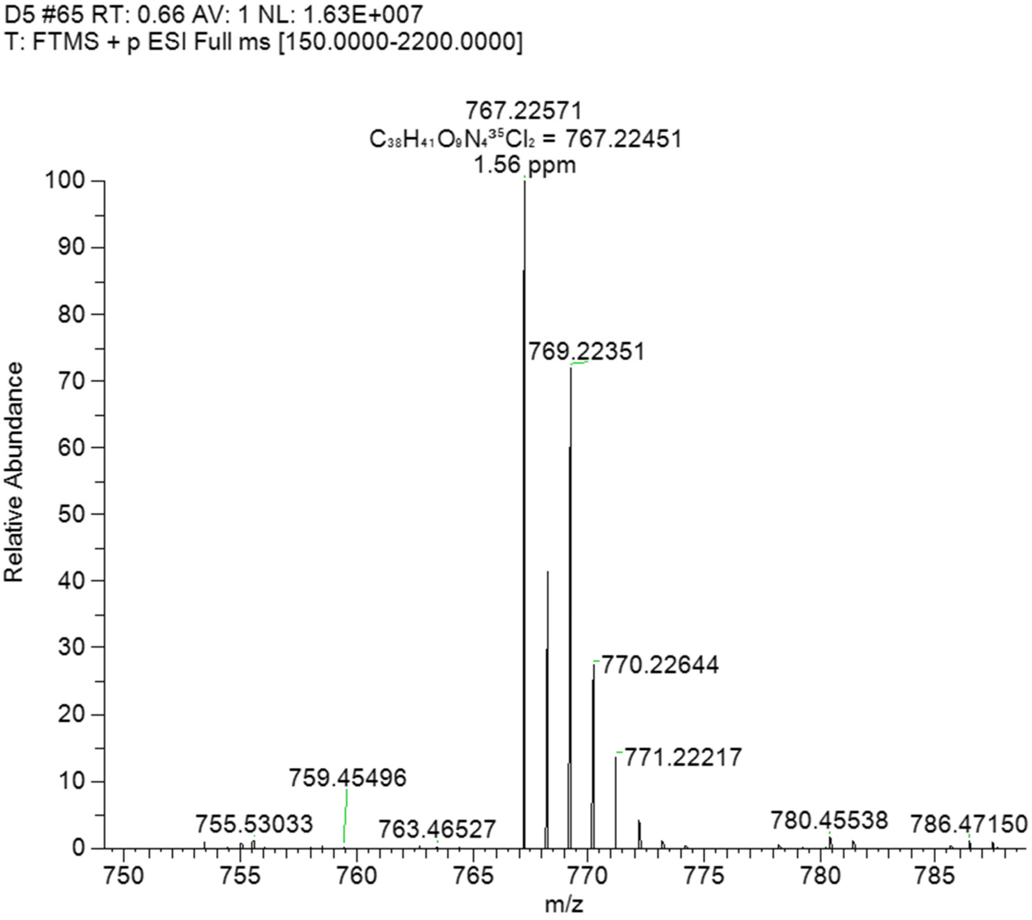

Figure S45 HRMS spectrum of title compound **D5**

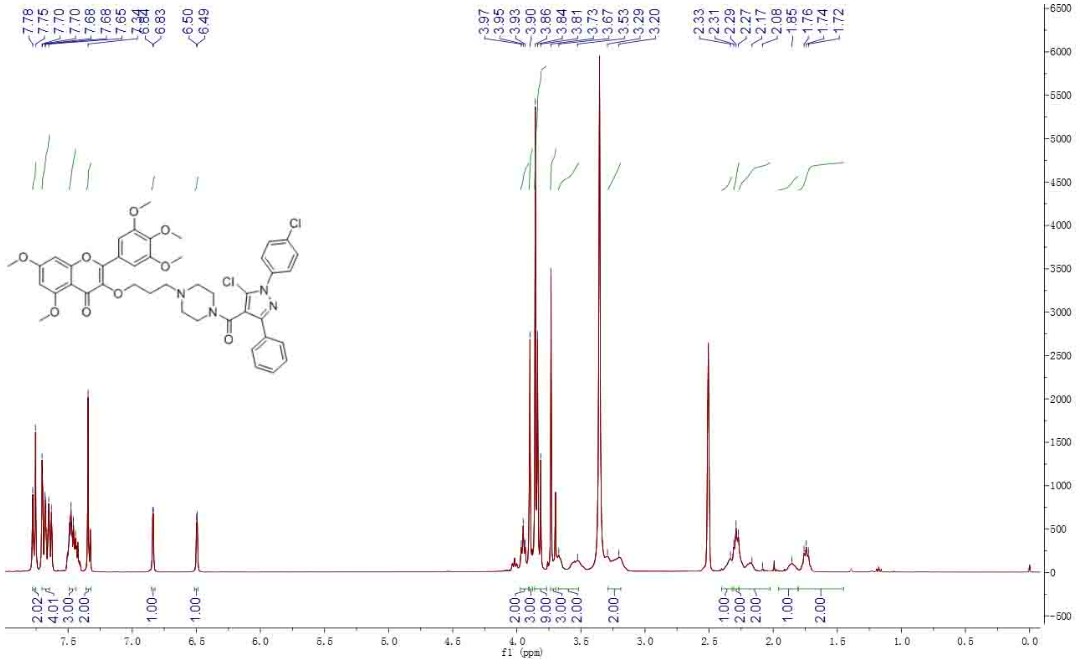

Figure S46 ^{1}H NMR spectrum of title compound **D6**

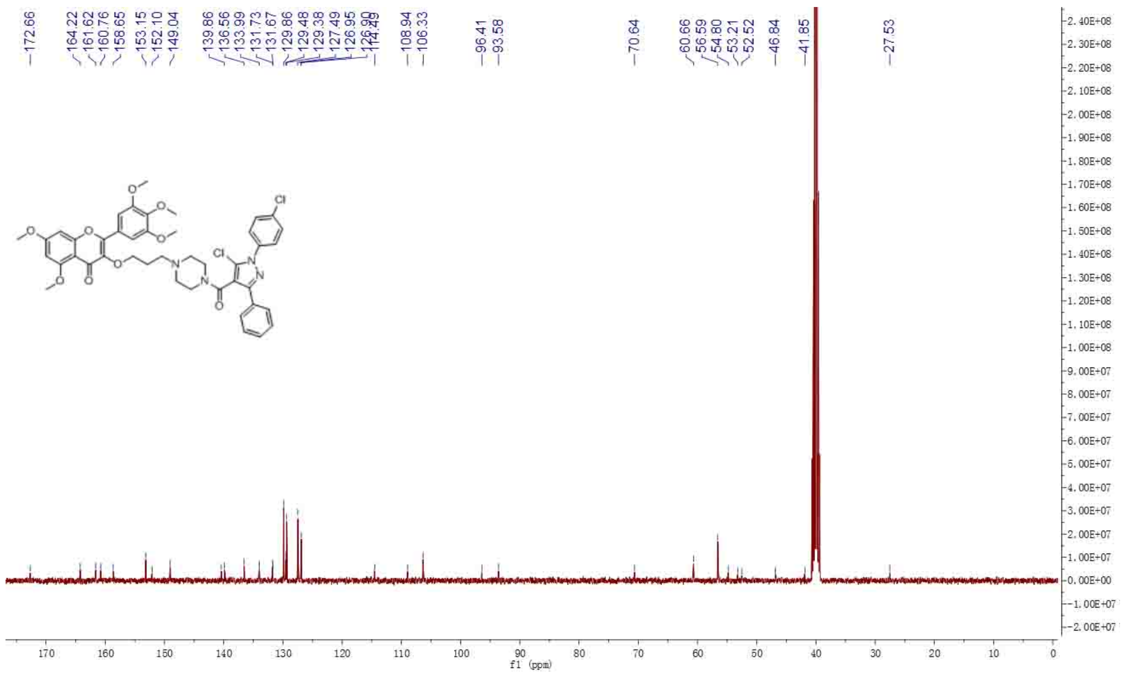

Figure S47 ^{13}C NMR spectrum of title compound **D6**

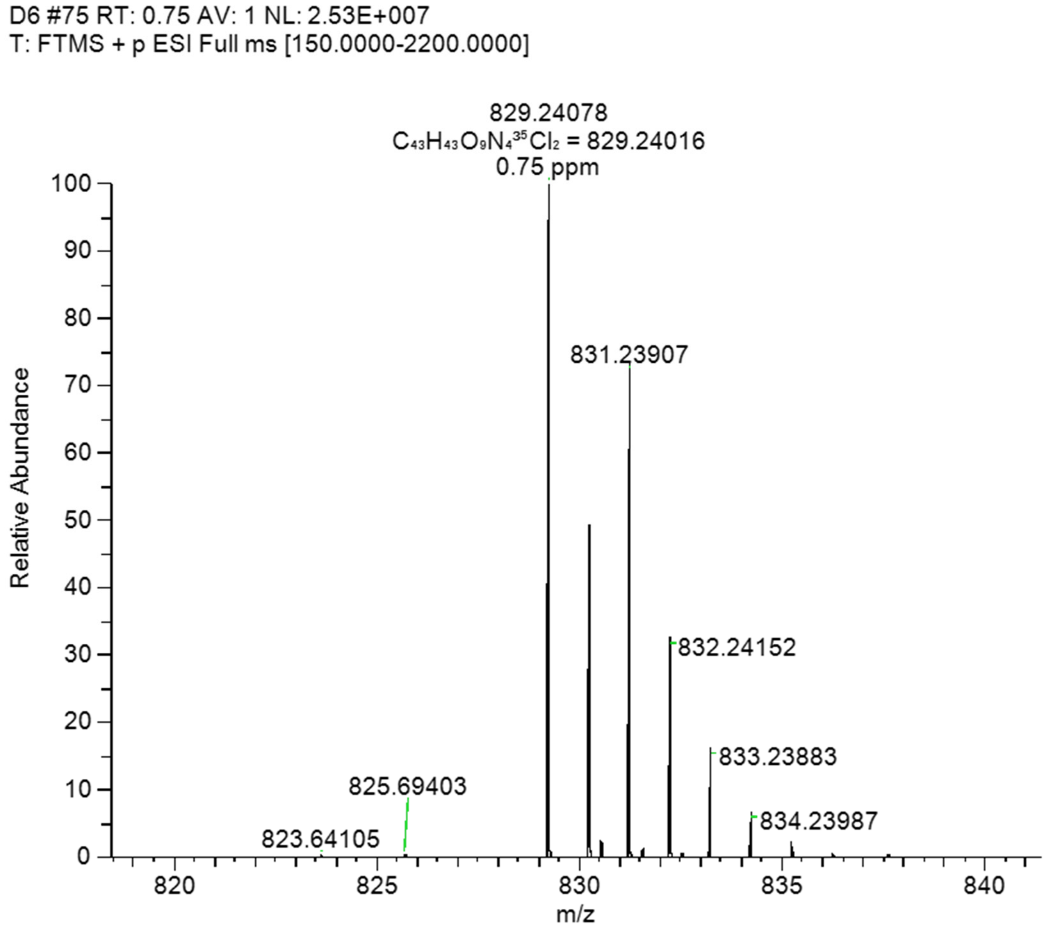

Figure S48 HRMS spectrum of title compound **D6**

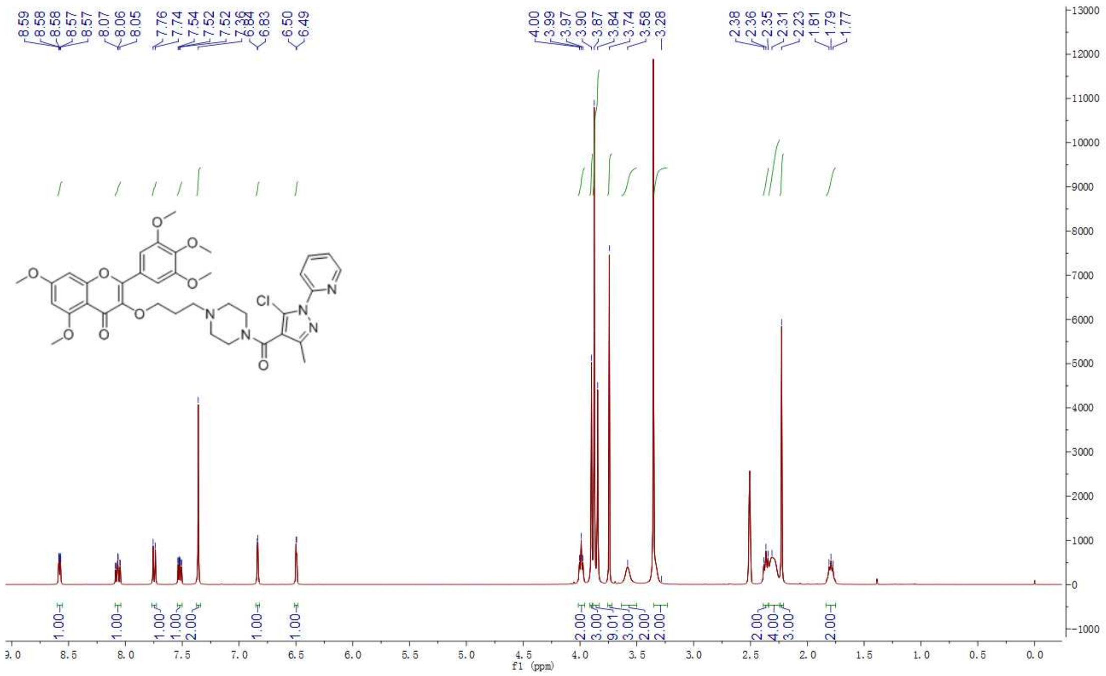

Figure S49 ^{1}H NMR spectrum of title compound **D7**

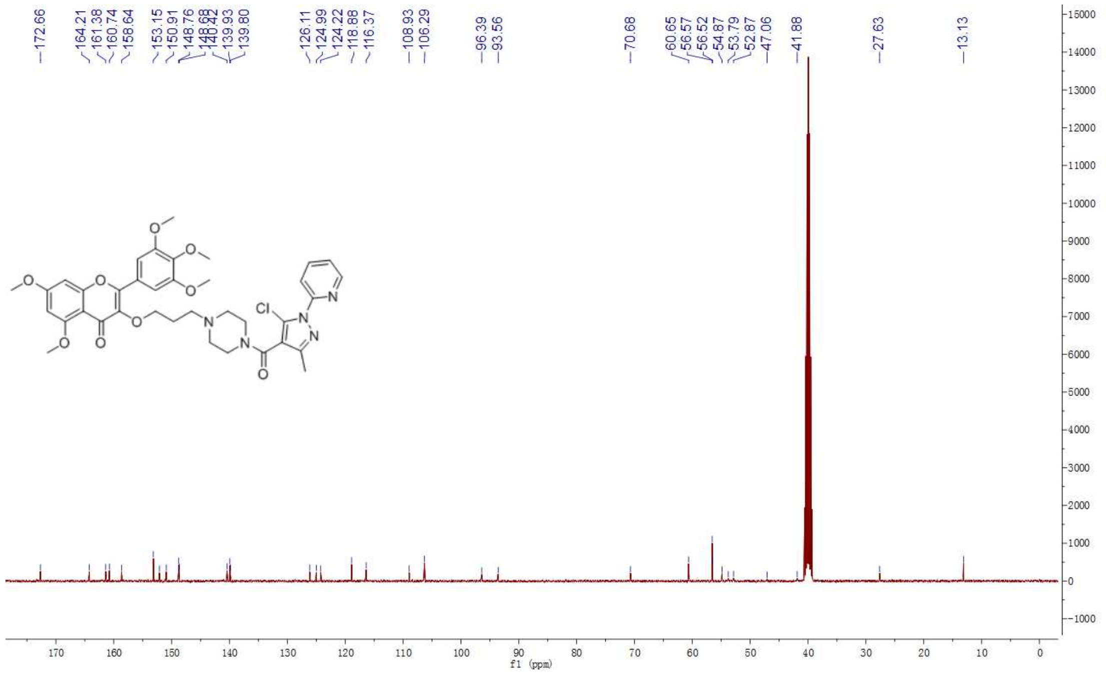

Figure S50 ^{13}C NMR spectrum of title compound **D7**

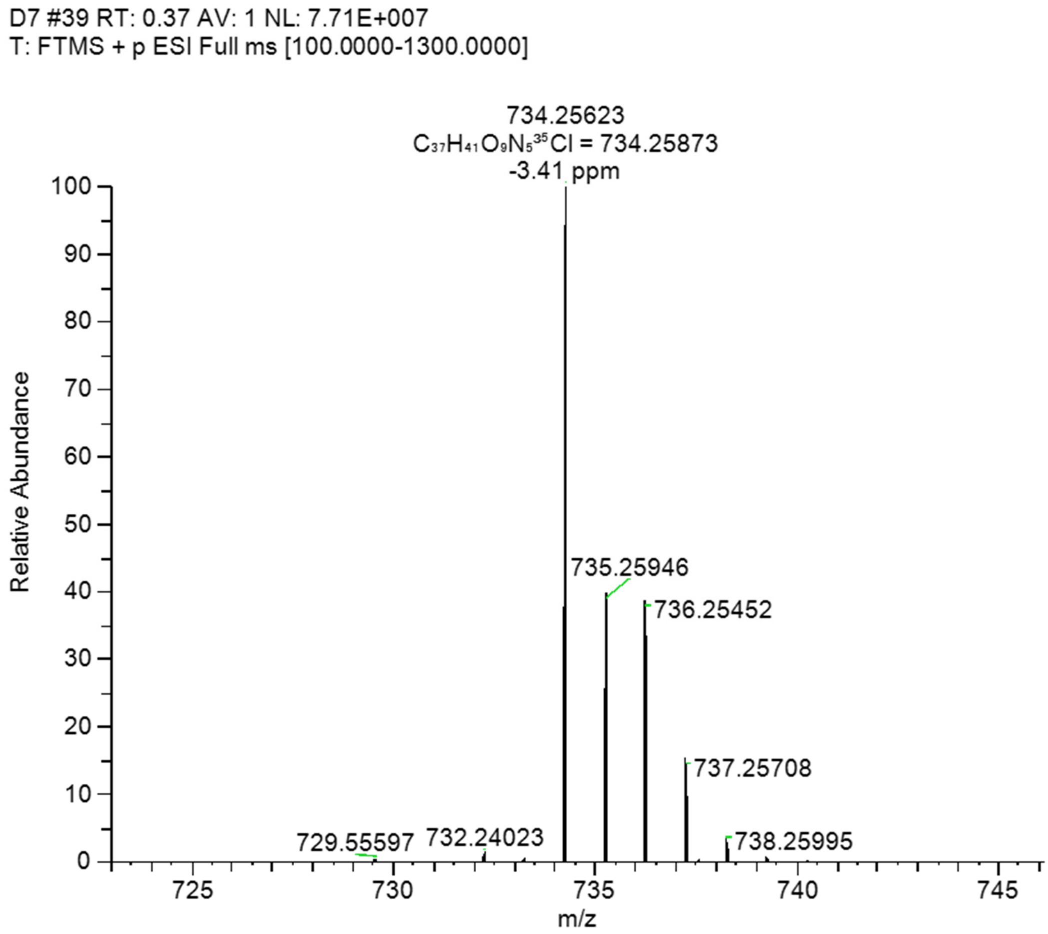

Figure S51 HRMS spectrum of title compound **D7**

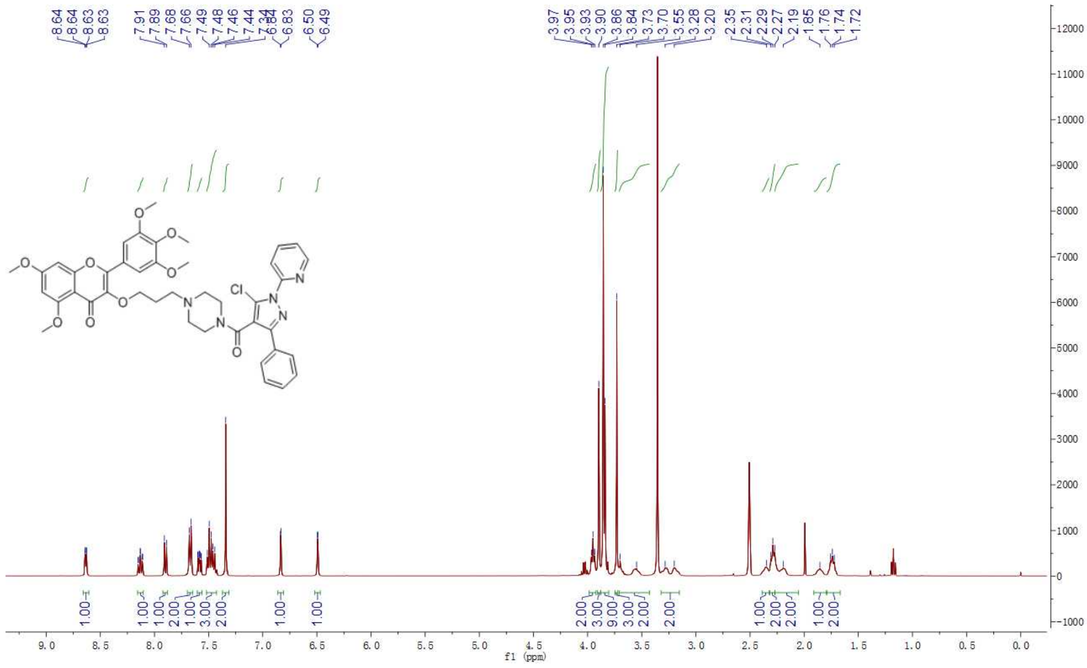

Figure S52 ^{1}H NMR spectrum of title compound **D8**

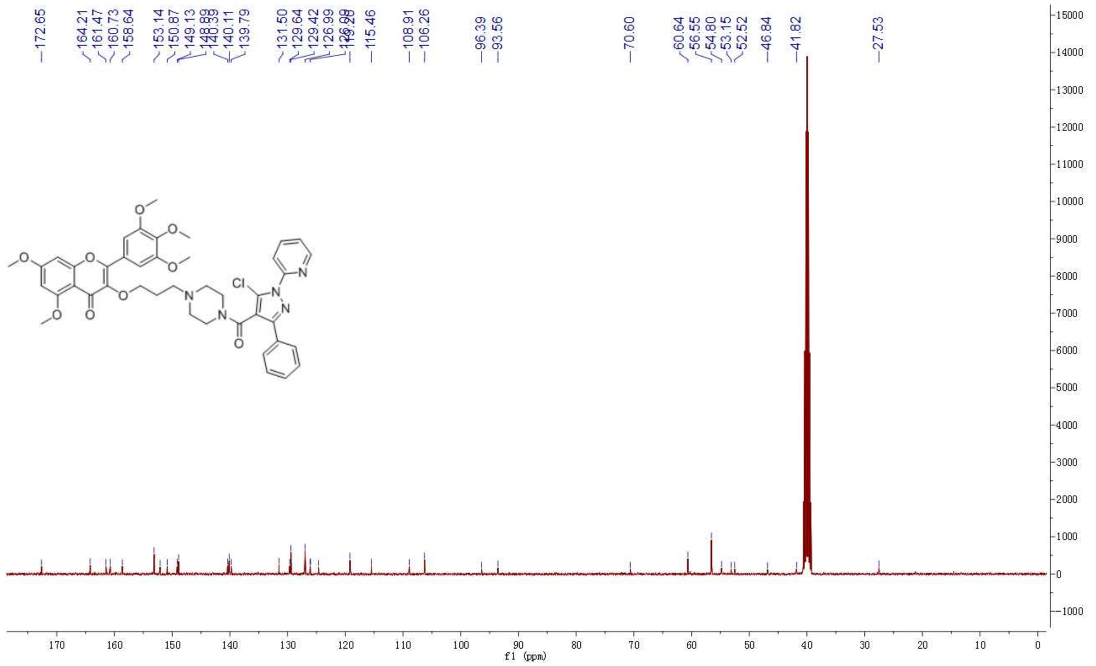

Figure S53 ^{13}C NMR spectrum of title compound **D8**

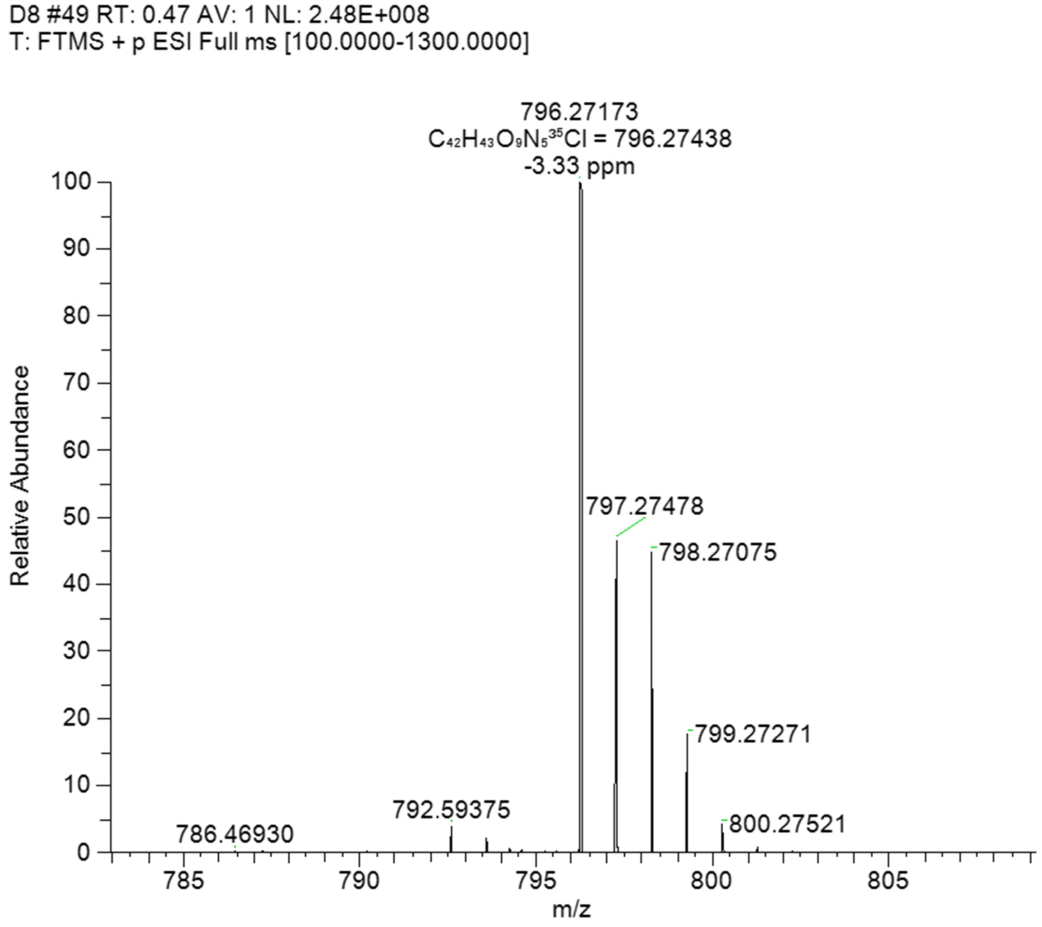

Figure S54 HRMS spectrum of title compound **D8**

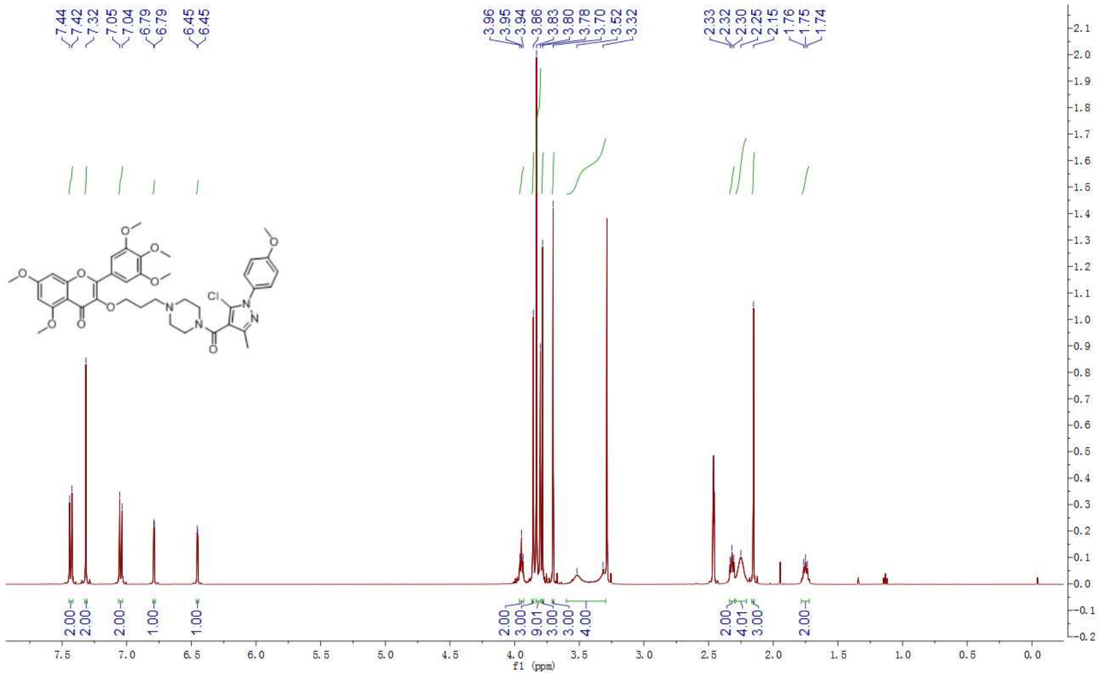

Figure S55 ^{1}H NMR spectrum of title compound **D9**

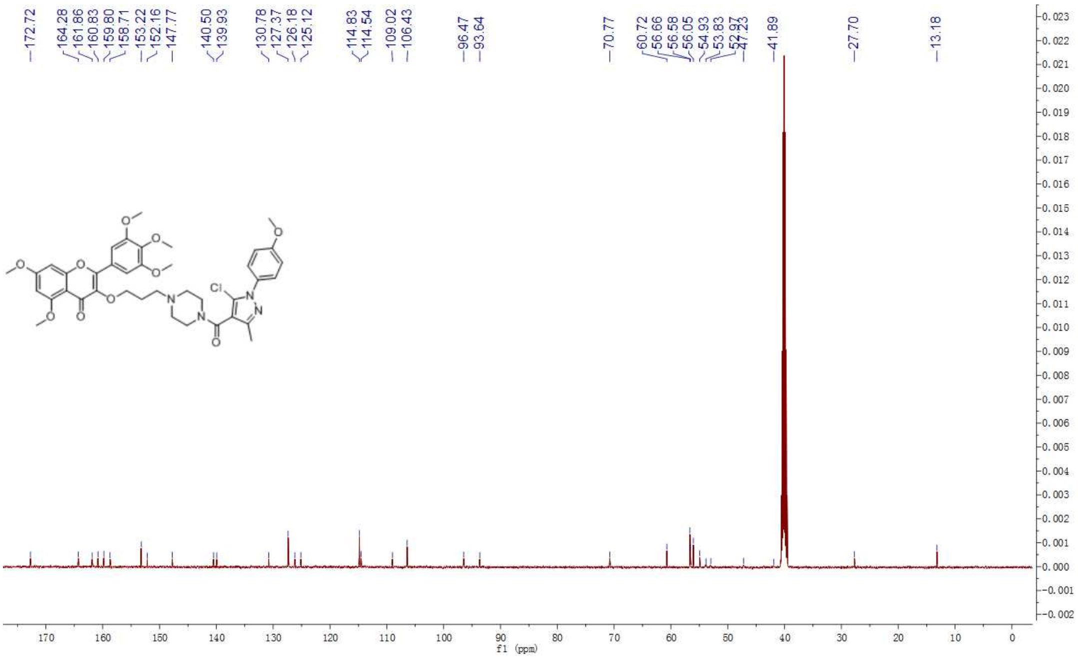

Figure S56 ^{13}C NMR spectrum of title compound **D9**

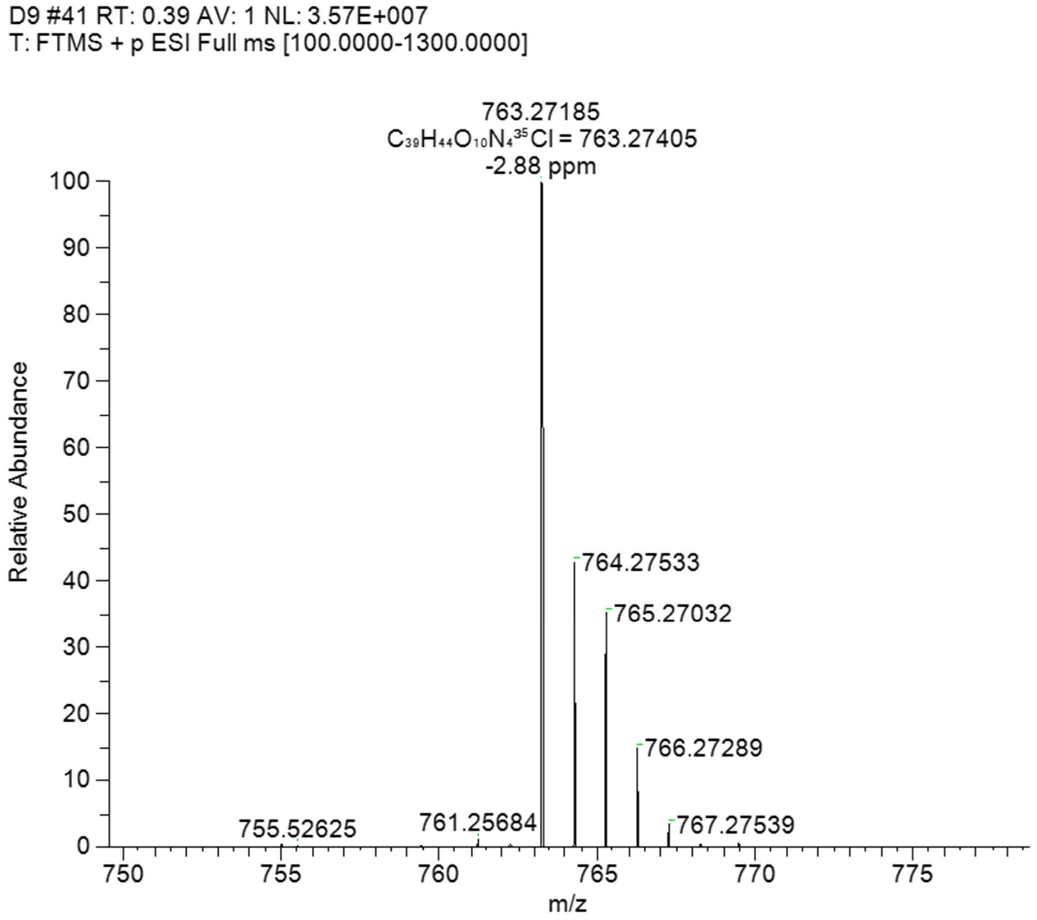

Figure S57 HRMS spectrum of title compound **D9**

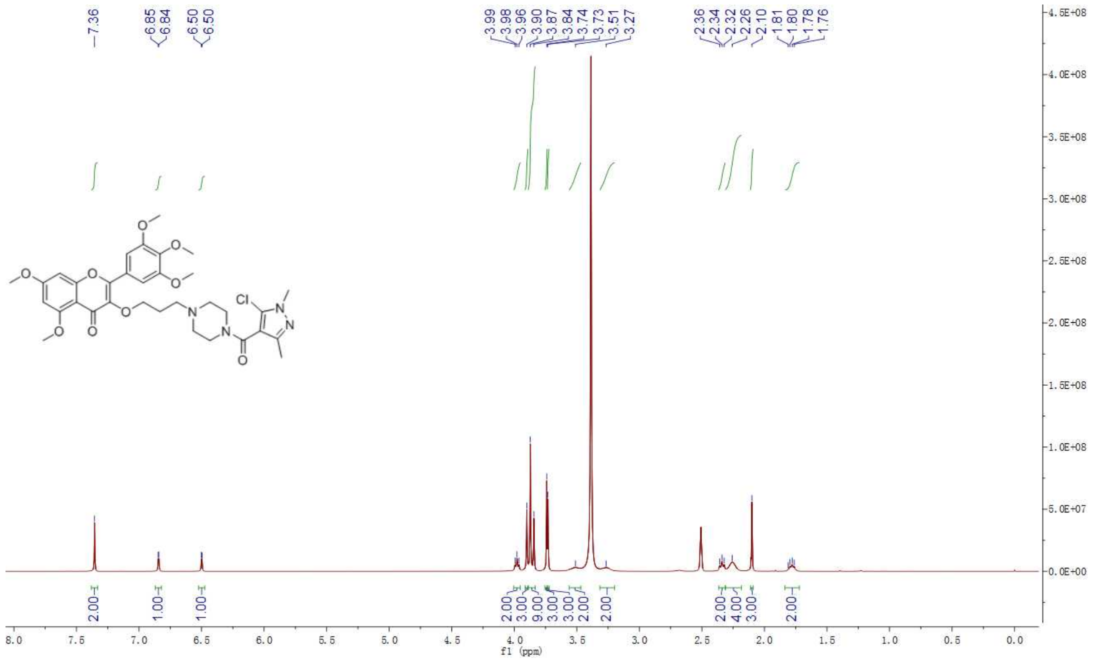

Figure S58 ^{1}H NMR spectrum of title compound **D10**

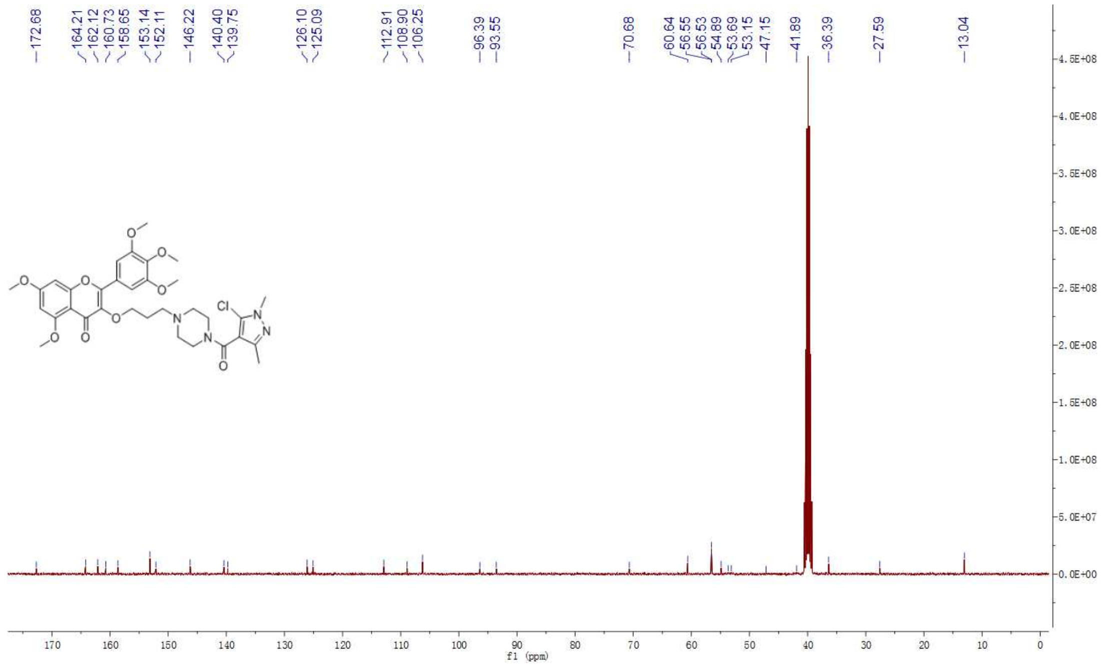

Figure S59 ^{13}C NMR spectrum of title compound **D10**

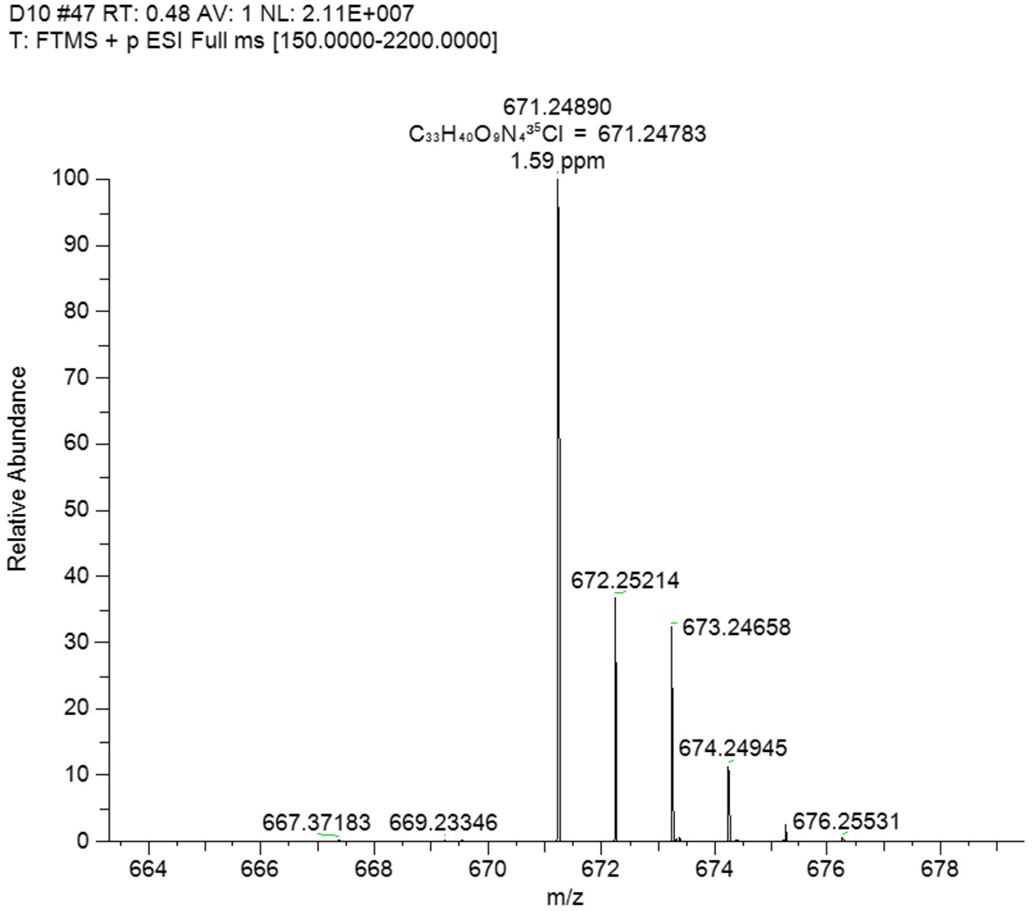

Figure S60 HRMS spectrum of title compound **D10**

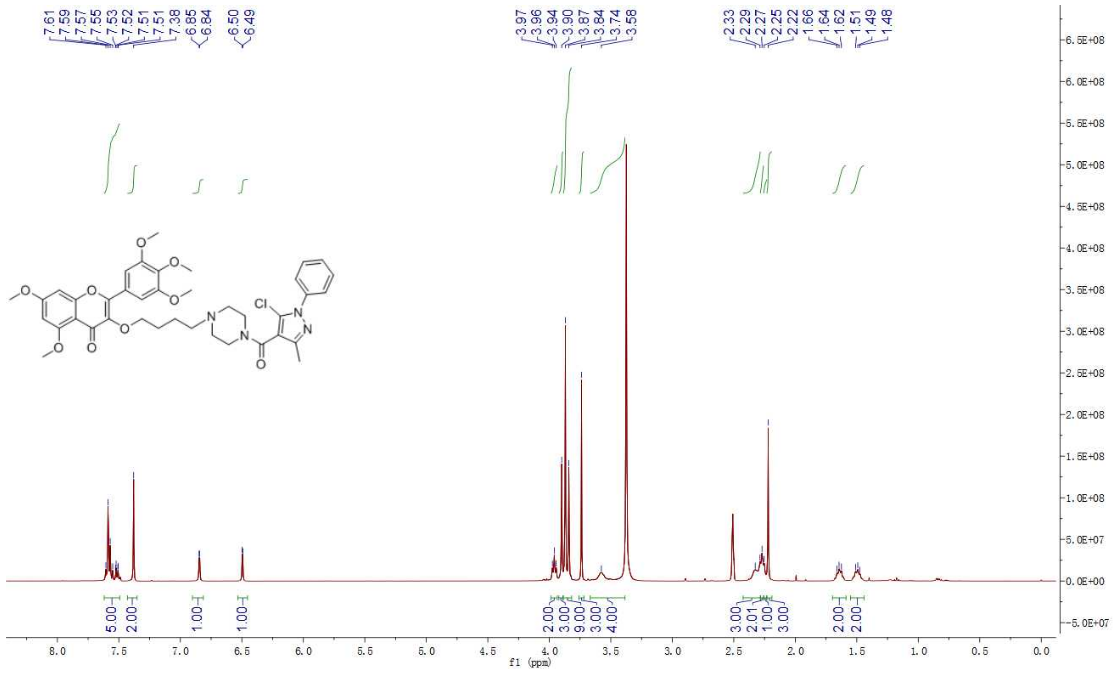

Figure S61 ^{1}H NMR spectrum of title compound **D11**

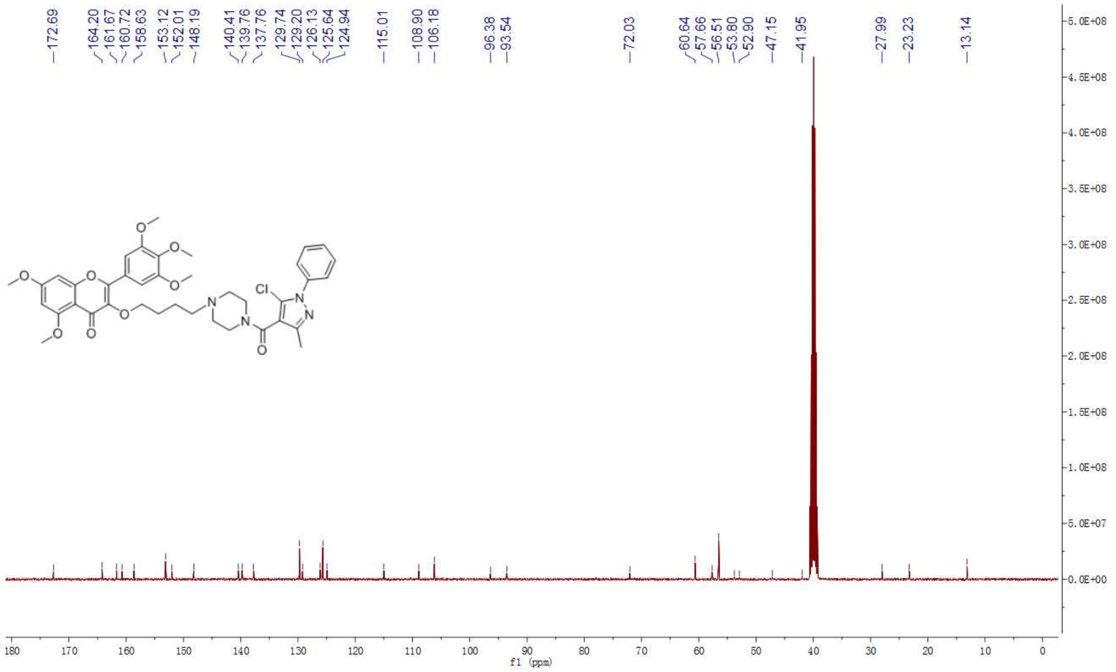

Figure S62 ^{13}C NMR spectrum of title compound **D11**

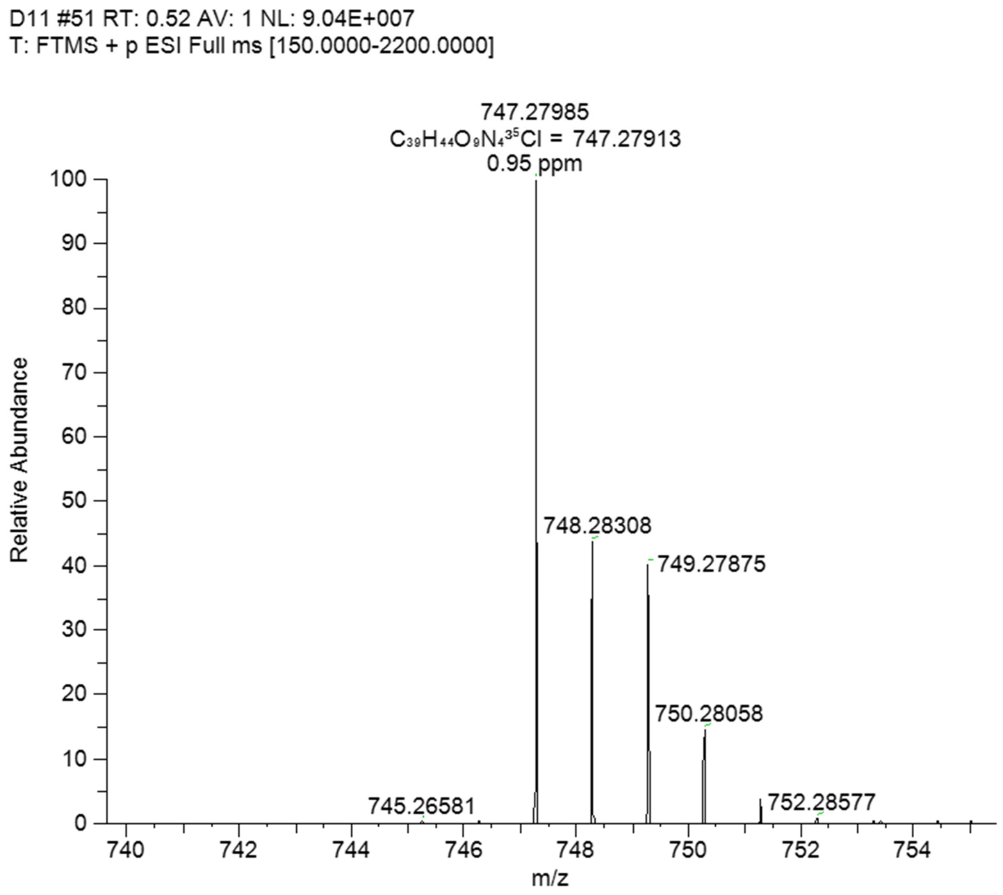

Figure S63 HRMS spectrum of title compound **D11**

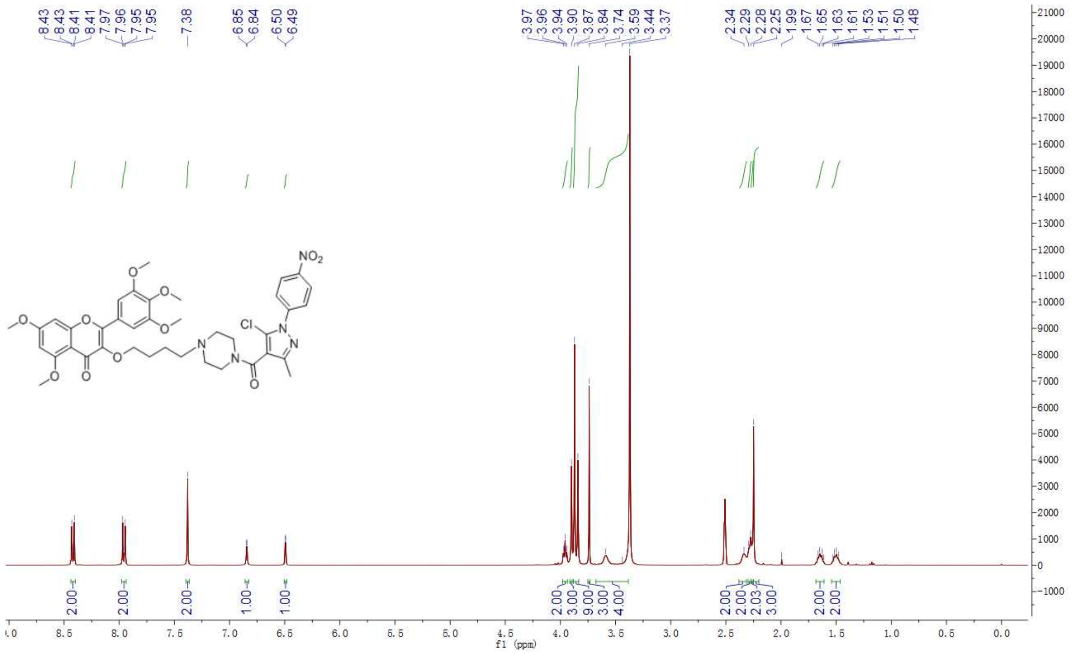

Figure S64 ^{1}H NMR spectrum of title compound **D12**

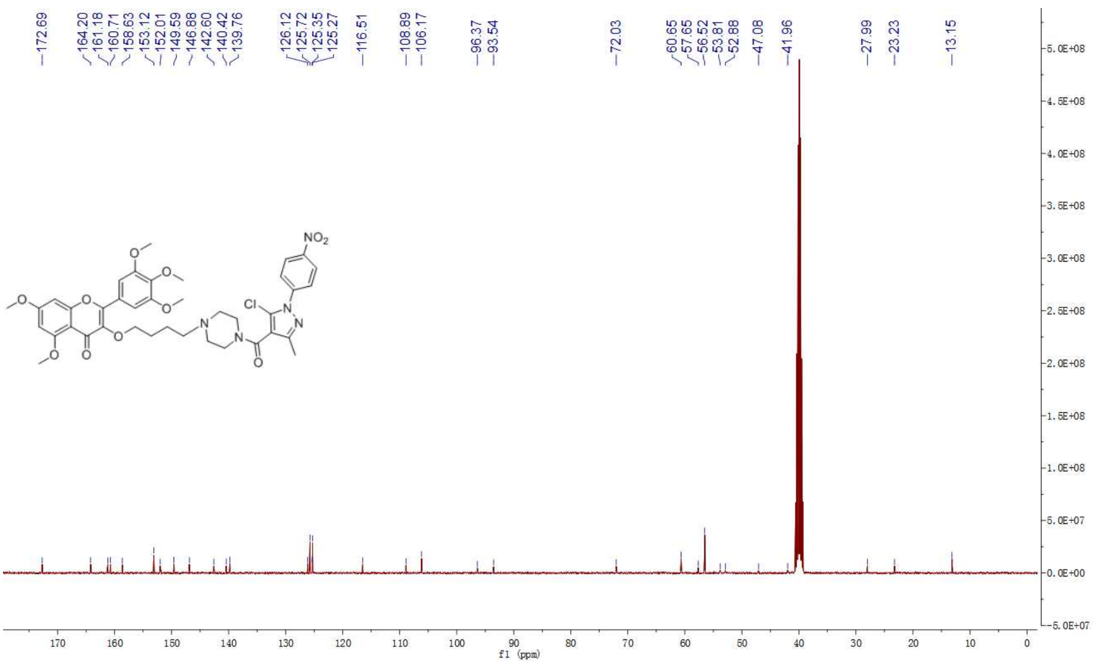

Figure S65 ^{13}C NMR spectrum of title compound **D12**

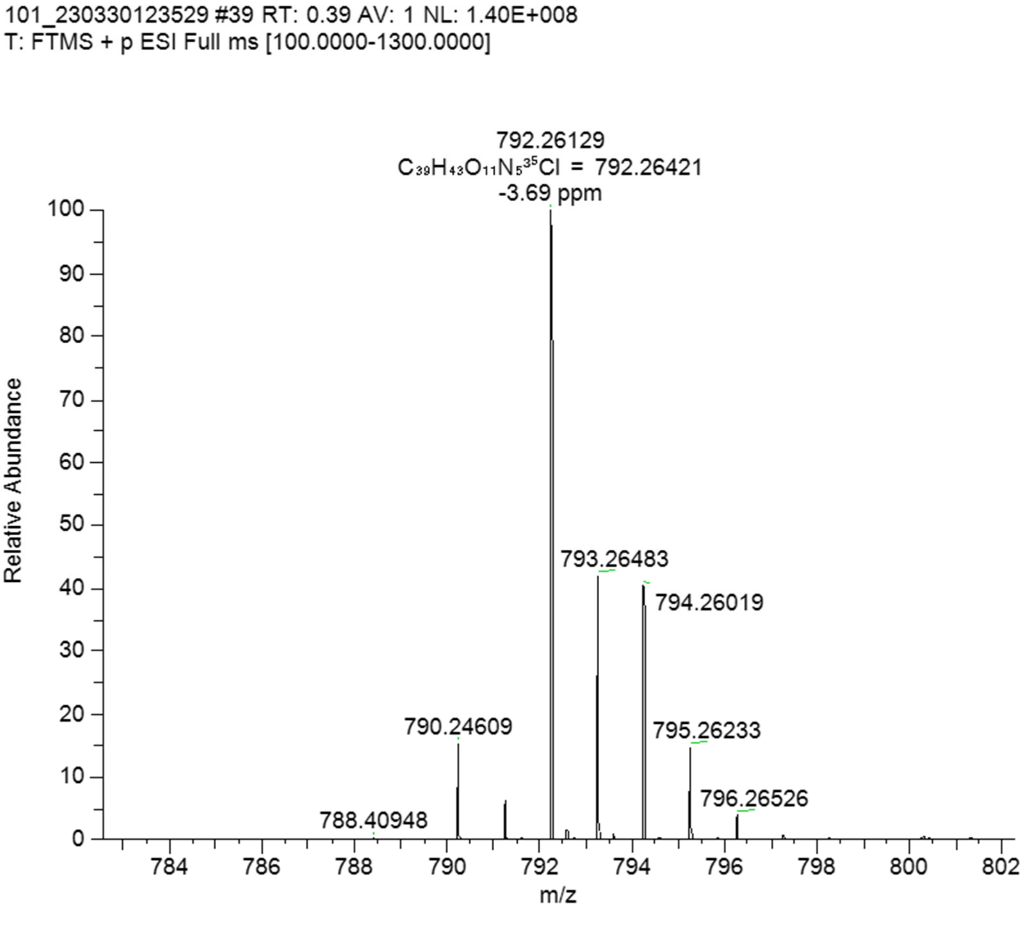

Figure S66 HRMS spectrum of title compound **D12**

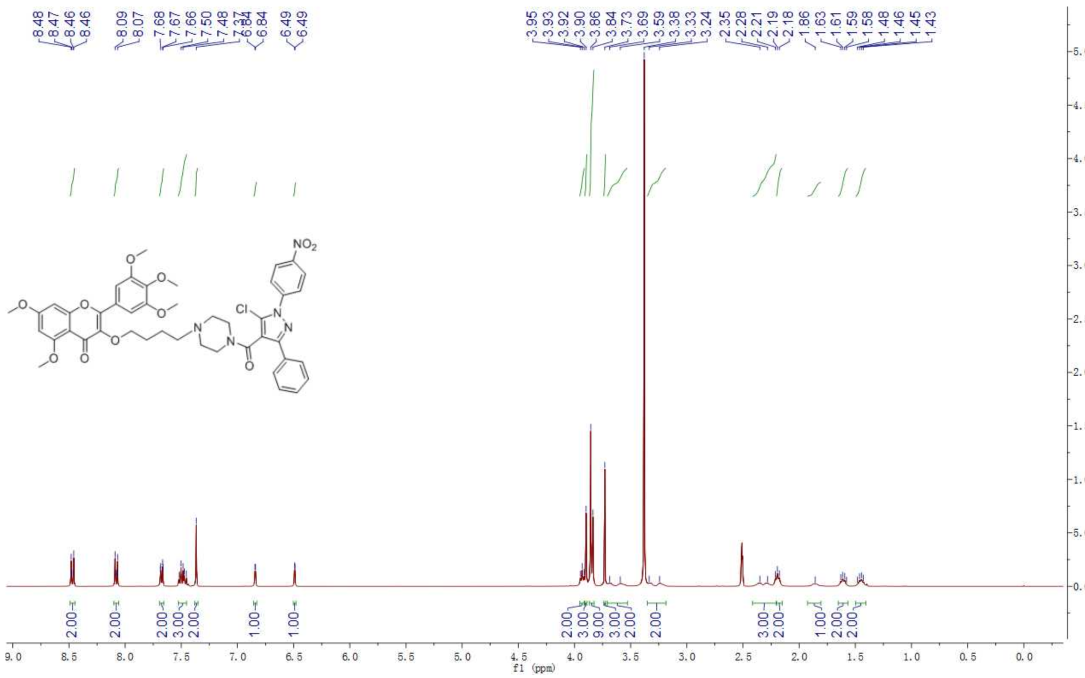

Figure S67 ^{1}H NMR spectrum of title compound **D13**

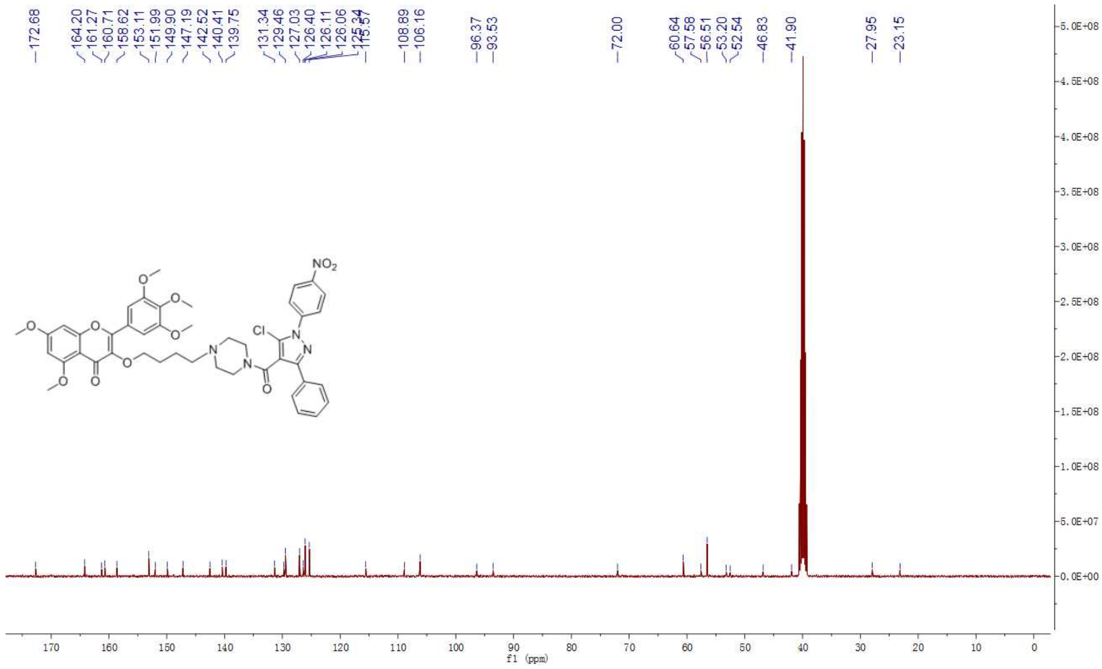

Figure S68 ^{13}C NMR spectrum of title compound **D13**

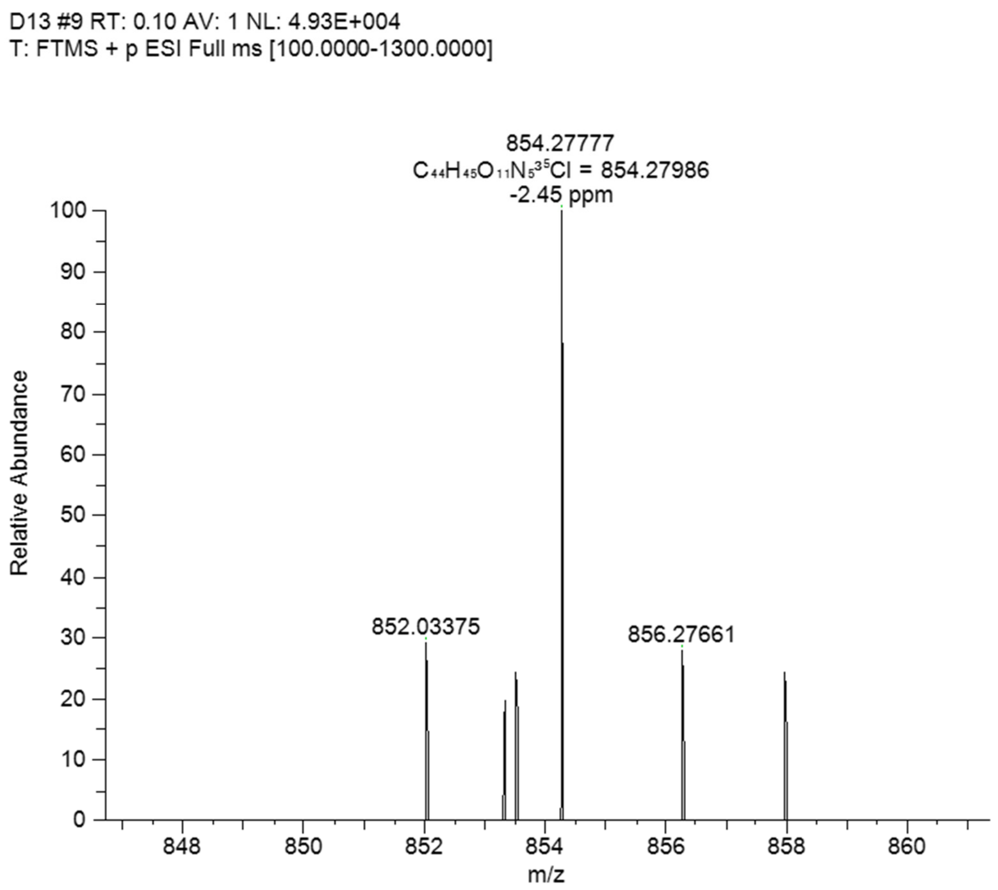

Figure S69 HRMS spectrum of title compound **D13**

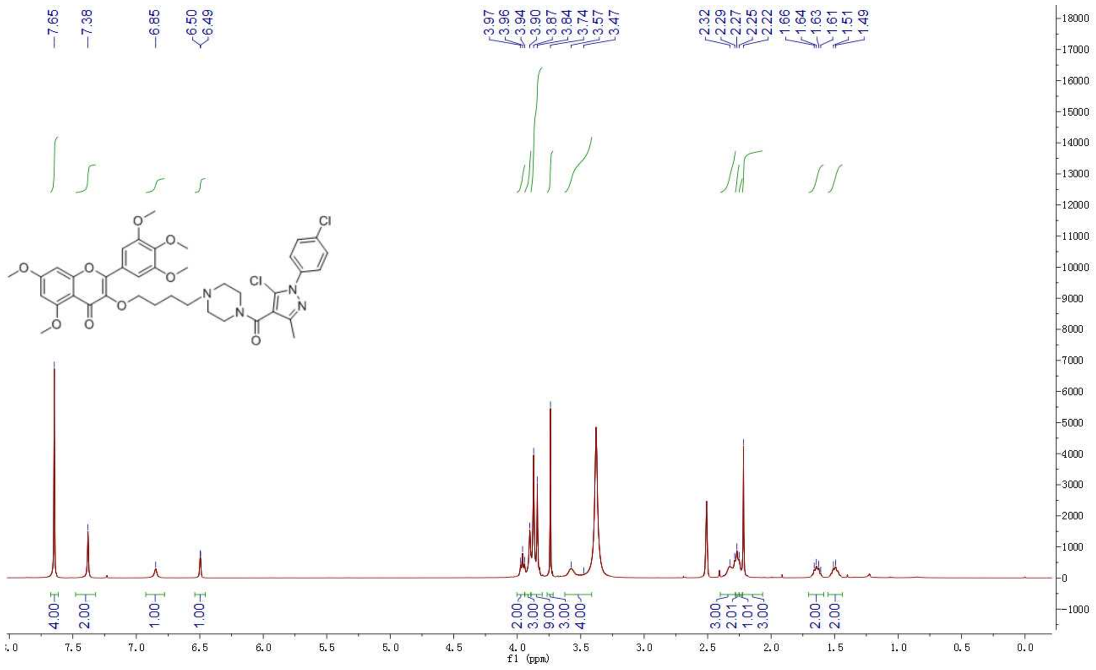

Figure S70 ^{1}H NMR spectrum of title compound **D14**

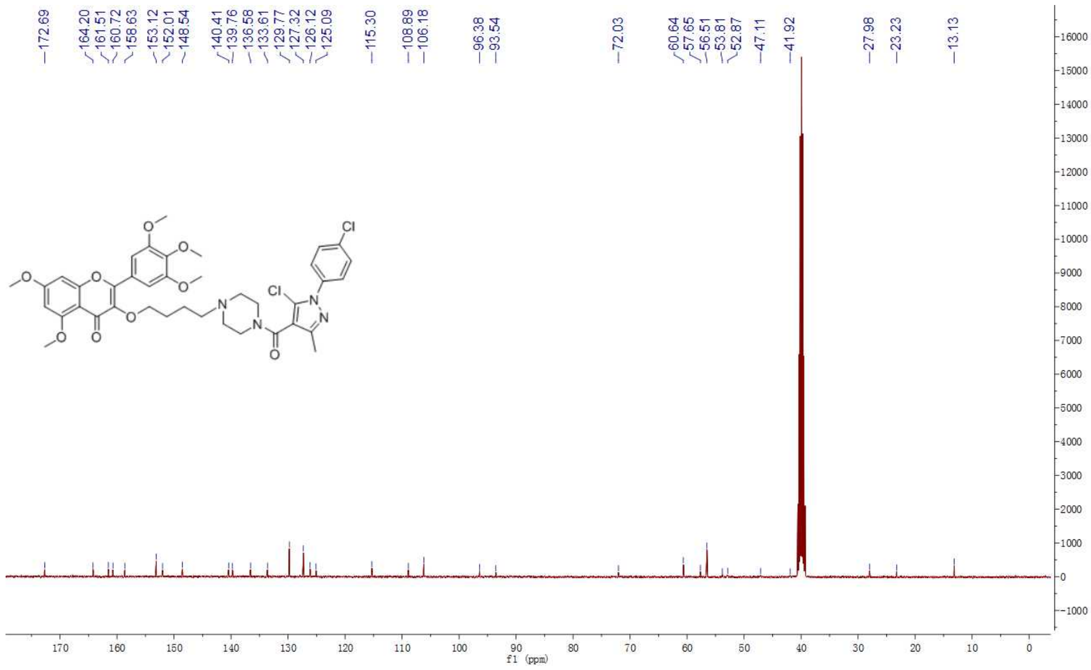

Figure S71^{13}C NMR spectrum of title compound **D14**

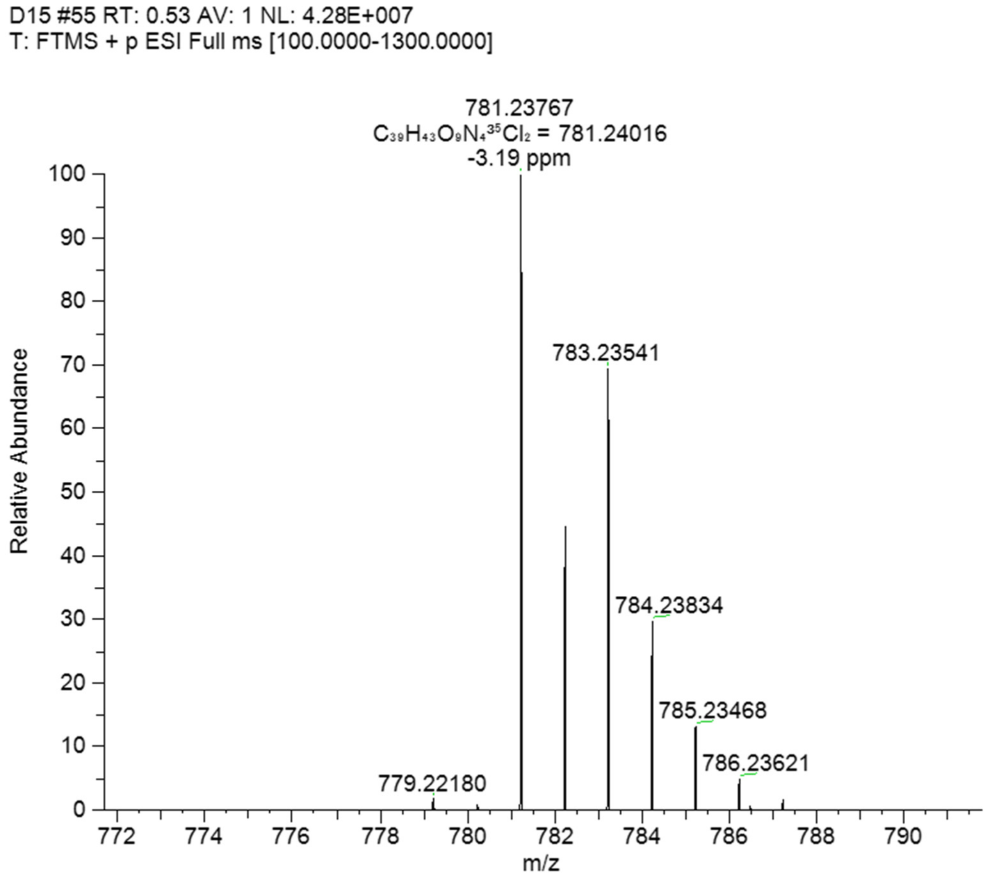

Figure S72 HRMS spectrum of title compound **D14**

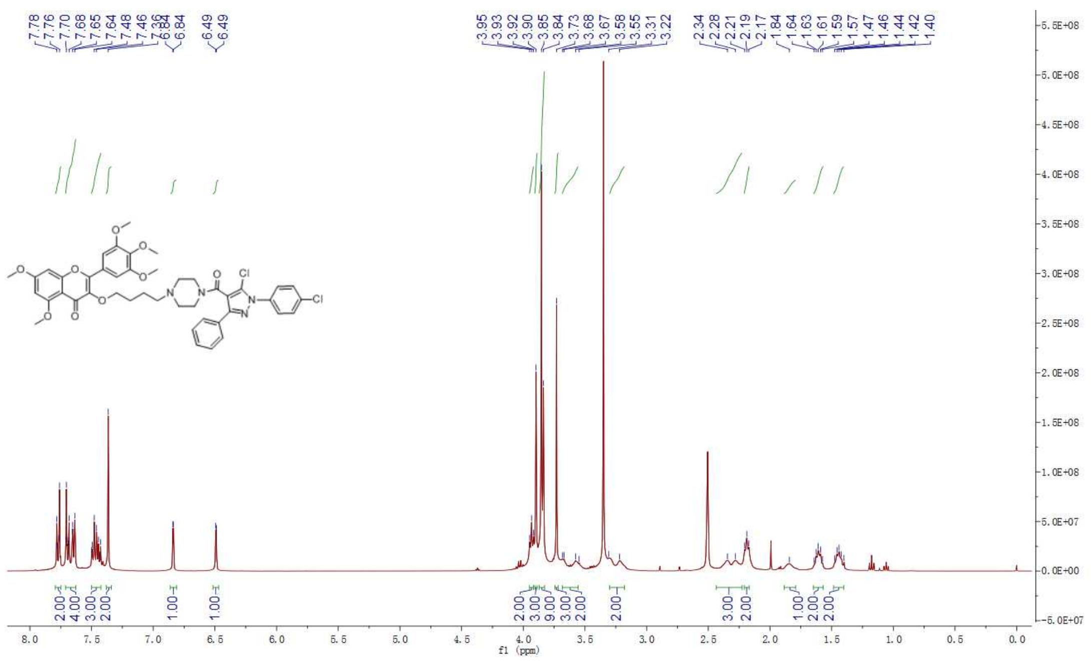

Figure S73 ^{1}H NMR spectrum of title compound **D15**

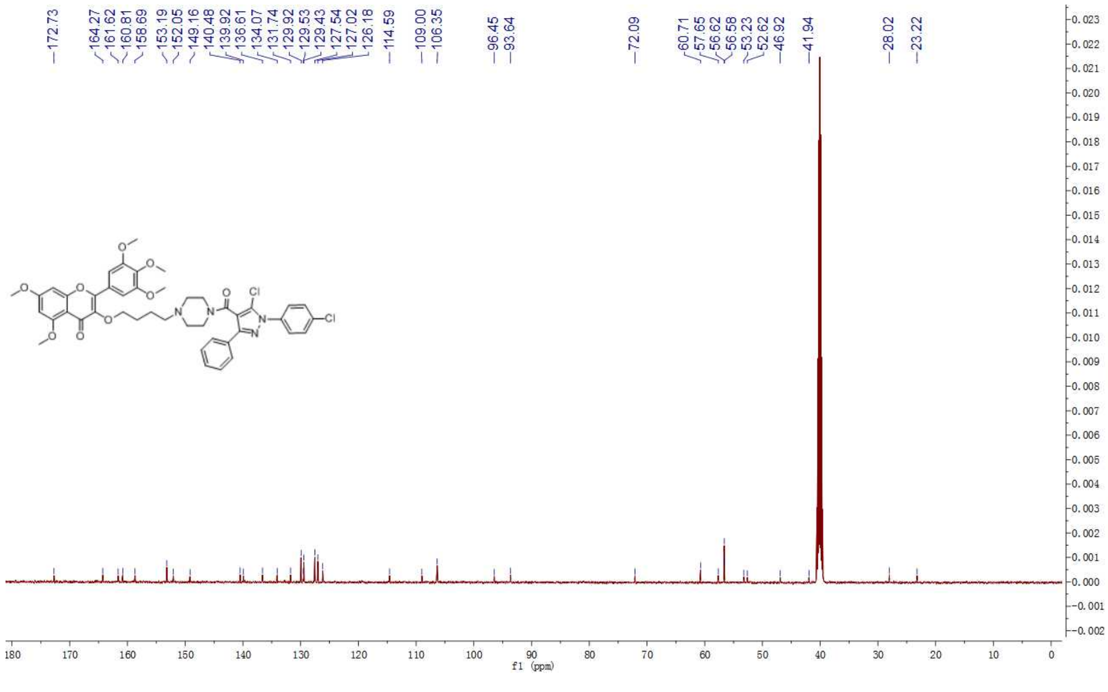

Figure S74 ^{13}C NMR spectrum of title compound **D15**

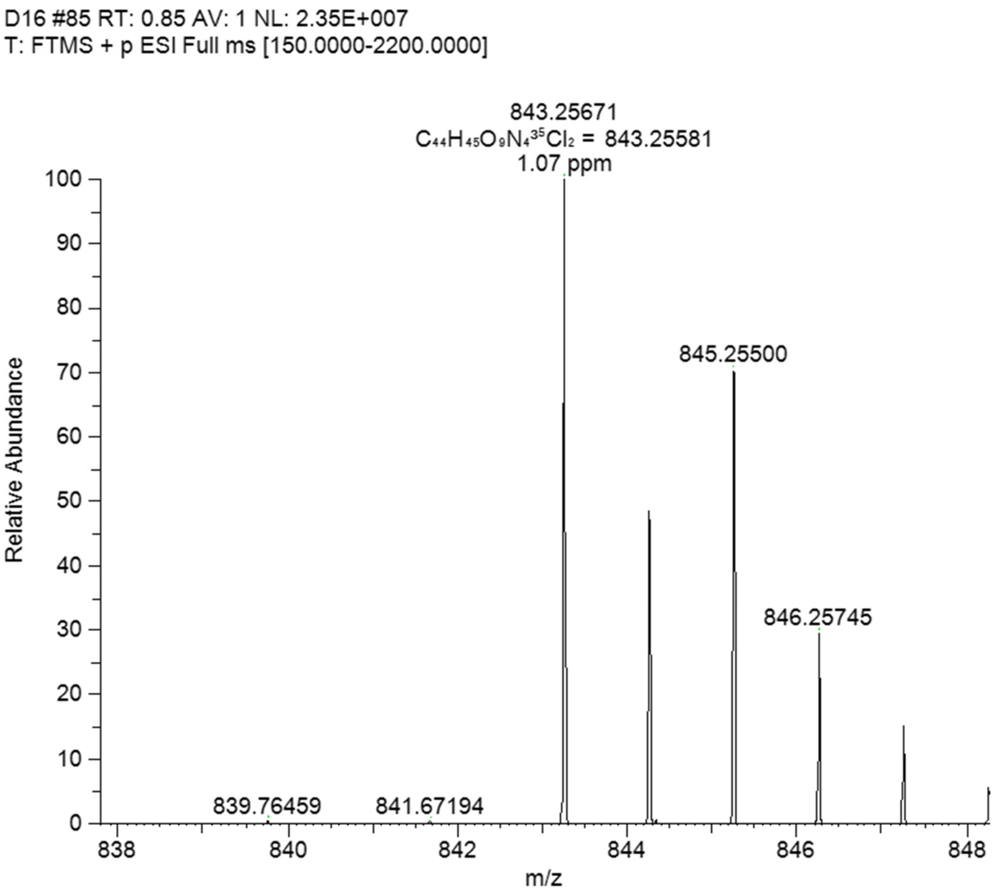

Figure S75 HRMS spectrum of title compound **D15**

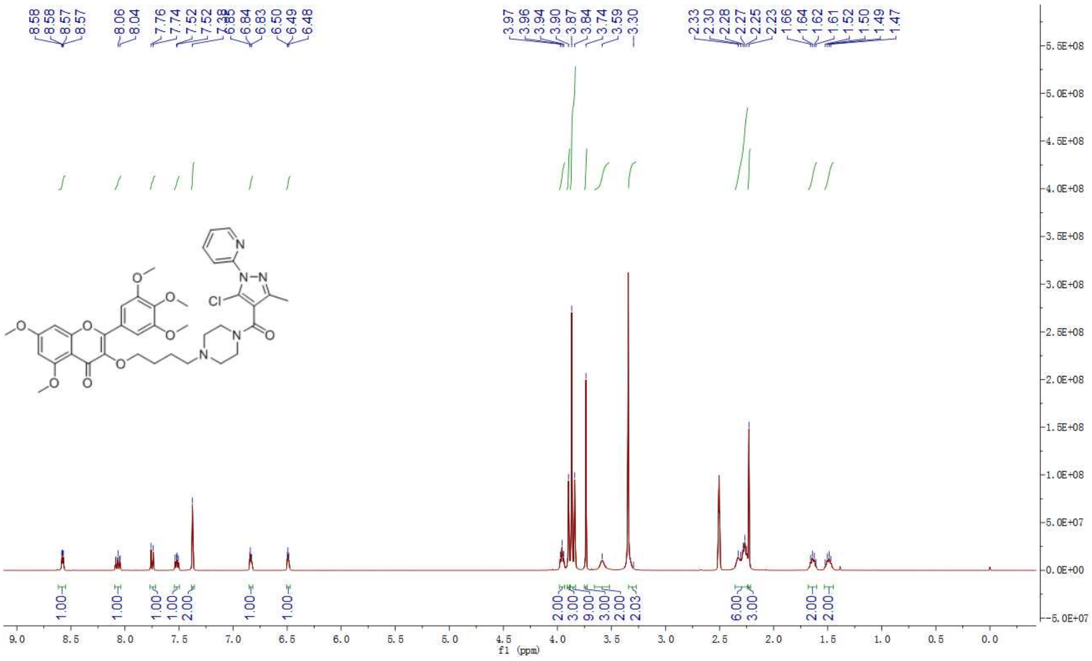

Figure S76 ^{1}H NMR spectrum of title compound **D16**

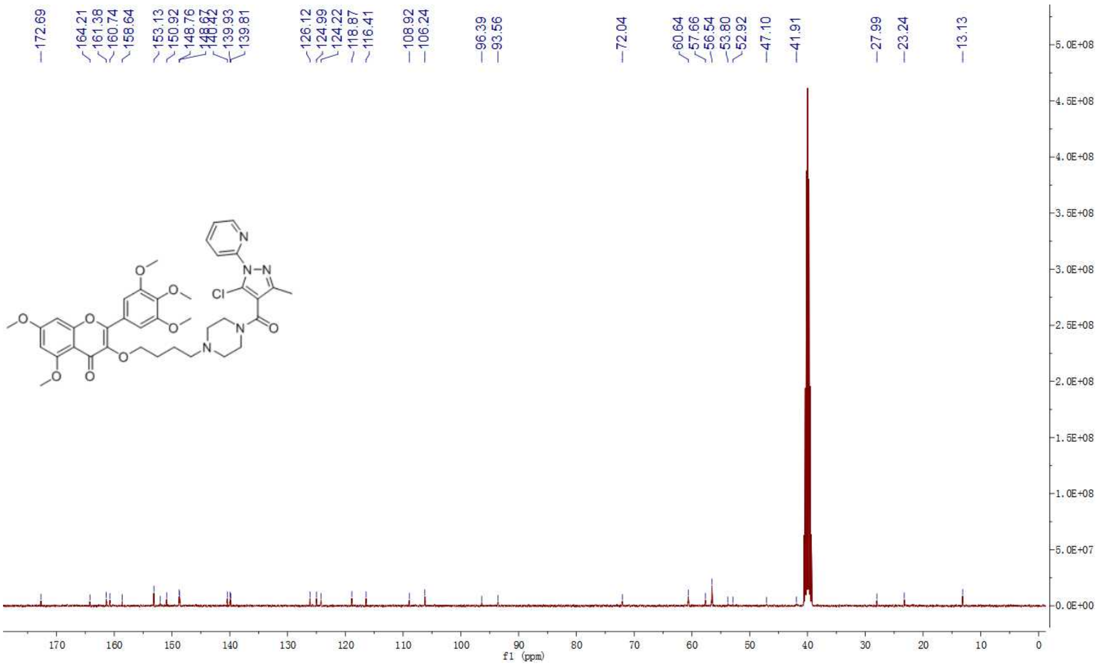

Figure S77 ^{13}C NMR spectrum of title compound **D16**

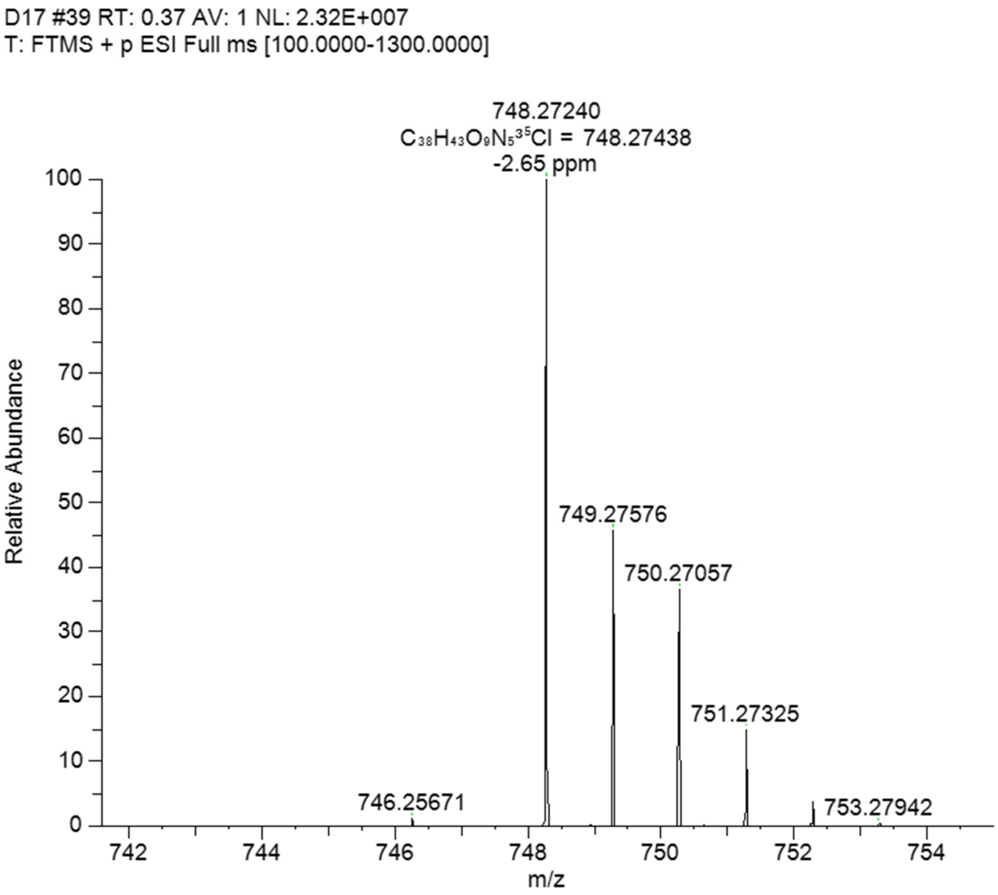

Figure S78 HRMS spectrum of title compound **D16**

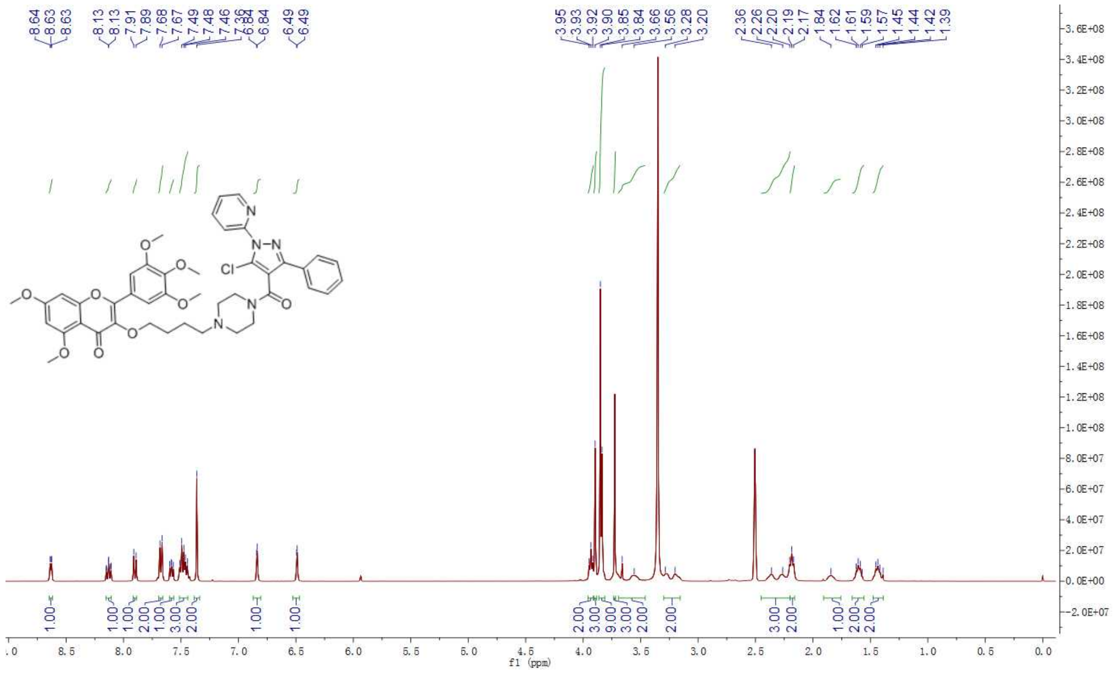

Figure S79 ^{1}H NMR spectrum of title compound **D17**

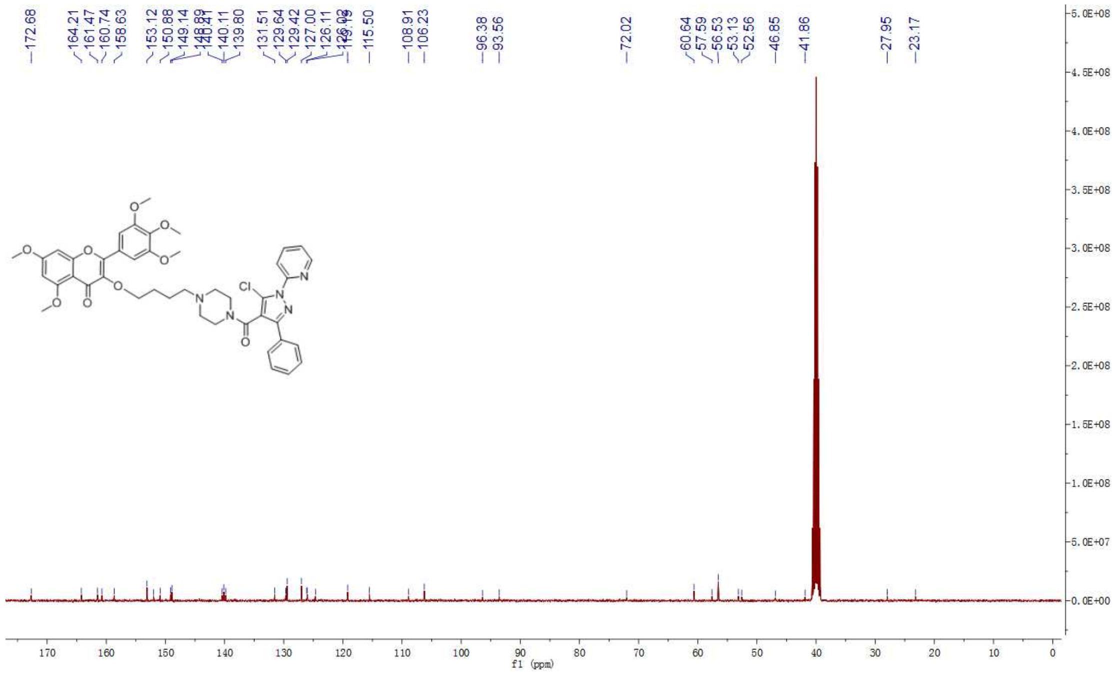

Figure S80 ^{13}C NMR spectrum of title compound **D17**

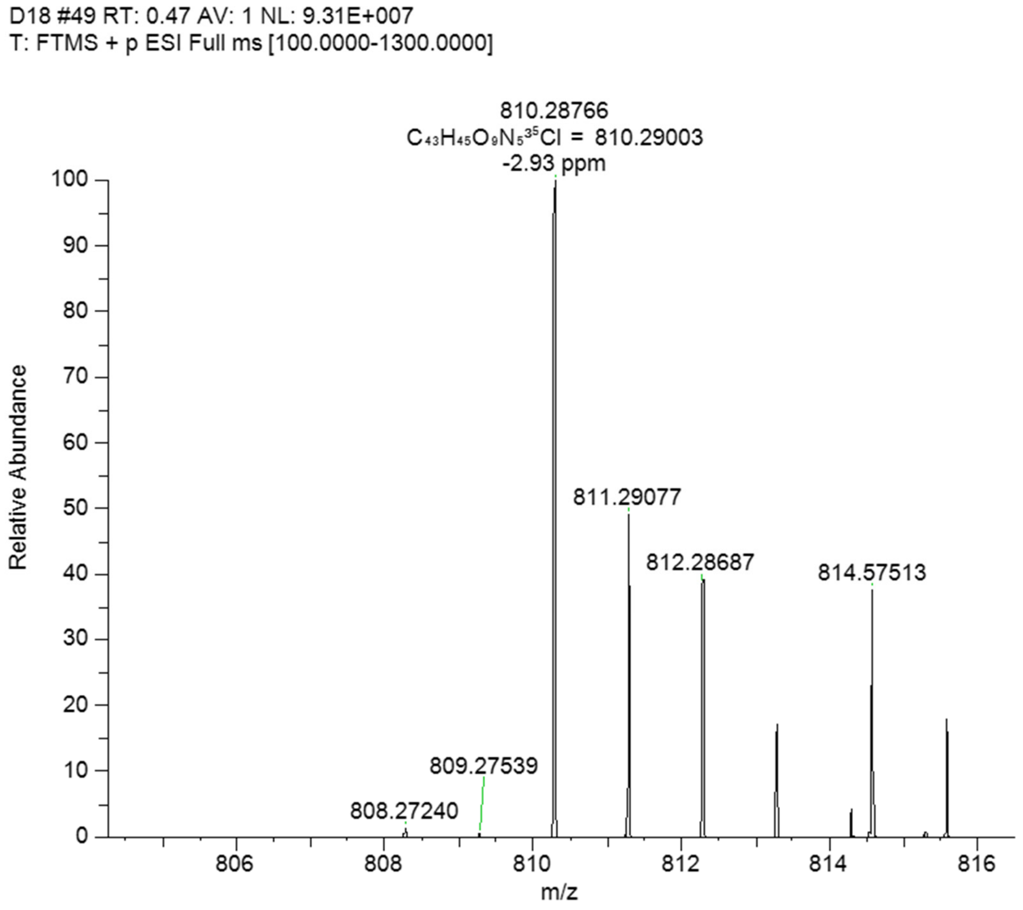

Figure S81 HRMS spectrum of title compound **D17**

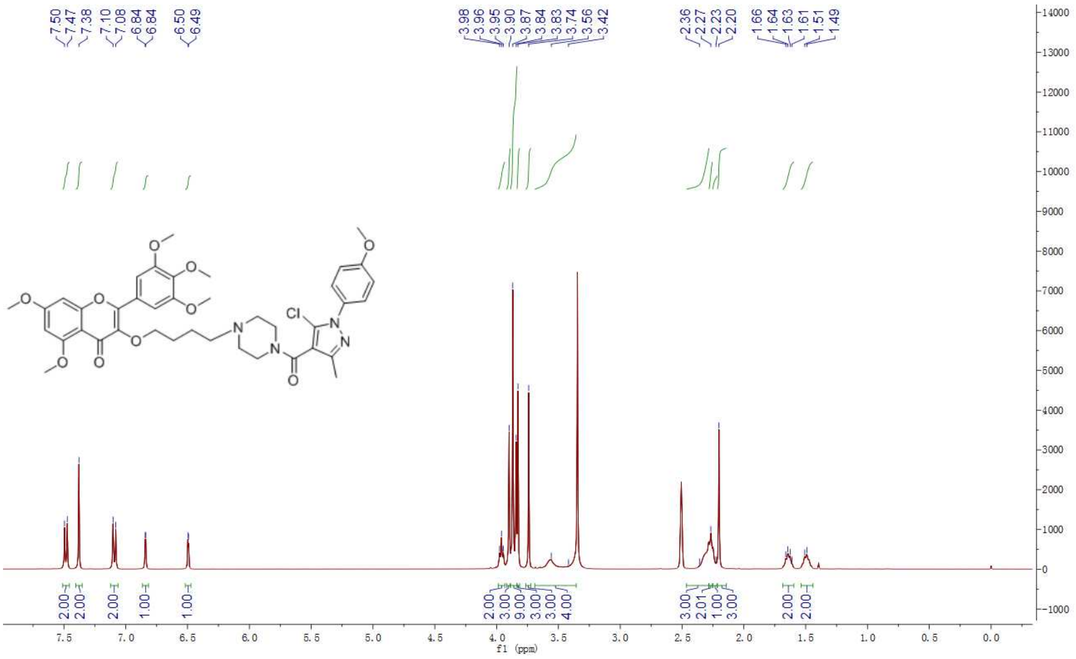

Figure S82 ^{1}H NMR spectrum of title compound **D18**

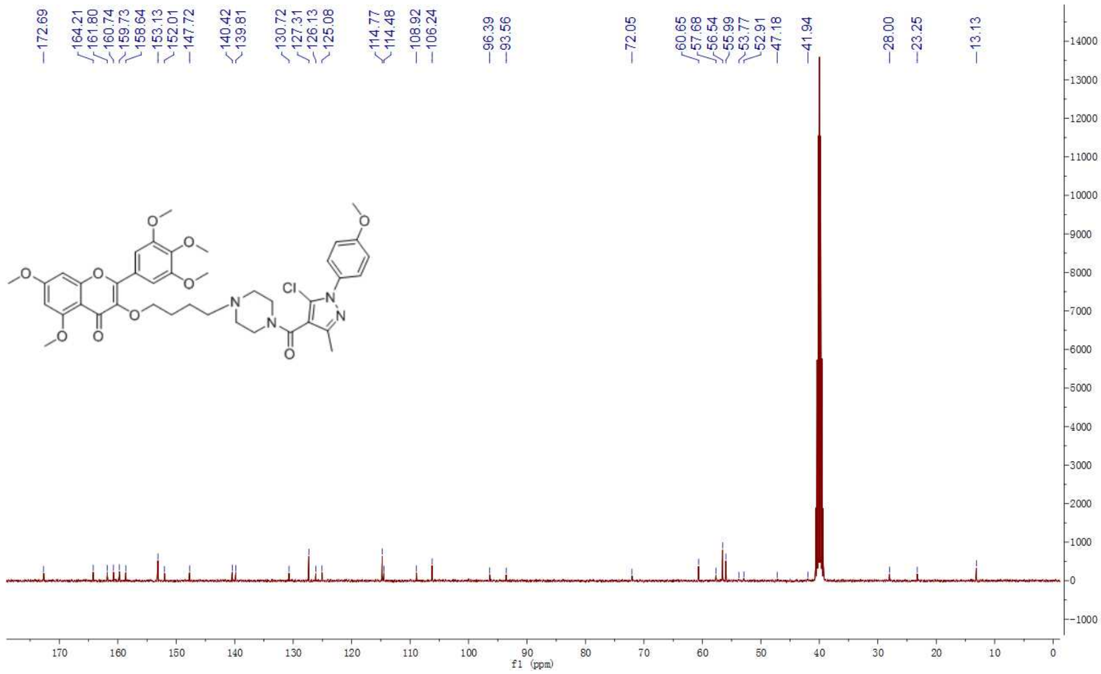

Figure S83 ^{13}C NMR spectrum of title compound **D18**

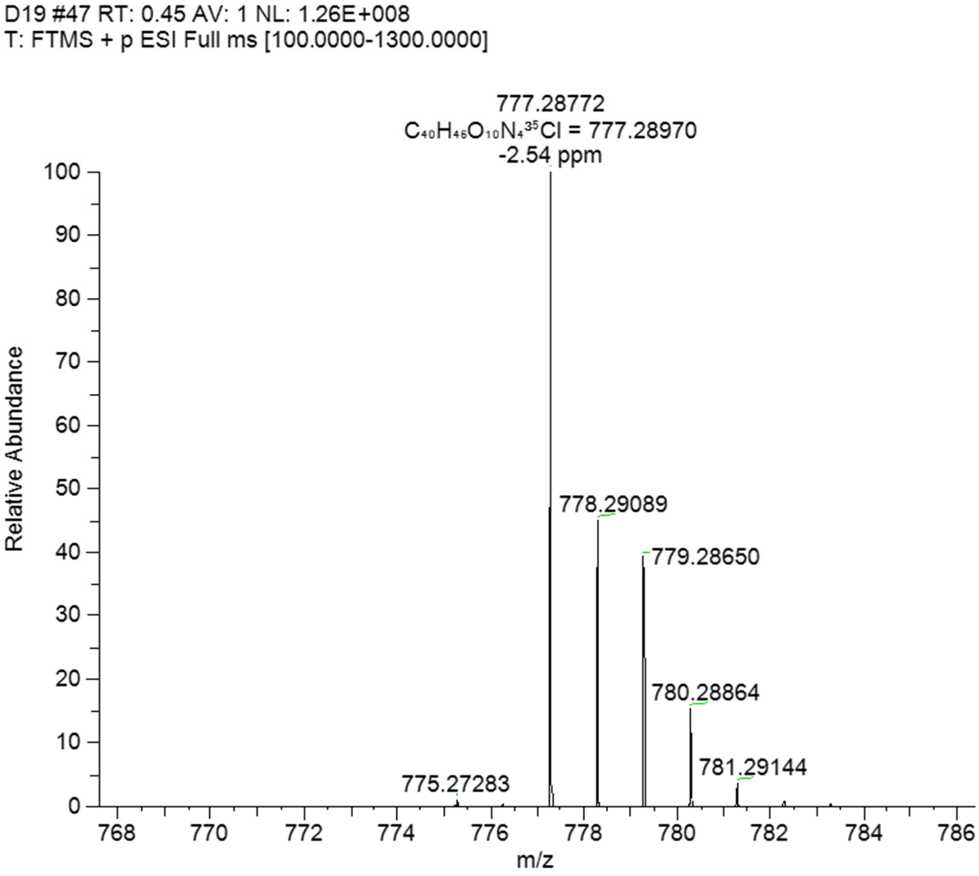

Figure S84 HRMS spectrum of title compound **D18**

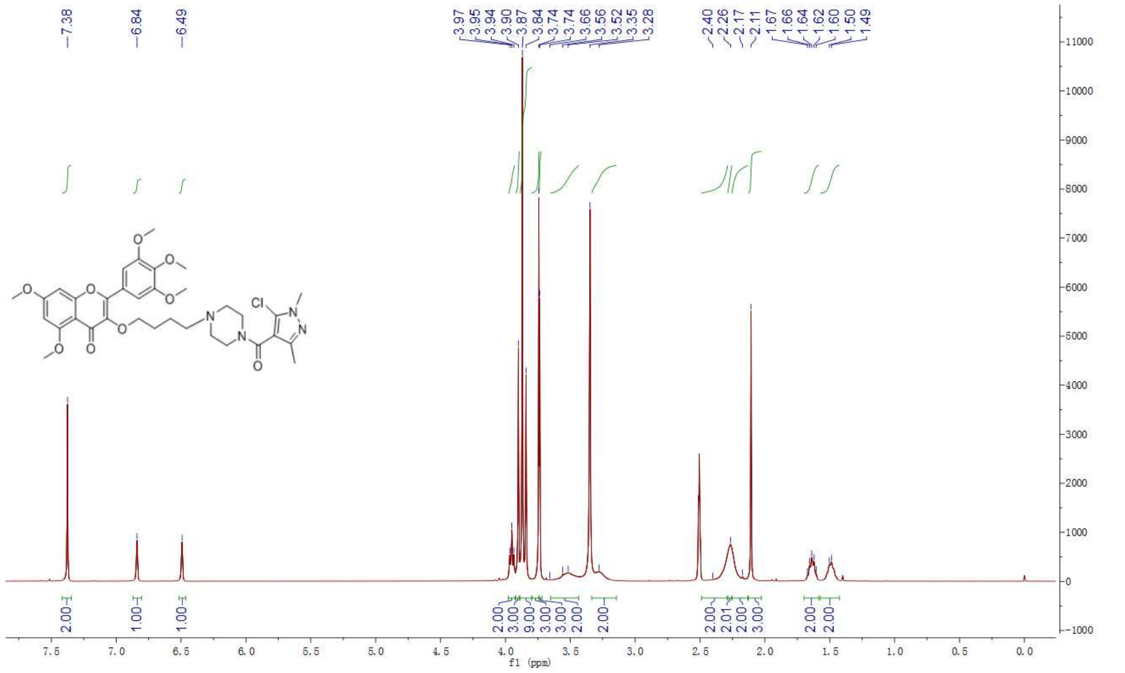

Figure S85 ^{1}H NMR spectrum of title compound **D19**

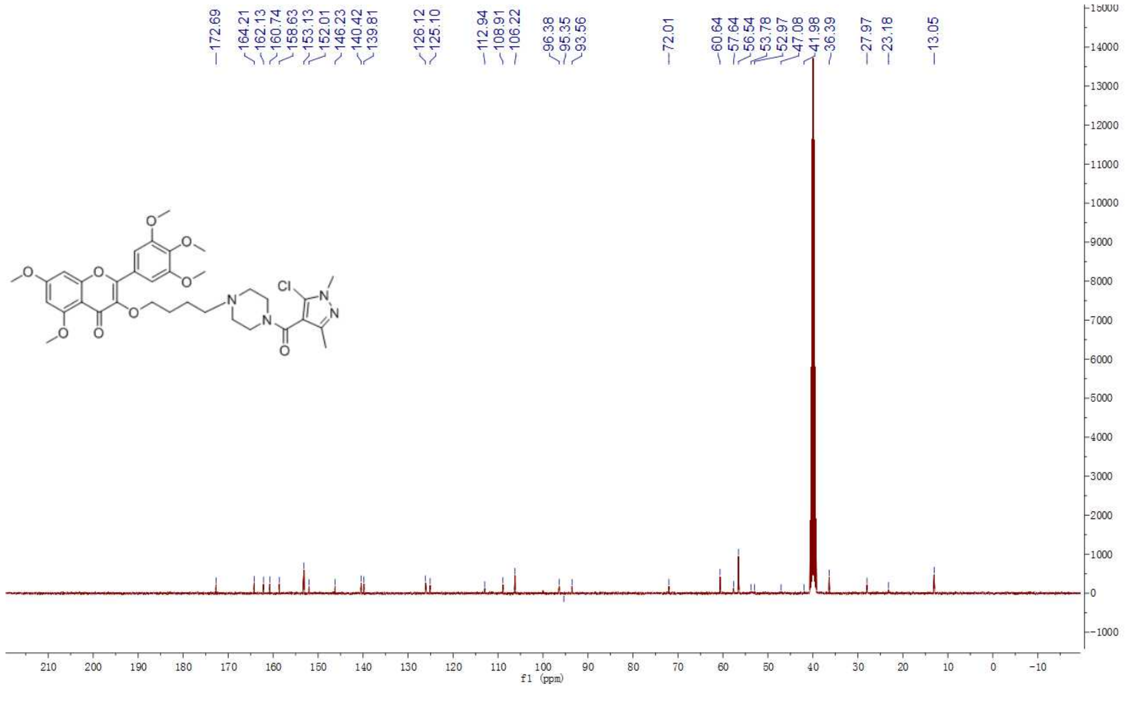

Figure S86 ^{13}C NMR spectrum of title compound **D19**

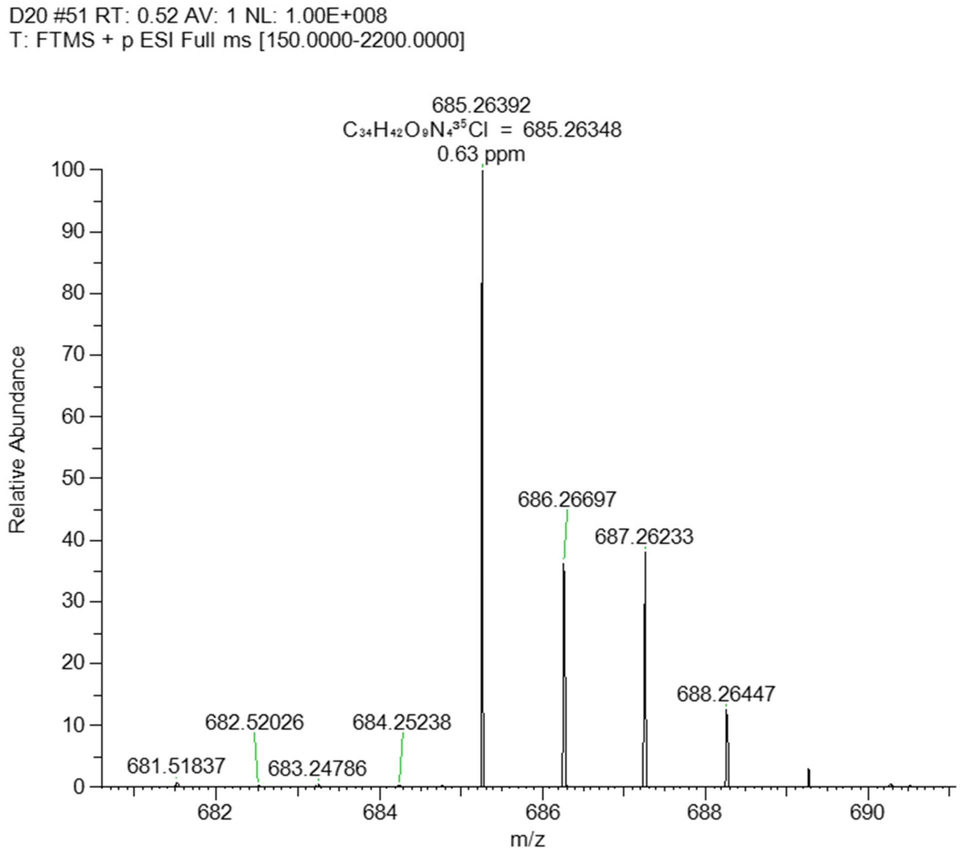

Figure S87 HRMS spectrum of title compound **D19**
